# Using High‐Throughput Screening to Identify Crosslinking Peptides That Control Cell‐Mediated Matrix Degradation

**DOI:** 10.1002/adhm.202501932

**Published:** 2025-08-25

**Authors:** Yingjie Wu, Samuel J. Rozans, Abolfazl Salehi Moghaddam, E. Thomas Pashuck

**Affiliations:** ^1^ Lehigh University 124 E. Morton Street Bethlehem Pennsylvania 18015 United States of America

**Keywords:** cell‐matrix interactions, endothelial cells, high‐throughput, hydrogels, mesenchymal stem cells, proteases

## Abstract

Cells modify the extracellular matrix by expressing proteases that degrade matrix proteins, enabling cell migration within tissues. This process is mimicked in hydrogels through protease‐degradable peptide crosslinks. However, cleaving hydrogel crosslinks reduces local matrix mechanical properties, and most crosslinking peptides, including the widely used GPQGIWGQ “PanMMP” sequence, often lead to bulk hydrogel degradation. Membrane‐type proteases are localized to the cell surface, have important roles in cell migration, and are active in the pericellular region. To identify peptides primarily cleaved by membrane‐type proteases, an approach is developed that couples proteomic identification of candidate peptides with mass spectrometry‐based functional assays to quantify degradation. The target sequence is then optimized using a split‐and‐pool synthesis to generate over 300 peptide variants to improve degradation behavior. The optimized peptide, KLVADLMASAE, shows reduced degradation by soluble proteases, while enabling endothelial and stem cell spreading and viability comparable to PanMMP hydrogels. KLVADLMASAE‐crosslinked hydrogels have reduced crosslinker degradation, are stiffer during culture, and exhibit less macroscopic degradation after 14 days of culture than PanMMP gels. The performance of KLVADLMASAE‐crosslinked gels is significantly improved from the initial peptide target, validating this functional high‐throughput approach for identifying peptides that control matrix degradation.

## Graphical abstract

Protease‐degradable hydrogels mimic dynamic cell‐matrix interactions, but controlling hydrogel degradation is a challenge. We screen libraries of peptides to identify sequences that are primarily degraded by membrane‐localized proteases. Crosslinking hydrogels with these peptides enables cell to spread and migrate while reducing matrix degradation. Utilizing functional screens of hundreds of peptides is a powerful approach for controlling cell‐matrix interactions in hydrogels.

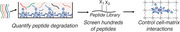

## 1 Introduction

**Figure 1:**
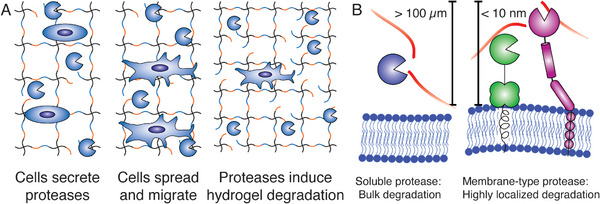
A) Soluble proteases enable cells to spread and migrate within peptide‐crosslinked hydrogels but can also induce bulk degradation. B) Soluble cell‐secreted proteases can diffuse more than 100 µm from the cell surface and induce bulk hydrogel degradation, while membrane‐type proteases typically have enzymatic activity located to the cell surface.

Synthetic hydrogel scaffolds are widely used to model tissues in vitro and regenerate them in vivo.^[^ ^1^, ^2^ ^]^ These gels are often made of crosslinked polymer networks, such as poly(ethylene glycol) (PEG), that offer a high degree of tailorability to the matrix mechanical properties and signaling moieties present within the hydrogels.^[^ ^3^ ^]^ Because many polymers do not degrade on time scales relevant for cell studies, they are frequently crosslinked with peptides that are substrates for cell‐secreted proteases, such as the GPQGIWGQ “PanMMP” peptide that is cleaved by many members of the matrix metalloproteinase (MMP) family of proteases.^[^ ^4^, ^5^ ^]^ Utilizing protease‐cleavable peptides to crosslink hydrogels has the benefit of enabling cell spreading and migration within synthetic matrices (**Figure** **1**A). However, they also introduce variability into hydrogel properties, as the matrix is actively degraded by cells, often leading to bulk degradation by soluble proteases.^[^ ^6^ ^‐^ ^8^ ^]^

The mechanical properties of hydrogels are important for cell behavior^[^ ^9^ ^]^ and these properties are dependent on the extent of crosslinking between polymer chains.^[^ ^6^, ^10^ ^]^ While synthetic matrices are often treated as relatively inert scaffolds that can be modified with specific ligands or properties, the extent to which the encapsulated cells degrade the hydrogel network alters the local network structure.^[^ ^11^ ^]^ The rate of bulk hydrogel degradation can vary by cell type,^[^ ^12^ ^]^ donor age,^[^ ^13^ ^]^ and cell seeding density,^[^ ^14^ ^]^ which introduces experimental variability in the time‐dependent properties of synthetic matrices. A variety of crosslinking peptides have been used to modulate cell‐induced hydrogel degradation, such as increasing or decreasing the rate of bulk hydrogel degradation,^[^ ^5^, ^15^, ^16^ ^]^ controlling cell migration by cell type,^[^ ^12^, ^17^ ^]^ or using a combination of crosslinking peptides to mimic the complex environment found in tissues.^[^ ^18^ ^]^ Endothelial cells are highly sensitive to matrix mechanics, with network formation maximized in matrices that are initially soft and highly degradable but stiffen during culture.^[^ ^19^, ^20^, ^21^, ^22^ ^]^ This can be challenging to mimic within hydrogel matrices, and endothelial cells are frequently co‐cultured with matrix‐producing cells, such as fibroblasts,^[^ ^21^, ^23^ ^]^ or hMSCs,^[^ ^22^, ^24^ ^]^ which both deposit extracellular matrix (ECM) and increase vascular network formation.^[^ ^21^ ^]^ The degradability of peptide crosslinks also influences the osteogenic differentiation of human mesenchymal stem cells (hMSCs).^[^ ^25^ ^]^

Soluble MMPs can diffuse over 100 µm from the surface of cells and induce bulk degradation of hydrogel matrices (Figure 1B).^[^ ^6^, ^7^ ^]^ Protease activity in human tissues is highly regulated to prevent uncontrolled matrix degradation,^[^ ^26^ ^]^ and one mechanism by which cells spatially control degradation is by sequestering proteases to the cell membrane.^[^ ^27^ ^]^ These “membrane‐type” proteases are involved in numerous physiological processes, and there are dozens of proteases that are either permanently located on the cell membrane or are localized there through avidity to membrane proteins.^[^ ^28^, ^29^ ^]^ Membrane‐type proteases, including MMP‐14 (also called MT1‐MMP), enable cell migration through the extracellular matrix in vivo^[^ ^30^ ^]^ while only degrading the pericellular matrix in the immediate vicinity of the cell surface.^[^ ^31^ ^]^ Identifying peptides that are primarily cleaved on the cell surface, rather than by soluble proteases, is a promising method for maintaining hydrogel properties consistent across different cell types, culture periods, and seeding densities, while enabling cell spreading and migration within the matrix. Cells express a complex mixture of proteases^[^ ^26^ ^]^ and most MMP peptides, including the PanMMP peptide, are cleaved by numerous members of the MMP family.^[^ ^5^, ^32^ ^]^ Hydrogels have been crosslinked with peptides that are substrates for membrane‐type proteases.^[^ ^12^, ^15^, ^25^ ^]^ However, most peptides that are cleaved by membrane‐type proteases are also cleaved by soluble proteases, such as MMP‐2^[^ ^15^, ^25^ ^]^ or MMP‐9,^[^ ^12^ ^]^ which limits their ability to reduce bulk hydrogel degradation.

In this work, we used a proteomics‐based approach to identify novel protease‐substrate peptides. We then used a functional cell‐based degradation assay to select a peptide that is primarily degraded in cell culture only when cells are present, but not in cell‐conditioned media that contains soluble proteases. These peptides were candidates for membrane‐specific degradation. A split‐and‐pool synthesis scheme was used to generate over 300 variants of the most promising membrane‐specific peptide to improve the degradation kinetics while maintaining specificity for degradation when cells are present. Human mesenchymal stem cells and human umbilical vein endothelial cells (hUVECs) were studied due to their biological relevance, and both stem cells and endothelial cells have significant therapeutic importance. The optimized peptides were used as crosslinks for hydrogels, and we determined that a KLVADLMASAE peptide enables both hMSCs and hUVECs to spread in the gels with average cell area and viability equivalent to that observed with the PanMMP peptide, but significantly improved over the initial peptide identified in the proteomics screen. Hydrogels crosslinked by the KLVADLMASAE were less degraded and were stiffer during culture than PanMMP hydrogels, indicating that our soluble peptide screen effectively predicted how the peptides were degraded when used as crosslinking peptides. Since all cell types express a different mix of proteases, and time‐dependent changes to the matrix structure is important for cell behavior, this work introduces a functional approach to controlling time‐dependent changes to the matrix properties of synthetic hydrogels.

## 2 Results and Discussion

### 2.1 Proteomics‐Based Approach to Peptide Discovery

We utilized a proteomics‐based approach to identify protease substrate peptides and then developed a novel functional assay to quantify how quickly these peptides are degraded. Terminal amine isotopic labeling of substrates (TAILS) is a proteomics technique that identifies sites of proteolytic degradation by labeling the resulting N‐terminal amines with dimethylation, a non‐natural chemical modification.^[^ ^33^, ^34^ ^]^ These dimethylated N‐termini can then be identified in liquid chromatography mass spectrometry (LC‐MS) and are candidates for sites of proteolytic cleavage. Candidate peptides found in the TAILS screen were then synthesized with the 4‐5 amino acids flanking the N‐ and C‐terminal sides of the likely site of proteolytic cleavage. Therapeutic peptide drugs frequently undergo non‐specific degradation by exopeptidases,^[^ ^35^ ^]^ which are proteases that cleave amino acids off the N‐ or C‐termini of peptides. Modifying the termini of peptides with an acetylated β‐alanine on the N‐terminus and an amidated β‐alanine on the C‐terminus has been shown to greatly limit non‐specific peptide degradation and enable the quantification of peptide degradation by LC‐MS,^[^ ^36^ ^]^ and all soluble peptides in this work had these modifications.

**Figure 2:**
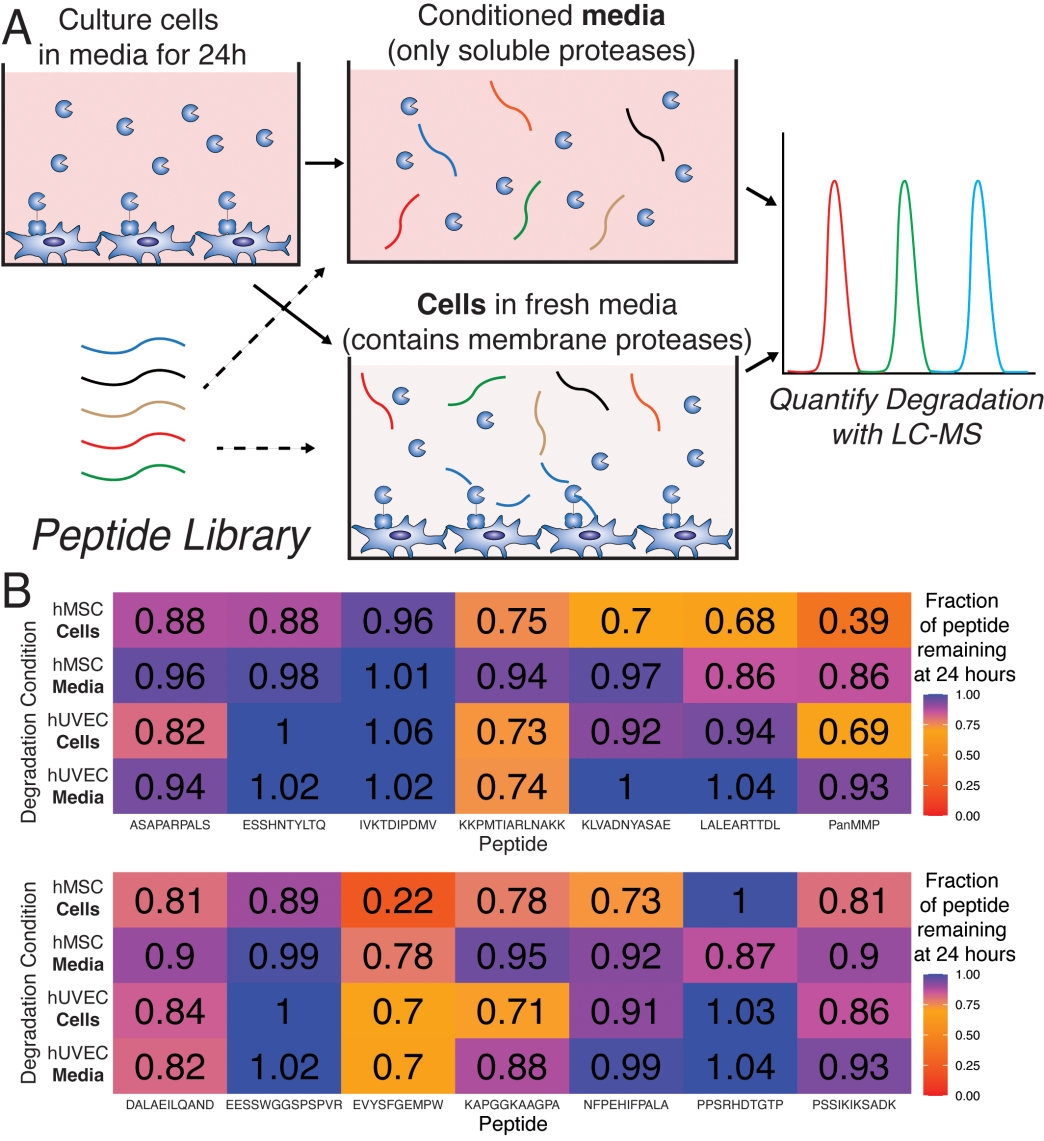
A) We used a functional approach to quantify how peptides are degraded by both cells in media (Cells) and conditioned media (Media). Since membrane‐type proteases should only be found on cells, peptides that are primarily degraded in the Cells condition are candidates for relative specificity to membrane‐type proteases. B) We found that the commonly used PanMMP showed degradation in both Cells and Media conditions, while the KLVADNYASAE peptide showed minimal degradation in the Media condition. Error bars indicate ± standard deviation, N = 5.

Most in vitro protease activity assays incubate the peptides or proteins of interest with a single protease in a buffered solution. However, the kinetics of protease activity in tissues are complex and highly regulated, and both the activity^[^ ^37^ ^]^ and substrate specificity^[^ ^38^ ^]^ of proteases can be modulated through association with other proteins.^[^ ^39^ ^]^ Most MMP‐substrate peptides are also cleaved by multiple proteases,^[^ ^5^ ^]^ and within the context of biomaterials, the most important factor is typically how the combined activity of all proteases, rather than the activity of any specific protease, modifies the local microenvironment versus the activity due to any individual enzyme. We synthesized peptides identified in the TAILS proteomic screen that were present on proteins that are known to be secreted or found on the cell membrane, pooled them, and added them either to cell culture media on top of cells, the “Cells” condition, or conditioned media lacking cells, the “Media” condition (**Figure** **2**A). The fraction of peptides remaining after 24 h in each condition was then quantified using LC‐MS. Soluble proteases should be present in both conditions, however, membrane‐type proteases should primarily be found in the samples containing cells and not the conditioned media samples. A key benefit of this approach is that it surveys the total functional proteolytic activity, including the effects of inhibitors and co‐factors, and quantifies the degradation rate of soluble peptides by the complete secretome of different cell types.

We tested our initial peptide library on both hMSCs and hUVECs because of their biomedical relevance, and their significantly different protease expression profile^[^ ^40^, ^41^ ^]^ increases the likelihood that a peptide that is membrane‐specific in both cell types is more likely to be broadly applicable across cell types. We found that peptides had varying degradation rates by both cell type and Cell/Media condition. In Figure 2B our data shows that some peptides, such as the IVKTDIPDMV sequence, had minimal degradation under any conditions, while both the KKPMTIARLNAKK peptide and PanMMP peptide were significantly degraded in both hMSCs and hUVECs in both the Cells and Media conditions (*p* < 0.05). The KLVADNYASAE was identified as a peptide sequence that was cleaved by both hMSCs and hUVECs, but was primarily cleaved only when cells are present and not in conditioned media, having less than 3% degradation in Media condition for either cell type.

While the KLVADNYASAE peptide had 70% remaining after 24 h in the Cells condition for hMSCs, the amount of degradation in the Cells condition was lower than for the PanMMP peptide (61% PanMMP, p <0.05). Furthermore, the KLVADNYASAE peptide had only minor degradation in the Cells condition for hUVECs, with 90% remaining. Since the final goal of these peptides is to crosslink hydrogels and enable cells to spread and migrate, it is desirable to have peptides that are cleaved under the Cells condition as rapidly as possible to ensure that the spreading and migration processes are not hindered by the inability of cells to degrade the local matrix. The rate at which a specific peptide is degraded by a protease is highly sensitive to the amino acids near the cleavage site,^[^ ^42^ ^]^ and mutating amino acids near this site can significantly change the rate of protease activity.^[^ ^32^ ^]^

### 2.2 Split‐And‐Pool Synthesis of Peptide Libraries to Improve Protease Kinetics

**Figure 3:**
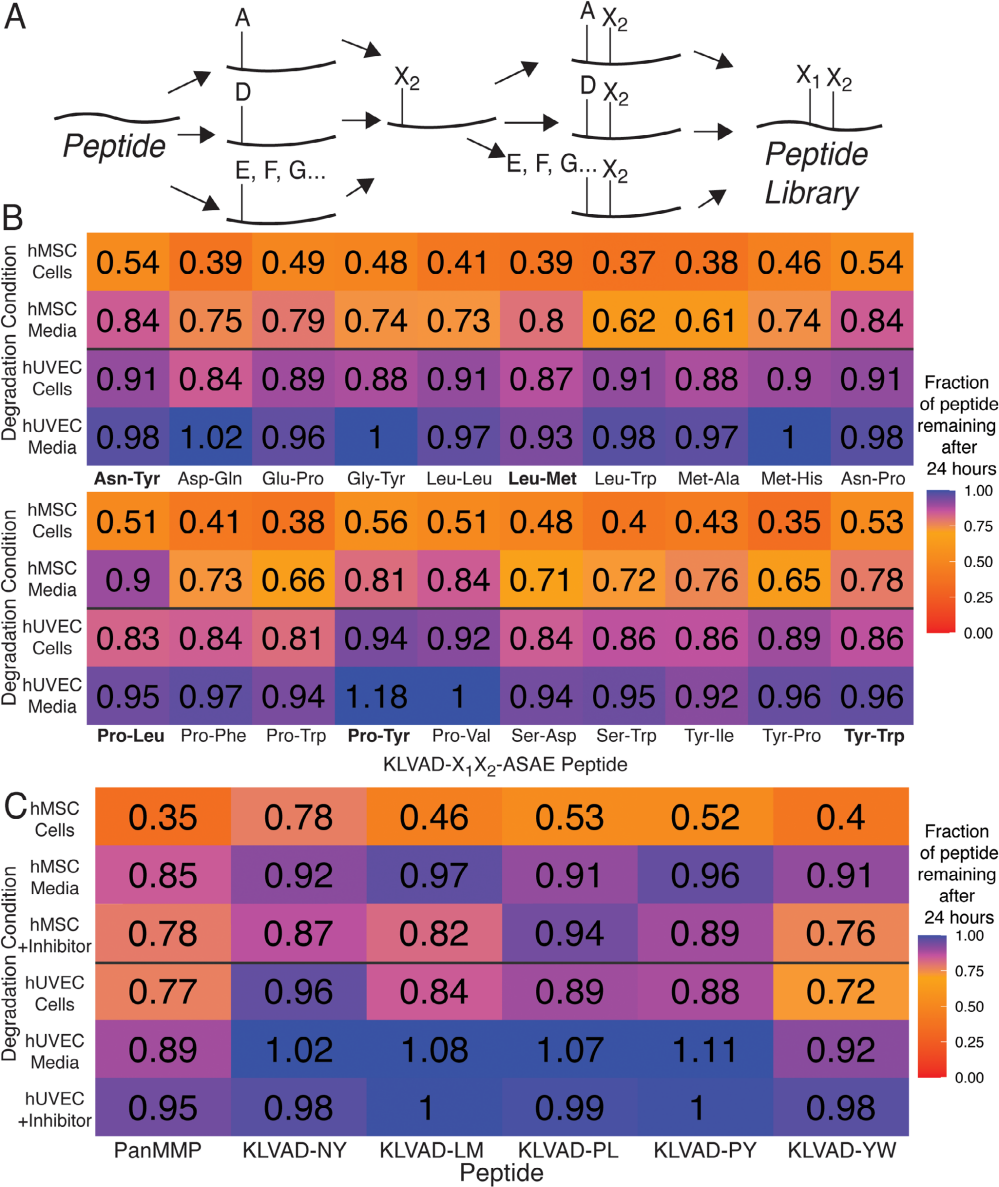
A) A split‐and‐pool approach was used to tune peptide degradation kinetics by making more than 300 variants of the KLVADNYASAE peptide having the structure KLVADX_1_X_2_ASAE. B) Modifying the amino acids near the site of proteolytic cleavage tuned the degradation kinetics for both hMSCs and hUVECs (N = 2). C) The most promising peptides from the split‐and‐pool studies were cultured with hMSCs and hUVECs, and the amount of peptide degradation in both the Cells and Media was quantified using LC‐MS. Peptides were also added to the Cells condition that contained an MMP‐14 inhibitor. KLVAD peptides showed significant specificity for degradation in the cell condition, and the addition of an MMP‐14 inhibitor greatly reduced the peptide degradation rate. (N = 6).

Our proteomics‐based method for identifying peptide sequences is limited to the protease‐accessible parts of proteins secreted by cells, which constitutes just a minuscule fraction of the ≈10^13^ possible 10 amino acid sequences. We used a “split‐and‐pool” approach^[^ ^43^ ^]^ to synthesize more than 300 peptide variants of the KLVADNYASAE peptide first identified in our proteomics screen (**Figure** **3**A). Previous studies have shown that the enzyme kinetics of membrane‐type MMPs, including MMP‐14, are highly sensitive to the amino acids near the site of cleavage.^[^ ^44^, ^45^ ^]^ We mutated the P1’ and P2’ locations on the peptide, which are the two amino acids on the C‐terminal side of the cleavage site, resulting in the format KLVADX_1_X_2_ASAE. Peptides were synthesized on solid‐phase resin consisting of 10^5^–10^6^ particles per gram of resin, and upon addition of the ASAE amino acids, the resin was split 19 ways. Each canonical amino acid except cysteine was added to one of the 19 fractions. These were then pooled together into a single batch containing all 19 variants, and then split 19 ways again to create more than 300 variants having the sequence KLVADX_1_X_2_ASAE, where X_1_ and X_2_ can each be of the 19 different amino acids. The second split of 19 different variants was then pooled into 10 different libraries, and the remaining KLVAD amino acids were added to each of the libraries. The ten peptide libraries containing either 19 or 38 peptides were incubated with hUVECs and hMSCs in the Cells and Media conditions, and the degradation of each peptide in the libraries was quantified using LC‐MS.

The peptides in the libraries were evaluated with two primary considerations: having maximal degradation in the “Cells” condition that contains membrane‐proteases, and minimal degradation in the “Media” condition, which should have minimal membrane‐type protease activity. The ability of cells to degrade covalent matrices is important for spreading and viability,^[^ ^5^ ^]^ and membrane‐type proteases degrade the pericellular matrix during cell migration through the ECM.^[^ ^30^ ^]^ An ideal crosslinking peptide should be degraded by both hMSCs and hUVECs, like the PanMMP peptide, so that it can be used across different cell types. Minimizing degradation in the Media condition is important to limit unwanted bulk hydrogel degradation. Figure 3B shows the subset of split‐and‐pool peptide library that had minimal degradation in the hUVEC Media condition (>93% remaining after 24 h), but had significant degradation in the hMSC Cells condition (<54% of peptide remaining after 24 h), and also included the Asn‐Tyr peptide from KLVAD‐**NY**‐ASAE, the peptide sequence from which the split‐and‐pool library was derived. Peptides were further refined by selecting for those that had minimal degradation in hMSC (>77% of peptide remaining). From this smaller pool the following peptides were selected, KLVAD‐**LM**‐ASAE, KLVAD‐**PL**‐ASAE, PLVAD‐**PY**‐ASAE, and KLVAD‐**YW**‐ASAE, in addition to the parent peptide, KLVAD‐**NY**‐ASAE, for further characterization.

The most promising peptides identified in the split‐and‐pool study were then synthesized using standard peptide synthesis protocols and cultured with hMSCs and hUVECs at a higher concentration of 50 µM in both the Cells and Media conditions. The KLVAD‐derived peptides had limited degradation in the Media condition, with more than 90% remaining, and in most cases, more than 95% remaining (Figure 3C). These results indicate that the split‐and‐pool approach successfully improved the degradation kinetics of the peptide identified in the proteomic screen. For instance, the KLVAD‐**LM** peptide had increased degradation compared to the KLVAD‐**NY** peptide in the Cells condition for both hMSCs (46% remaining versus 78% remaining, *p* < 0.05), and also having more than 94% remaining in the Media conditions for either hUVECs or hMSCs. The PanMMP peptide shows significant cleavage in the Media condition, which contains soluble proteases for both hMSCs and hUVECs. These results indicate that the initial proteomic peptide screen was able to identify a peptide with the desired degradation specificity and that the subsequent split‐and‐pool screen improved peptide degradation under desired conditions while minimizing degradation in undesirable conditions.

The extent of peptide degradation in the Cells condition is increased compared to the Media condition across all peptides. Part of this difference is likely due to membrane‐type proteases localized to the cell surface. However, it is also possible that the activity of secreted proteases decreases over time in the Media condition since they are no longer getting produced by a local population of cells. To better understand time‐dependent changes in proteolytic activity, we performed a degradation study and compared peptide degradation in the media in the for the first 24 h after being removed from contact with cells, to 24–48 h (Figure S1, Supporting Information). The KLVAD‐LM peptide had greater than 90% remaining across all Media conditions, with only minimal differences between 0–24h culture and 24–48 h culture. We found that there was statistically significant degradation for the PanMMP peptide in the media condition for the hMSCs at both 0–24 h and 24–48 h after culture with cells (*p* < 0.05). The amount of PanMMP degradation from 24–48 h trended lower than 0–24 h for both hMSCs and hUVECs, but the results were not statistically significant. These results show that some proteases in the conditioned media remain proteolytically active more than 24 h after being secreted by cells.

A key benefit of a functional approach to quantifying degradation during culture is that the kinetics of degradation can be tuned without any prior knowledge of protease expression profiles. A consequence of this approach is that the identity of the specific protease(s) that are cleaving the peptides is completely unknown. hMSCs^[^ ^40^, ^46^, ^47^ ^]^ and hUVECs^[^ ^41^ ^]^ express numerous membrane‐type proteases, including the membrane protease MMP‐14. MMP‐14 is also widely expressed across cell types,^[^ ^30^ ^]^ where it has roles in migration, differentiation,^[^ ^48^, ^49^ ^]^ and angiogenesis.^[^ ^50^, ^51^ ^]^ This makes MMP‐14 a likely candidate for catalyzing peptide degradation in the presence of cells.

To better understand the quantity and localization of MMP‐14 in hMSCs and hUVECs in culture, we performed an enzyme‐linked immunosorbent assay (ELISA) to quantify the concentration of MMP‐14 in both conditioned media and on cells. hMSCs and hUVECs were cultured in media for 24 h, at which point the MMP‐14 concentration in both the cells and conditioned media was quantified (Figure S1, Supporting Information). hMSCs and hUVECs secrete extracellular vesicles (EVs), and MMP‐14 has been shown to be present on the surface of EVs.^[^ ^52^ ^]^ EVs can be depleted from conditioned media using ultracentrifugation,^[^ ^53^ ^],^ and centrifuged the conditioned media to remove EVs and quantify the presence of MMP‐14 as a soluble protein. We found that hMSCs had greater amounts of MMP‐14 than hUVECs (12.6 and 7.5 ng mL^−1^, p< 0.001) (Figure S2, Supporting Information). Both hMSCs and hUVECs had less than 1.2 ng mL^−1^ of MMP‐14 in conditioned media. The concentration of MMP‐14 in conditioned media and the ultracentrifuged samples was nearly identical for both hMSCs and hUVECs, suggesting a negligible amount of MMP‐14 is present on secreted EVs.

We then added the MMP‐14 inhibitor NSC 405 020^[^ ^54^ ^]^ to both the Cells and Media condition (without additional MMP‐14) to quantify the effect that cell‐produced MMP‐14 has on peptide degradation. NSC 405 020 binds a hemopexin domain on MMP‐14, which inhibits the ability of MMP‐14 to homodimerize, reducing the proteolytic activity.^[^ ^54^ ^]^ Unlike the catalytic site, the hemopexin domain on MMPs is largely unique to each family member.^[^ ^55^ ^]^ NSC 405 020 has not been reported to have significant cross‐reactivity with other MMPs, and most soluble MMPs do not require dimerization for activity, however, there are not reports of it being tested against most family members or non‐MMP proteases, so it is possible that its inhibitory activity is not limited to MMP‐14.

We found that adding NSC 405020 to the Cells condition led to a reduction in protease degradation of all peptides across both cell types (Figure 3). Interestingly, while most peptides still had greater than 10% degradation in the Cells + Inhibitor condition for hMSCs, only the PanMMP had greater than 2% degradation after 24 h when cultured with hUVECs. These results indicate that MMP‐14 is possibly responsible for almost all peptide degradation in the hUVEC Cells condition, while the partial reduction of proteolytic degradation in the hMSC Cells condition suggests that both MMP‐14 and other proteases are responsible for peptide degradation. It is also notable that the MMP‐14 inhibitor reduced PanMMP degradation in hMSCs from 35% remaining to 78% remaining, and in hUVECs from 85% remaining to 95% remaining, indicating that MMP‐14 plays a major role in cell‐based degradation of the PanMMP sequence, even though it is known to be cleaved by many other MMPs. This indicates that while both peptides are cleaved by more than one protease, a larger fraction of protease activity against the KLVAD peptides is due to the membrane‐type MMP‐14 protease.

### 2.3 Cell Growth and Viability within Peptide‐Cross Linked Hydrogels

**Figure 4:**
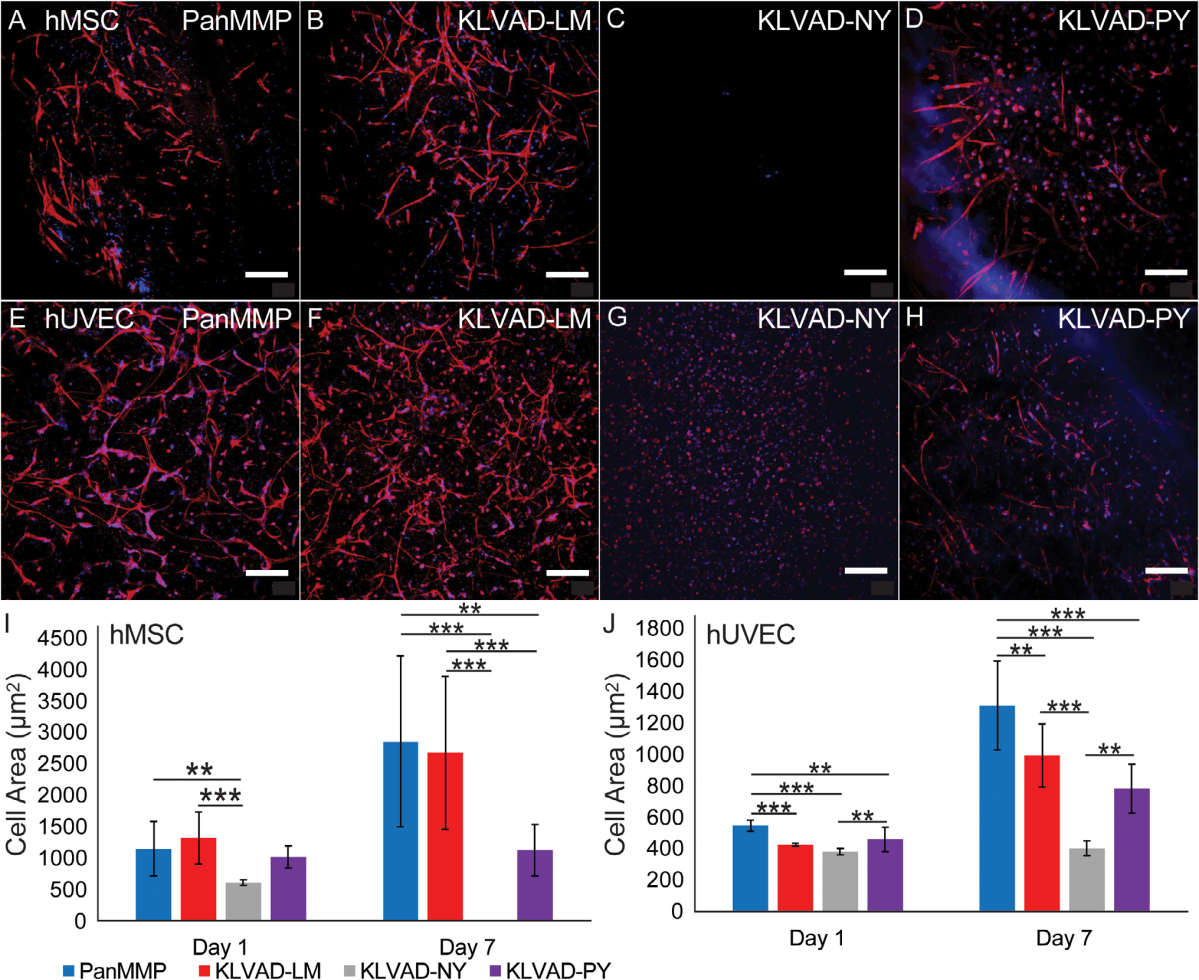
Cells were grown for seven days in PEG hydrogels with different crosslinking peptides. hMSCs in hydrogels crosslinked by the A) PanMMP, B) KLVAD‐LM, C) KLVAD‐NY, and D) KLVAD‐PY peptides, and hUVECs cultured in hydrogels crosslinked by the E) PanMMP, F) KLVAD‐LM, G) KLVAD‐NY, and H) KLVAD‐PY peptides. The average cell area was quantified at 1 and 3 days for I) hMSCs and J) hUVECs. Scale bar is 100 µm. ^*^ indicates *p* < 0.05, ^**^ indicates *p* < 0.01, and ^***^ indicates that *p* < 0.001 by Tukey's post‐hoc test. Error bars indicate ± standard deviation, N = 3 with technical triplicates.

Each of the peptides was then used to crosslink hydrogels containing hMSCs or hUVECs at 20 million cells mL^−1^ and cultured for 1, 3, or 7 days. To maximize cell spreading, we made soft hydrogels that were 3.5% 8‐arm poly(ethylene glycol) (PEG) by weight. 40% of the arms were reacted with the crosslinking peptides, and 10% of the arms were functionalized with a cyclic RGD peptide for cell adhesion. We found that both the PanMMP peptide and KLVAD‐**LM** supported cell spreading and growth within the hydrogels (**Figure** **4**). In a surprising result, hydrogels crosslinked by the original KLVAD‐**NY**‐ASAE peptide were found to be cytotoxic, and by Day 7 there were no detectable cells within the hMSC‐seeded gels, and hUVECs showed only minimal cell spreading. The KLVAD‐**PY** peptide also had less cell spreading and viability compared to the PanMMP and KLVAD‐**LM** peptides for both hMSCs and hUVECs (Figure 4I,J). Interestingly, the addition of an MMP‐14 inhibitor completely prevented hUVEC spreading from increasing between Day 1 and Day 7 in a KLVAD‐**LM**‐crosslinked hydrogel, but hUVECs in a PanMMP‐crosslinked hydrogel were still able to spread (Figures S3 and S4, Supporting Information). This supports the inhibitor studies for the soluble peptides in which the MMP‐14 inhibitor completely abolished the degradation of the KLVAD‐**LM** peptide by hUVECs, but not the PanMMP peptide. (Figure 3C). It also shows that degradation studies using soluble peptides were predictive of cell spreading and viability within peptide crosslinked hydrogels.

Cell viability was also found to be highly dependent on the sequence of the peptide crosslinking the PEG hydrogels. hMSCs cultured in hydrogels crosslinked by either the PanMMP or KLVAD‐**LM** peptide had significantly higher viability after seven days of culture than the KLVAD‐**NY** or KLVAD‐**PY** peptide, based on either metabolic activity assays (Figure S5, Supporting Information) or cell number (Figure S5, Supporting Information). Notably, the KLVAD‐**LM**‐ASAE peptide is not found within the human proteome and thus cannot be identified through a proteomics‐only approach, and this highlights the importance of utilizing a split‐and‐pool methodology for improving the biological performance of crosslinking peptides identified from physiological protease‐substrate interactions. hUVECs cultured in gels crosslinked by the PanMMP peptide had higher metabolic activity after seven days than the KLVAD peptides (Figure S5, Supporting Information), although the difference in cell number was not significant (Figure S5, Supporting Information). hUVEC growth is optimized in very weak, highly degraded hydrogels,^[^ ^20^ ^]^ so the propensity of the PanMMP peptide to undergo bulk degradation could be advantageous for this cell type.

### 2.4 Quantification of Peptide Degradation During Culture

**Figure 5:**
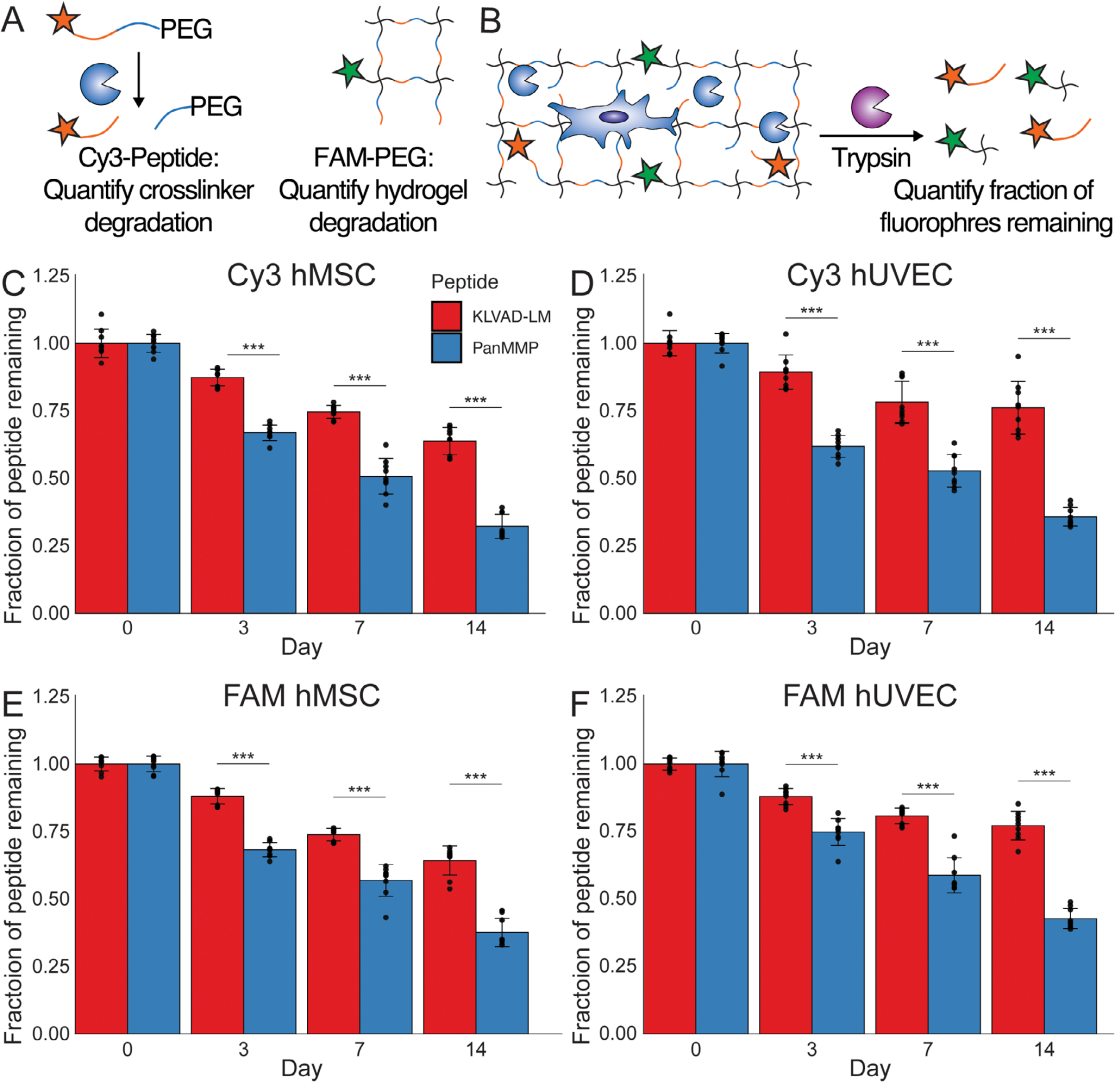
The rate of degradation of crosslinking peptides in the hydrogels was quantified using fluorescence. A) A fraction of PEG arms were functionalized with PanMMP and KLVAD‐LM peptides that were labeled with fluorescein on their N‐terminus and conjugated to the PEG hydrogel matrix on their C‐terminus to quantify crosslinker degradation. Fluorescein (FAM) was also conjugated to the hydrogel through non‐degradable linkers to quantify bulk hydrogel degradation. B) hUVECs and hMSCs were grown in gels in which a small fraction PEG arms was conjugated of fluorescently‐labeled peptides. After 0, 3, and 7 days the gels were washed and then digested with trypsin. The amounts of remaining Cy3 and FAM were quantified using fluorescence spectroscopy. The extent of peptide degradation was quantified for C) hMSCs and D) hUVECs, and the extent of hydrogel degradation was quantified for E) hMSCs and F) hUVECs, with all gels containing cells cultured at 20 million cells mL^−1^.^**^ indicates *p* < 0.01, and ^***^ indicates that *p* < 0.001 by a Student's *t*‐test. Error bars indicate ± standard deviation, N = 3 with technical triplicates.

Next we designed systems to quantify the amount of crosslinking peptide degradation and bulk hydrogel degradation during culture. We synthesized both the KLVAD‐LM peptide and the PanMMP peptide with an N‐terminal Cy3 and a C‐terminal azide to click into the PEG hydrogel network, and also coupled fluorescein (FAM) to the hydrogel using a non‐degradable linker (**Figure** **5**A). Proteolytic cleavage of the crosslinking peptides will liberate the Cy3 from the PEG hydrogel, and bulk erosion of the hydrogel reduces the amount of PEG and FAM. At desired time points, the gels were completely degraded with trypsin, and the fluorescence intensity of Cy3 and FAM was measured to quantify peptide and hydrogel degradation during culture (Figure 5B).

We found that the KLVAD‐LM peptide had reduced degradation compared to the PanMMP peptide for both hMSCs and hUVECs at 20 million cells mL‐1 all time points (Figure 5; Figure S6, Supporting Information). After fourteen days of culture with 20 million cells mL^−1^ we found that in hydrogels crosslinked by the PanMMP peptide, 36 and 32% of peptides remained for hUVECs and hMSCs, respectively. Hydrogels crosslinked by KLVAD‐LM had 76 and 64% of peptides remaining, respectively, and the differences between PanMMP and KLVAD‐LM‐crosslinked hydrogels were statistically significant (*p* < 0.001). Bulk degradation of the hydrogel was quantified by measuring FAM fluorescence, and we found that PEG gels crosslinked by PanMMP had a lower fraction of PEG remaining after 14 days for both hUVECs (43%) and hMSCs (38%) than KLVAD‐LM‐crosslinked hydrogels for both hUVECs (77%) and hMSCs (64%). We also found that the amount of peptide remaining was lower for PanMMP hydrogels compared to KLVAD‐LM hydrogels when hUVECs and hMSCs were added at 1 million cells mL^−1^ (Figure S6A,B, Supporting Information). The difference in degradation for hMSCs was noticeable on a macroscopic level, and PanMMP hydrogels were noticeably degraded after seven days, while KLVAD‐LM hydrogels had no observable changes from Day 0 (Figure S6C,D, Supporting Information).

It is notable that the initial peptide screens were done as soluble peptides incubated over cells on tissue culture plastic, while in the hydrogel studies the cells were in a different 3D environment, and the peptides were covalently conjugated to the polymer matrix at both termini, which greatly reduced their molecular mobility. Importantly, the degradation behavior of the crosslinking peptides within the hydrogel was effectively predicted by the soluble peptide studies, but only when considering degradation in both the presence and absence of cells (the “Cells” and “Media” conditions, respectively). The KLVAD‐LM and PanMMP peptides had similar degradation in the Cell condition (35% versus 46% remaining after 24 h for hMSCs, 77% and 84% for hUVECs, respectively), and while the KLVAD‐LM had negligible degradation in the Media condition, the PanMMP had 85% remaining for hMSCs and 89% for hUVECs. Within hydrogels, the KLVAD‐LM peptide showed substantial degradation, unlike the Media condition with soluble peptides, however, its degradation by hMSCs and hUVECs was only 40–60% of that seen with the PanMMP peptide. This represents a much larger difference than was observed between the peptides in the soluble “Cells” condition.

### 2.5 Rheological Characterization of Hydrogels During Culture

**Figure 6:**
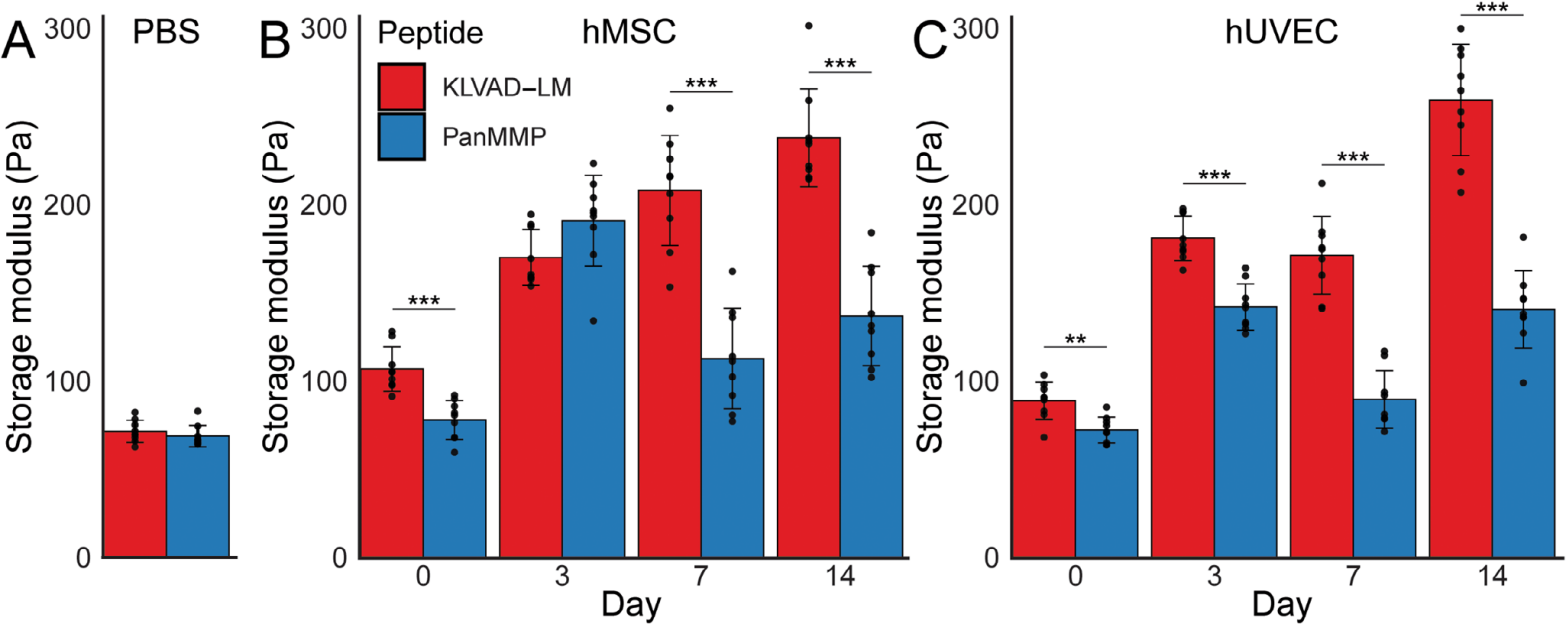
Rheology was performed on hydrogels before and after cell culture with hMSCs and hUVECs. A) The storage moduli of the KLVAD‐LM peptide (71 ± 6 Pa) and PanMMP (69 ± 6 Pa) were similar for hydrogels formed in PBS without cells. B) Cells deposit matrix during culture, and we found that hydrogels with hMSCs stiffen during culture, however, this was peptide dependent. KLVAD‐LM hydrogels had an increase in storage modulus from 89 ± 10 Pa at Day 0 to 250 ± 32 Pa at Day 14 (*p* < 0.001), while PanMMP hydrogels only stiffened from 78 ± 11 Pa to 141 ± 22 Pa (*p* < 0.001). C) This trend was also seen in hUVECs, where the storage modulus of KLVAD‐LM‐crosslinked hydrogels increased from 89 ± 10 Pa to 260 ± 32 Pa (*p* < 0.001) and the storage modulus of PanMMP crosslinked hydrogels increased from 73 ± 7 Pa to 141 ± 22 Pa (*p* < 0.001) ^*^ indicates *p* < 0.05, ^**^ indicates *p* < 0.01, and ^***^ indicates that *p* < 0.001 by Tukey's post‐hoc test. Error bars indicate ± standard deviation, N = 3 with technical triplicates.

Rheology was performed on hydrogels before and after culture with hMSCs and hUVECs to understand how changes in peptide degradation influenced the bulk mechanical properties of the hydrogels (**Figure** **6**). The storage moduli of the KLVAD‐LM peptide (71 ± 6 Pa) and PanMMP (69 ± 6 Pa) were similar for hydrogels formed in phosphate buffered saline (PBS) without cells (Figure 6A). Cells deposit a matrix during culture, and previous studies have shown that soft hydrogels can stiffen during culture.^[^ ^56^ ^]^ In our work, we found that hydrogels with hMSCs stiffen during culture, and that this was peptide‐dependent. Hydrogels with hUVECs or hMSCs stiffened from Days 0 to 3 for both PanMMP and KLVAD‐LM (*p* < 0.001 for comparisons). For both hUVECs and hMSCs KLVAD‐LM‐crosslinked hydrogels continued to stiffen throughout 14 days, while the PanMMP crosslinked hydrogels either had weaker mechanical properties (PanMMP, *p* < 0.01) or did not increase in stiffness (KLVAD‐LM). KLVAD‐LM hydrogels had an increase in storage modulus from 107 ± 13 Pa at Day 0 to 238 ± 28 Pa at Day 14 (*p* < 0.001), while PanMMP hydrogels only stiffened from 78 ± 11 Pa to 137 ± 28 Pa (*p* < 0.001). The difference between KLVAD‐LM and PanMMP at Day 14 was statistically significant (*p* < 0.001) (Figure 6B). Hydrogels with hUVECs also stiffened during culture, and this again was more pronounced in KLVAD‐LM gels, which had storage moduli increase from 89 ± 10 Pa to 260 ± 32 Pa (*p* < 0.001), while the storage moduli of PanMMP crosslinked hydrogels increased from 73 ± 7 Pa to 141 ± 22 Pa (*p* < 0.001) (Figure 6C). The difference in storage moduli between KLVAD‐LM and PanMMP hydrogels was not statistically significant at Day 0, but was at Day 14 (*p* < 0.001).

These results highlight the benefits of controlling matrix degradation during culture. Endothelial network formation is influenced by numerous factors, and the presence of other cell types has been shown to be beneficial by several research groups.^[^ ^21^, ^22^, ^23^, ^24^ ^]^ However, identifying the specific mechanisms by which other cell types promote network formation can be challenging as their presence modulates several variables, including both the mechanical and signaling environments. Hydrogels can be designed to tunably increase or decrease in stiffness during culture,^[^ ^57^, ^58^ ^]^ often through the use of photoactive molecules. The ability to tune matrix degradation such that the time‐dependent changes in mechanical properties can be controlled is a simple way that the effects of matrix properties can be controlled without needing additional cell types or chemistries.

To determine whether hydrogels crosslinked by the PanMMP and KLVAD‐LM‐crosslinked had different diffusivity values for macromolecules we performed fluorescence recovery after photobleaching (FRAP) (Figure S7, Supporting Information). To do this, we incorporated 40 kDa FITC‐dextran into the hydrogels and then photobleached a portion of the gel and quantified the diffusion of the 40 kDa dextran conjugate into the photobleached volume. We found that the PanMMP hydrogel had an average diffusivity of 38 ± 7.6 µm^2^ s^−1^, while the KLVAD‐LM‐crosslinked hydrogels had an average diffusivity of 50 ± 9.5 µm^2^ s^−1^, which is similar to literature values found for FITC‐dextran diffusion within PEG hydrogels.^[^ ^59^ ^]^

### 2.6 Validation of Peptide Degradation by MMP‐14

**Figure 7:**
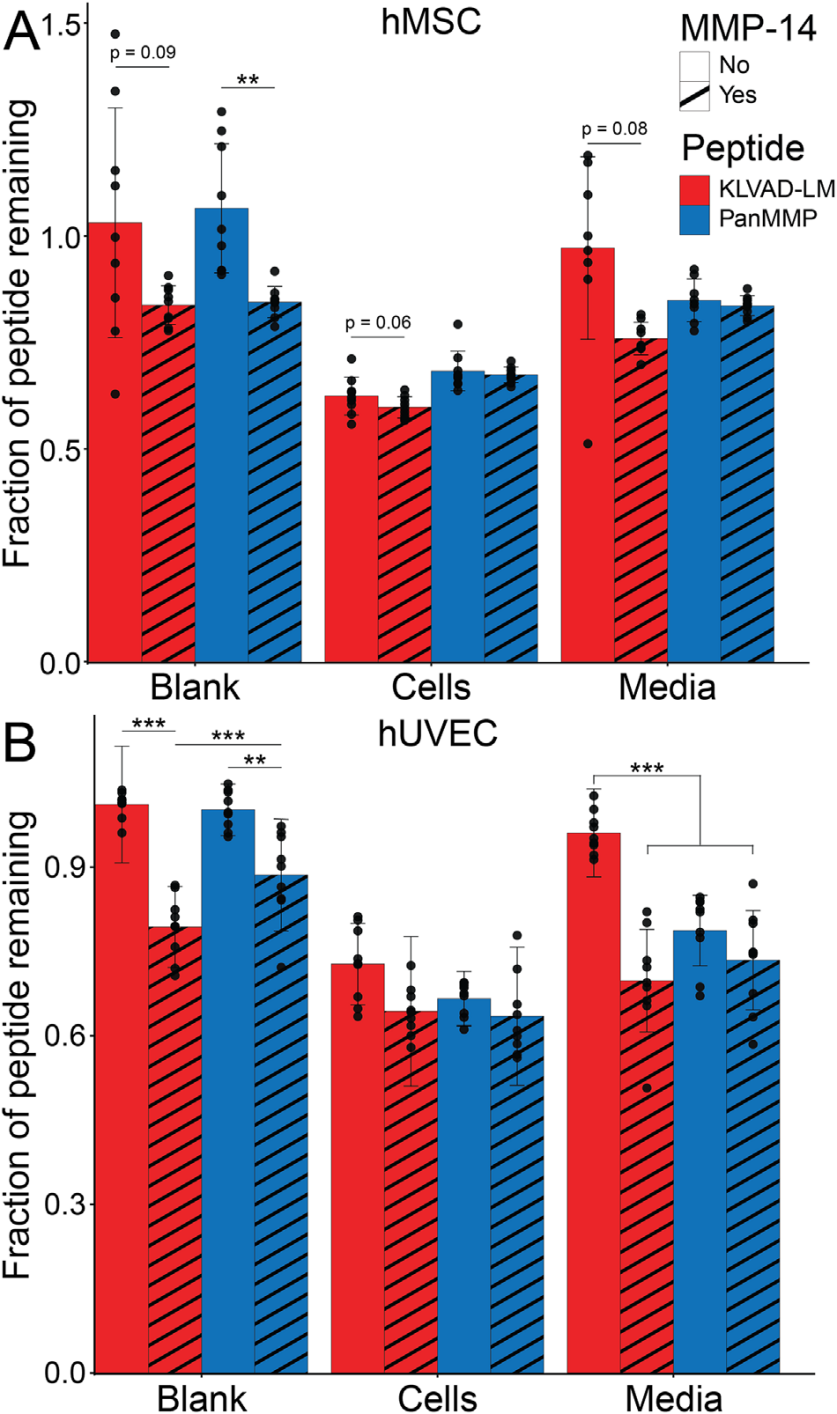
The KLVAD‐LM and PanMMP peptides were incubated for 24 h in cell culture media that did not come into contact with cells (Blank), cell culture media in the presence of cells (Cells), and cell‐conditioned media (Media). The extent of peptide degradation was quantified for samples both with and without the addition of exogenous MMP‐14. This was done for A) hMSCs and B) hUVECs. ^**^ indicates *p* < 0.01, and ^***^ indicates that *p* < 0.001 by Tukey's post‐hoc test. Error bars indicate ± standard deviation, N = 3 with technical triplicates.

To verify that MMP‐14 can degrade the KLVAD‐LM and PanMMP peptides, recombinant MMP‐14 was added to the different peptides, both in PBS and in the Cell and Media culture conditions (**Figure** **7**). Peptides were incubated under three conditions: in fresh cell culture media (“Blank”), in media with cells present (“Cells”), and in cell‐conditioned media (“Media”). This was done for both hMSCs and hUVECs, and was also done with and without the addition of exogenous MMP. Both PanMMP and KLVAD‐LM had negligible degradation in the Blank condition without added MMP‐14, indicating the stability of these peptides to hydrolytic degradation (Figure 7). Adding MMP‐14 to the Blank condition resulted in 10–20% degradation for the PanMMP and KLVAD‐LM peptides in both hMSC and hUVEC media, and this was statistically significant for all conditions except for the KLVAD‐LM peptide in hMSC media (*p* = 0.09).

Both peptides were between 60–68% of the initial peptide remaining after 24 h when cultured with hMSCs, and adding exogenous MMP‐14 had a minimal increase in degradation that was not statistically significant. A similar trend was seen in hUVECs, where between 65–72% of the peptides remained after 24 h of culture with cells, and adding MMP‐14 to the cell culture media increased degradation of either peptide by less than 3% when cultured with either cell type. The KLVAD‐LM peptide had low amounts of degradation when added to either hMSC or hUVEC conditioned media (97% and 93% remaining, respectively). Interestingly, adding exogenous MMP‐14 addition resulted in a minimal increase in the degradation of KLVAD‐LM in hUVEC‐conditioned media, reducing the peptide from 93 to 69% (*p* < 0.001).

Adding MMP‐14 to hMSC conditioned media reduced the amount remaining from 97% to 76%, although the difference was not statistically significant (*p* = 0.08). The PanMMP peptide was more degraded than the KLVAD‐LM peptide in media without exogenous MMP‐14 for hUVECs (78% remaining compared to 92% remaining, respectively, *p* < 0.001), however, unlike the KLVAD‐LM peptide, the PanMMP peptide showed minimal additional degradation in the media condition with added MMP‐14 for either hUVECs (78% remaining versus 72% with added MMP‐14), or hMSC (85% versus 84% with added MMP‐14).

These results highlight the benefits of utilizing crosslinking peptides that support cell spreading and viability while reducing time‐dependent changes to maintain hydrogel structure over extended periods of time. It also validates that incubating libraries of peptides with cells in culture is a promising way to rapidly screen hundreds of sequences, and that the degradation rate of soluble peptides is correlated with that of crosslinking peptides covalently bound to hydrogel matrices.

## 3 Conclusion

In conclusion, we have utilized an approach combining proteomics, functional degradation screens, and split‐and‐pool synthesis to identify and refine peptides to control cell‐matrix interactions within synthetic hydrogels. Through the use of a split‐and‐pool approach to generate larger peptide libraries, we identified a KLVAD‐LM‐ASAE peptide sequence that is less degraded by soluble proteases than the commonly used GPQGIWGQ “PanMMP” peptide, but still shows significant degradation in the presence of cells. Compared to the initially identified KLVAD‐NY‐ASAE peptide, the optimized KLVAD‐LM‐ASAE peptide had increased cell spreading and viability for hMSCs and hUVECs. The peptides identified in these screens had reduced degradation as soluble peptides. We incorporated fluorescent versions of these sequences into hydrogels to enable us to quantify peptide degradation during cell culture and found that the KLVAD‐LM peptide reduced degradation of the crosslinking peptide and PEG hydrogel on both the molecular and macroscopic level. Increased degradation of crosslinking peptides was also found to reduce the bulk rheological properties of hydrogels compared to less degraded KLVAD‐LM‐crosslinked hydrogels. Since protease activity is altered across a range of developmental, regenerative, and degenerative processes, this approach has the potential to improve our ability to harness cell‐matrix interactions for a therapeutic benefit.

## 4 Experimental Section

### Materials

All peptide synthesis reagents and amino acids were purchased from Ambeed or Chemscene. N,N‐Dimethylformamide (DMF) (VWR BDH Chemicals), dichloromethane (DCM) (VWR BDH Chemicals), piperidine (Millipore Sigma), trifluoroacetic acid (Millipore Sigma), and diethyl ether (Fisher Scientific) were used as purchased. 20 kDa 8‐arm poly(ethylene glycol) dibenzocyclooctyne (PEG‐DBCO) was purchased from Biopharm PEG. Batches of PEG were only used if they dissolved in water in under 30 s, and then they were used as received. Otherwise, the PEG was quickly purified by dissolving it in isopropanol (iPOH) and removing impurities using a 10 kDa Amicon centrifugal filter. Approximately 200 mg of PEG was dissolved in 20 mL of iPOH, and it was centrifuged at 4500 RPM until less than one milliliter of PEG‐DBCO/isopropanol was left in the filter. This was repeated 2×, and then the filtrate was dried via lyophilization and used.

### Methods–Peptide Synthesis Procedure

Peptides were synthesized using standard solid‐phase peptide synthesis (SPPS) protocols using an automated peptide synthesizer (CEM Liberty Blue) or manual synthesis. All SPPS used standard Fmoc‐protected amino acids on a Rink amide resin (Supra Sciences) unless otherwise noted. Amide couplings were done using O‐(6‐chlorobenzotriazol‐1‐yl)‐N,N,N′,N′‐tetramethyluronium hexafluorophosphate (HCTU), unless otherwise noted, or when needed for difficult couplings. For each coupling step the amino acid, HCTU, and DIPEA were added in turn in a 4:4:6 ratio to activate the amino acid, and then this solution was added to the synthesis resin. A ninhydrin test was performed after every coupling step to test for the presence of free primary amines. Upon a positive test, the coupling step was replicated until the test was negative. A capping step was then performed in which an acetic anhydride (Sigma‐Aldrich) solution (10:5:100 acetic anhydride:DIPEA:DMF) was added to the resin twice for 5 min, followed by 4× washing with DMF. A ninhydrin test was then performed to check for complete capping of the free amines. After successful amino acid coupling, the N‐terminal Fmoc group was removed by washing the resin with 20% piperidine in DMF twice for 5 min. A ninhydrin test was performed to check for a positive result, and then the next amino acid was coupled.

For split‐and‐pool steps the resin was washed 3× with DMF, and then the resin was weighed on a scale. This total mass of resin was divided by 22 and this amount was added into 19 separate tubes. Twenty‐two was used to account for resin loss during splitting, weighing, and transport. Coupling reactions were performed in 15 mL conical tubes, and coupling was verified using ninhydrin tests. Upon successful coupling of all 19 amino acids, the 19 fractions of resin were re‐combined and then split 19 ways again using the same protocol. To reduce the complexity of the individual libraries, these 19 fractions were combined into 10 different libraries.

All peptides containing tryptophan, including the peptide libraries, were cleaved using a cocktail containing 92.5% trifluoroacetic acid (TFA), 2.5% H_2_O, 2.5% triisopropylsilane (TIPS), and 2.5% dithiothreitol (DTT). DTT was not used for peptides that did not have tryptophan. Peptides were cleaved for 2 h at RT using 20 mL of cleavage solution per mM of peptide. Peptides containing azides were cleaved for 30 min to prevent degradation of the azide group. Peptide masses were verified using electrospray ionization (ESI), and peptides were re‐cleaved for 30 min if protecting groups remained attached. After cleavage, the peptides were precipitated in diethyl ether (DEE) and then centrifuged for 5 min at 4000 rpm, after which the supernatant was discarded. The peptide pellet was washed with DEE and centrifuged, and this was repeated 2×. The peptide pellet was dried, dissolved in water, and then neutralized with ammonium hydroxide prior to purification.

The cyclic RGD peptide was synthesized on a 2‐chlorotrityl chloride resin. The first amino acid was dissolved in DCM and added to the resin at 0.3 mmol gram^−1^ in a shaker vessel. Five equivalents of DIPEA were then added to the resin, and after 5 min on a wrist action shaker, another 1.5 equivalents of DIPEA were added to the resin. After 1 h, an excess of methanol was added to the resin for 30 min to quench any remaining reactive groups. Subsequent amino acids were then coupled using standard Fmoc‐synthesis protocols. Upon removal of the final Fmoc group, the resin was washed 3× in DMF and 3× in DCM. The peptide was then cleaved under mild acidic conditions consisting of 5% TFA and 2.5% TIPS in DCM. The cleavage solution was added to the resin for 5 min and then collected into a round‐bottom flask, and this was repeated until the resin turned dark red or black color. The peptide was then precipitated from the solution into DEE and pelleted using centrifugation. The protected peptide pellet was dissolved in 1:1 acetonitrile:water mixture, neutralized with 1M ammonium hydroxide, frozen, and lyophilized. The protected linear GRGDSK(N_3_) peptide was then cyclized in solution. This was done by dissolving the peptide into DMF at 1 mg mL^−1^ and adding 3 equivalents of (O‐(7‐azabenzotriazol‐1‐yl)‐N,N,N“,N”‐tetramethyluronium hexafluorophosphate) (HATU). After 6 h the reaction was checked using LC‐MS, and upon successful cyclization, the DMF was removed using rotary evaporation at 75 °C.

All peptides were purified using high‐performance liquid chromatography on a Phenomenex Gemini 5 µm NX‐C18 110 Å LC Column 150×21.2 mm. Elution gradients were run from 95% mobile phase A (water with 0.1% TFA) and 5% mobile phase B (acetonitrile with 0.1% TFA) to 100% mobile phase B. Each HPLC run began with a 2 min equilibration step, followed by a 10 min ramp from 95% mobile phase A to 100% mobile phase B, and followed by 2 min of equilibration at 100% Mobile Phase B, before ramping back down to the starting conditions. The split‐and‐pool libraries were purified by ramping up to 100% Mobile Phase B over 2 min. These libraries consisted of 19‐38 different peptides, and the purification process was not intended to separate the peptides from each other, but only to remove non‐peptide contaminants that do not interact strongly with the HPLC column. The protected cyclic RGD peptides were purified using an HPLC gradient that went from 30% mobile phase B to 100% mobile phase B. All peptides were lyophilized after purification and were then used, except for the protected cyclic RGD peptide, which was cleaved using 95% TFA, 2.5% TIPS, and 2.5% H_2_O for 1 h, followed by neutralization and lyophilization.

### General Cell Culture Procedure

Human mesenchymal stem cells (hMSCs) (Rooster Bio, Donor 310 305, a 20‐year‐old African American female) and human umbilical vein endothelial cells (hUVECs) (Lifeline Cell Technology, Donor 0 4608, an African American male) were cultured according to the manufacturers’ instructions using RoosterNourish hMSC media or Lifeline VascuLife EnGS hUVEC media.

### Peptide Degradation Study Procedure

hUVECs (75000 per well) or hMSCs (36000 per well) were seeded into a 48 well plate, and 500 µL media was added to the wells. The cells were cultured for 24 h, at which point the conditioned media was transferred into a new plate that does not contain cells, and 500 µL fresh media was added into the wells with cells. The peptide libraries were dissolved at a stock solution of 10 mM total peptide content. For libraries containing 38 peptides, 95 µL of the stock solution was added to each well; for libraries with 19 peptides, 47.5 µL was added to each well, and media was taken at 0 and 24 h. Add 4 µL of acetic acid to all collected media in the LC‐MS plates and store them in a −80 °C freezer to prevent further proteolytic degradation. From each sample, 10 µL of crude solution was introduced by the LC‐MS through a Thermo Scientific Vanquish LC System (Thermo Fisher Scientific), which outputted to a Thermo Scientific LTQ XL Linear Ion Trap Mass Spectrometer (Thermo Fisher Scientific). The sampled mixture was trapped on a column (ProntoSIL C18 AQ, 120 Å, 3 µm, 2.0 x 50 mm HPLC Column, PN 0502F184PS030, MAC‐MOD Analytical Inc.). The samples were loaded onto the column with a solvent containing acetonitrile/water, 5:95 (v/v) containing 1% acetic acid at a flow rate of 300 µL min^−1^ and held for 1 min. The sample was then eluted from the column with a linear gradient of 5–40% Solvent B (1% acetic acid in acetonitrile) at the same flow rate for 5 min. This was followed by a 1 min ramp up to 100% Solvent B, where it was re‐equilibrated with Solvent A (1% acetic acid) to 5% solvent B over the course of 1 min and held there for 2 min. The column temperature was kept at a constant 29 °C, and the mass spectrometer was operated in positive ion mode. Using a Heated ESI, the Source Voltage was set to 4.1 kV, and the capillary temperature was 350 °C. For non‐library degradation studies, 2.5 µL of 10 mM peptide stock solution for each peptide was added.

In the MMP‐14 inhibitor study, the MMP‐14 inhibitor NSC 405 020 (Cayman Chemical Company) was dissolved in DMSO to make a 10 mM stock solution and stored in −20 °C freezer. 0.5 µL MMP‐14 inhibitor stock solution was added to each well to achieve a 10 µM final concentration.

To assess the activity of the conditioned media, the media were divided into two equal portions. One portion was used for a standard degradation study, while the other was cultured for an additional 24 h before the PanMMP and KLVADLMASAE were added. After 24 h of incubation, the media were collected for further analysis. To assess the impact of recombinant MMP‐14 on PanMMP and KLVADLMASAE, recombinant MMP‐14 (antibodies.com) was dissolved in the media at a concentration of 0.5 nM. The remaining steps followed the standard degradation study protocol.

For all degradation studies using soluble peptides, a non‐proteolytically degradable NH_2_‐βFβAβAβAβAβAβA‐amide (βFβA6) peptide, where βF is β‐phenylalanine and βA is β‐alanine, was added at a 50 µM concentration and used as an internal standard. Peptide degradation ratios were measured by LC‐MS and analyzed by Xcalibur. The relative concentrations of the peptide were calculated by the ratio of the peak area of the peptide and the peak area of βFβA_6_.

### Enzyme‐Linked Immunesorbent Assay (ELISA)

250000 hUVECs or 125000 hMSCs were seeded into a 6 wells plate, and 10 mL media was added on top of the cells. After 24 h of culture, the cells were harvested by treatment with trypsin, followed by centrifugation at 1000 rpm for 5 min to pellet the cells. The cell pellets were then washed three times with phosphate‐buffered saline (PBS). The pellets were resuspended in prepared lysis buffer containing 1% Triton X‐100, 50 mM Tris‐HCl, 150 mM NaCl, 1 mM EDTA, and 1 mM phenylmethylsulfonyl fluoride (PMSF). The mixture was incubated on ice for 30 min with occasional vortexing to ensure complete cell lysis. The lysate was subsequently centrifuged at 10000 × g for 10 min at 4 °C to remove cell debris. The supernatant, containing the extracted proteins, was collected and stored the ELISA assay. Conditioned media collected from cell cultures were divided equally into two centrifuge tubes. One tube was immediately prepared for ELISA analysis, while the second tube was further centrifuged at 2,000*g* for 15 min and then at 10,000*g* for 30 min. The supernatant was then filtered through a 0.2 µm filter and transferred to polycarbonate tubes (Beckman Coulter, Fullerton, CA). The media was then ultracentrifuged at 100,000g for 70 min using a Type 50.2 Ti rotor (Beckman Coulter) in a LE‐80K ultracentrifuge (Beckman Coulter). The supernatant was collected and used for the ELISA assay. The concentration of MMP‐14 was quantitatively assessed using a commercially available ELISA kit (R&D Systems, Minneapolis, MN, catalog #DY918‐05), according to the manufacturer's instructions.

### Hydrogel Fabrication

8‐arm 20 kDa poly(ethylene glycol) (PEG) functionalized with dibenzocyclooctyne (DBCO) was purchased from BiopharmPEG and used as received. PEG batches that did not quickly go into solution were further purified. This was done by dissolving the 200 mg of PEG‐DBCO in 20 mLs of isopropanol and using a 10 kDa centrifugal filter to remove impurities by centrifuging the PEG‐DBCO at 4500 RPM until less than 1 mL was remaining. This was repeated two more times, and then the PEG‐DBCO left in the upper part of the filter was dried under vacuum and then used.

For all hydrogel studies, the total volume of hydrogel was 20 µL, made with 3.5% (wt/wt) PEG, 40% of the arms were functionalized with crosslinking peptides, and 10% were functionalized with cyclic‐RGD, with a final cell concentration of 20 million cells per mL. Briefly, the PEG was dissolved at 140 mg mL^−1^ in PBS, the crosslinking peptides were dissolved at 22.4 mM in PBS, and the cyclic RGD was dissolved at 5.6 mM in PBS. hMSCs were harvested from T‐75 flasks. After centrifugation and pouring the supernatant liquid, the cells were resuspended at 80 000 000 or 4 000 000 cells mL^−1^. The cyclic RGD and PEG PBS solutions were mixed at the ratio of 1:1. Add 10 µL of the PEG‐cyclic RGD mixer in the wells and incubate for 10 mins. The crosslinking peptides and cell solutions were mixed at a ratio of 1:1. 10 µL of the peptide‐cell mixture was pipetted into the PEG‐cyclic RGD mixer previously dropped in the wells. The solutions were pipetted until well mixed, and after 15 min in the incubator 1 mL of media was added into each well and kept in the incubator. For gels intended for imaging, 12 mm round glass coverslips were placed in each well of the 24‐well plates prior to hydrogel fabrication. For cell viability test, sylgard layers were prepared in 24 well plates to prevent unwanted cell growth on the bottom of the well wall. Sylgard kit Part A/Part B was mixed in a 10:1 ratio by volume, and the mixture was added to the 24 well plates such that the bottom of each well was completely covered by Sylgard. The plates were placed in a biosafety hood overnight with UV light on. For all studies, three technical replicates were done per condition, and the studies were repeated three times.

### Phalloidin/DAPI Staining

The hydrogels were washed with PBS three times and incubated in 4% formaldehyde in PBS for 20 min. The hydrogels were again washed with PBS three times, followed by an incubation in 0.25% Triton X‐100 in PBS solution for 20 min. The hydrogels were washed with PBS three times, followed by a 20‐min incubation of in 1% BSA in PBS. The gels were washed 3x in PBS, and 700 µL of Phalloidin/DAPI PBS staining solution (0.1% BSA, 1 µg mL^−1^ DAPI, and 1 µg mL^−1^ phalloidin‐iFluor 555) was added to each well, and it was incubated for 1 h in the dark at room temperature. The hydrogels were washed 3x, and 1 mL PBS was added to each well, and they were stored in a 4 °C until they were ready for imaging.

### Quantification of Cell Spreading

Hydrogels were imaged by confocal microscope (LSM 800, AxioObserver, EC Olan‐Neofluar 10x/0.3 objective). The 12 mm round slides with hydrogels were taken out of 24 well plates and flipped gel side down on round‐bottom slides. Confocal microscopy was carried out in the Z‐stack mode. Phalloidin was excited at 488 nm and recorded between 493 and 630 nm. DAPI was excited at 405 nm and recorded between 410 and 501 nm. Cells in each hydrogel were measured with ImageJ software according to the particle analysis results and then averaged to obtain the mean cell area in the hydrogels.

### Viability Testing–AlamarBlue Metabolic Activity Assay

On Days 1, 3, and 7, the media from the wells was removed and 500 µL of a 10× diluted Alamar Blue stock solution in media was added to each well with hydrogels. Three wells without any cells were used for background subtraction. The gels were incubated for 4 h, at which time the absorbance was measured with a spectrophotometer at a wavelength of 579 nm (600 nm reference). The cell viability percentage was assessed according to the manufacturer's protocol.

### DNA Quantification Assay

Hydrogels seeded in Sylgard‐coated plates were homogenized in 300 mL Papain digestion solution (125 µg mL^−1^ Papain, 2 mM L‐cysteine, and 0.333 M EDTA) overnight at 60 °C to dissolve the samples and completely release their Double Stranded DNA (dsDNA) into solution. dsDNA was quantified using the Quantiflour dsDNA System (E2670, Promega) according to the manufacturer's protocol. A plate reader (SpectraMax iD3, Molecular Devices) was used to measure the fluorescence (504 nm Ex /531 nm Em).

### Quantification of Peptide and Hydrogel Degradation

The peptides Cy3‐KKGPQGIWGQKKK(N_3_) and Cy3‐KKKLVADLMASAEKK(N_3_) were synthesized by conjugating Cy3 to the N‐terminus and Lys(N_3_) to the C‐terminus of KKGPQGIWGQKK and KKGPQGIWGQKK peptides, respectively. Two additional lysine residues were introduced at both terminal ends to balance the hydrophobicity of the Cy3 label and to ensure the solubility of the peptides in the media. FAM‐K(N_3_)‐ βA_2_KβA_2_KβA_2_ peptides were synthesized by adding an N‐terminal Lys(N_3_) to βA_2_KβA_2_KβA_2_ and functionalizing the N‐terminal amine with 5(6)‐carboxyfluorescein. The total volume of hydrogel was 20 µL, made with 3.5% (wt/wt) PEG, 40% of the arms were functionalized with crosslinking peptides, 10% were functionalized with cyclic‐RGD, 1% were functionalized by FAM‐K(N_3_)‐βA_2_KβA_2_KβA_2_ and 1% were functionalized by Cy3‐labeled peptides, with a final cell concentration of 20 million cells per mL. In detail, the Cy3‐labeled peptides and FAM‐K(N_3_)‐ βA_2_KβA_2_KβA_2_ peptides were dissolved in PBS at a concentration of 3.5 mM in preparation for hydrogel fabrication. PEG was dissolved in PBS at a concentration of 175 mg mL^−1^ to maintain a consistent hydrogel volume. The PEG, cyclic RGD, FAM‐labeled peptides, and Cy3‐labeled peptides solution was mixed in a 4:5:0.5:0.5 ratio. Hydrogels were then prepared following the standard hydrogel fabrication procedure. Hydrogels were collected on days 0, 3, 7, and 14, washed three times with PBS to remove unbound fluorescence from peptide degradation, and stored at −80 °C for further analysis.

Fluorescent hydrogels were digested in 300 µL trypsin digestion solution (0.5 mg mL^−1^ trypsin in 50mM ammonium bicarbonate buffer) for 4 h at 37 °C to release the covalently attached Cy3 and FAM fluorophores into the solution. A plate reader (Tecan Infinite 200Pro) was used to measure fluorescence (526nm Ex /572nm Em for Cy3 and 470nm Ex /518nm Em for FAM).

### Rheological Characterization of Hydrogels During Culture

20 µL hydrogels with 20 million cells ml^−1^ hMSCs or hUVECs were fabricated according to the standard hydrogel fabrication protocol previously introduced and then cultured for 0, 3, 7, and 14 days. At each time point, the stiffness of the hydrogels was measured using an AR‐Ex 2000 rheometer equipped with an 8 mm parallel plate geometry. To perform the measurements, 12 mm diameter hydrogel‐loaded slides were removed from 24‐well plates and carefully placed on the rheometer stage, ensuring that the hydrogels were in direct contact with the middle of the geometry. The parallel plate geometry was then manually lowered until the hydrogel fully covered the surface of the geometry. A frequency sweep was performed at a constant strain of 1% over a frequency range of 0.1 to 10 Hz. The storage modulus at 1 Hz frequency from the frequency sweep was used for quantification.

### Fluorescence Recovery After Photobleaching (FRAP)

Hydrogels crosslinked by N_3‐_KKGPQGIWGQKK(N_3_) and N_3_‐KKKLVADLMASAEKK(N_3_) were incubated overnight in PBS containing 100 mg mL^−1^ 40 kDa FITC‐dextran. The 12 mm diameter hydrogel‐loaded slides were removed from the 24‐well plates and inverted with the gel side facing downward onto round‐bottom slides. Hydrogels were imaged by confocal microscope (LSM 800, AxioObserver, EC Olan‐Neofluar 10x/0.3 objective). A circular spot area (50 *µ*m dia.) was monitored by 5 pre‐bleach image scans (500 ms interval) at a 3% power 488 nm laser and photobleached for 340 ms at 100% laser power, with recovery monitored for up to 70 s.

For quantitative analysis, the normalized intensity (*I_N_* (*t*)) was calculated using the following equation:

(1) $$I_{N}\left(t\right)=\frac{I\left(t\right)-I_{0}}{I_{f}-I_{0}}$$

where *I*_0_ represents the initial intensity after photobleaching, and *I_f_* represents the final intensity after recovery has fully occurred. The recovery model (*R*(*t*)) was fitted to the following equation:

(2) $$R\left(t\right)=I_{f}-\left(I_{f}-I_{0}\right)*e^{-t/\tau_{D}}$$

where τ_*D*_ is the time constant related to the diffusion coefficient. The diffusion coefficient (*D*) is given by:

(3) $$D=\frac{r^{2}}{4\tau_{D}}$$

### Statistical Analysis

Statistical analysis was done using a Student's *t*‐test for comparisons with two conditions, and ANOVAs with a Tukey post‐hoc test for comparisons with more than two conditions. For peptide degradation studies, the amount of peptide in each sample was initially normalized to the βFβA6 internal standard, and then 24 h time points were divided by the 0 h time points to calculate fraction of peptide remaining. Samples were determined to have undergone statistically significant degradation when a paired t‐test between the Time Zero time point and later time point was statistically significant. All statistical analysis was done in R, and samples were considered statistically significant if they had a p‐value of less than 0.05.

## Conflict of Interest

The authors declare no conflict of interest.

## Supporting information



Supporting Information

## Data Availability

The data that support the findings of this study are available from the corresponding author upon reasonable request.
