# Supplementary material for: Using High‐Throughput Screening to Identify Crosslinking Peptides That Control Cell‐Mediated Matrix Degradation
Source: Adv Healthc Mater. 2025 Aug 25;14(31):e01932. doi: 10.1002/adhm.202501932 (PMC12683216; doi:10.1002/adhm.202501932)
Supplement: Supplementary file 1 — Supporting Information [file ADHM-14-0-s001.pdf]

ADVANCED HEALTHCARE MATERIALS

Supporting Information

for *Adv. Healthcare Mater.*, DOI 10.1002/adhm.202501932

Using High-Throughput Screening to Identify Crosslinking Peptides That Control Cell-Mediated Matrix Degradation

*Yingjie Wu, Samuel J. Rozans, Abolfazl Salehi Moghaddam and E. Thomas Pashuck**

Using High-Throughput Screening to Identify Crosslinking Peptides that Control Cell-Mediated Matrix Degradation

Yingjie Wu, Samuel J. Rozans, Abolfazl Salehi Moghaddam, and E. Thomas Pashuck

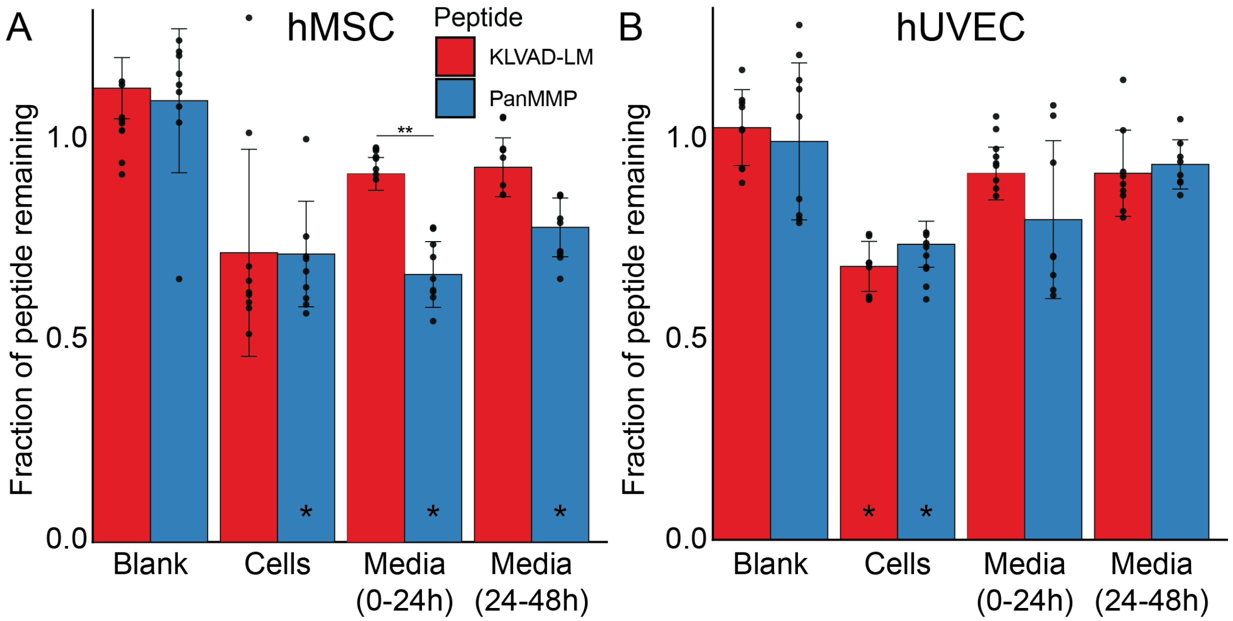

Figure S1. We modified our degradation procedure to better understand time-dependent changes in the activity of soluble proteases in the Media condition. We cultured cells for 24 hours and the put the conditioned media into two different wells. In the first well we immediately added the KLVAD-LM and PanMMP peptides and incubated them for 24 hours as the Media (0-24h) condition. In the second well we added the peptides after 24 hours, and then performed a degradation study for 24 hour (hours 24-48). The KLVAD-LM was less than 10% degraded across all Media conditions, while the PanMMP peptides had statistically significant degradation in media conditions for both (**A**) hMSCs and (**B**) hUVECs. Bars marked with an asterisk had statistically significant degradation as determined by paired t-test comparing the initial values for a well with the values after 24 hours of culture. Comparison between peptides was done using a t-test. * indicated $p < 0.05$, ** indicates $p < 0.01$. Error bars represent ± standard deviation. N = 3 with technical triplicates.

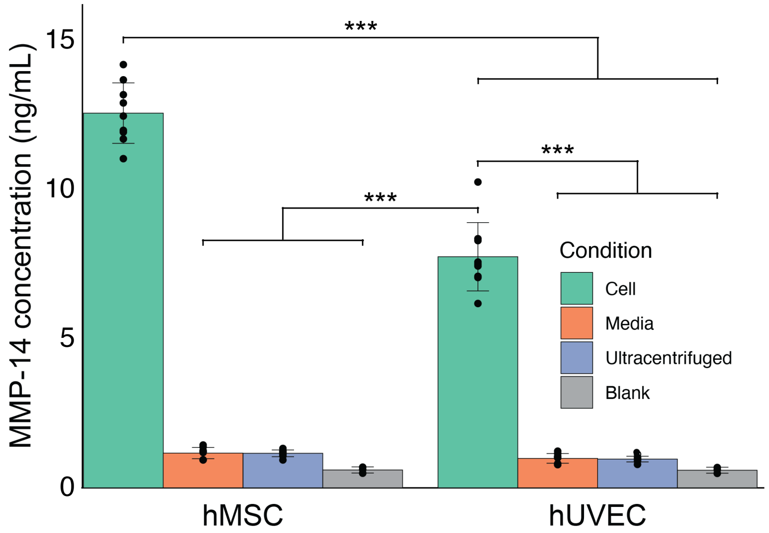

Figure S2. We performed an ELISA on MMP-14 for both hMSCs and hUVECs. Cells were cultured in media for 24 hours and then the concentration of MMP-14 was quantified the in the cells (Cell) and in the conditioned media (Media). MMP-14 can be present on extracellular vesicles (EVs), and the conditioned media was ultracentrifuged to deplete the EVs, and the MMP-14 concentration EV-depleted media was also quantified (Ultracentrifuged condition). We compared this to the 0 ng/mL data point from the standard curve (Blank). *** indicates that $p < 0.001$ by Tukey's post-hoc test. Error bars represent ± standard deviation. N = 3 with technical triplicates.

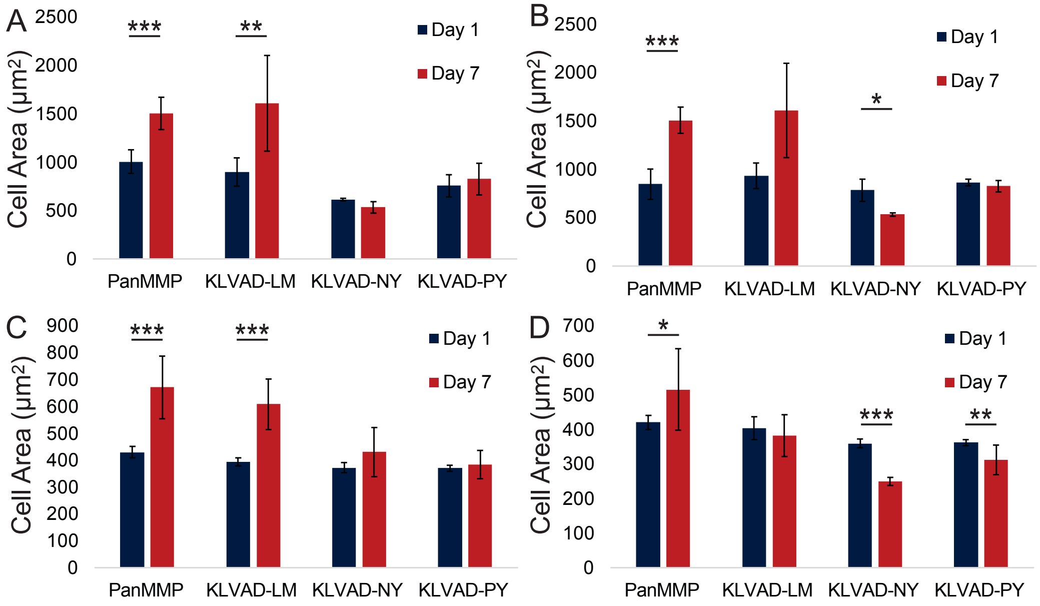

Figure S3. Cells were cultured in PEG hydrogels at 1 million cells/mL with and without an MMP-14 inhibitor and their area was measured using confocal microscopy. (**A**) hMSCs without the inhibitor, (**B**) hMSCs with the inhibitor (**C**) hUVECs without the inhibitor and (**D**) hUVECs with the inhibitor. Notably, the ability of hUVECs to spread in KLVAD-LM gels after 7 days was diminished when the MMP-14 inhibitor was added. * indicates $p < 0.05$, ** indicates $p < 0.01$, and *** indicates that $p < 0.001$ by Tukey's host-hoc test. Error bars represent ± standard deviation, N = 3 with technical triplicates.

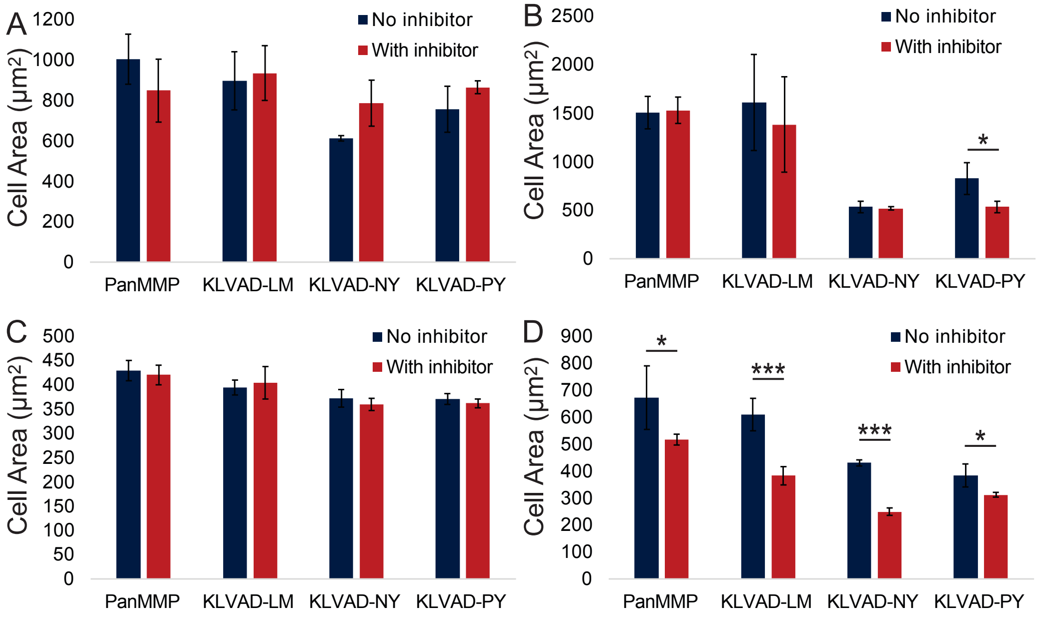

Figure S4. Cells were cultured in PEG hydrogels 1 million cells/mL with and without an MMP-14 inhibitor and their area was measured using confocal microscopy. (**A**) hMSCs at Day 1, (**B**) hMSCs at Day 7, (**C**) hUVECs at Day 1, and (**D**) hUVECs at Day 7. * indicates $p < 0.05$, ** indicates $p < 0.01$, and *** indicates that $p < 0.001$ by Tukey's host-hoc test. Error bars represent ± standard deviation, N = 3 with technical triplicates.

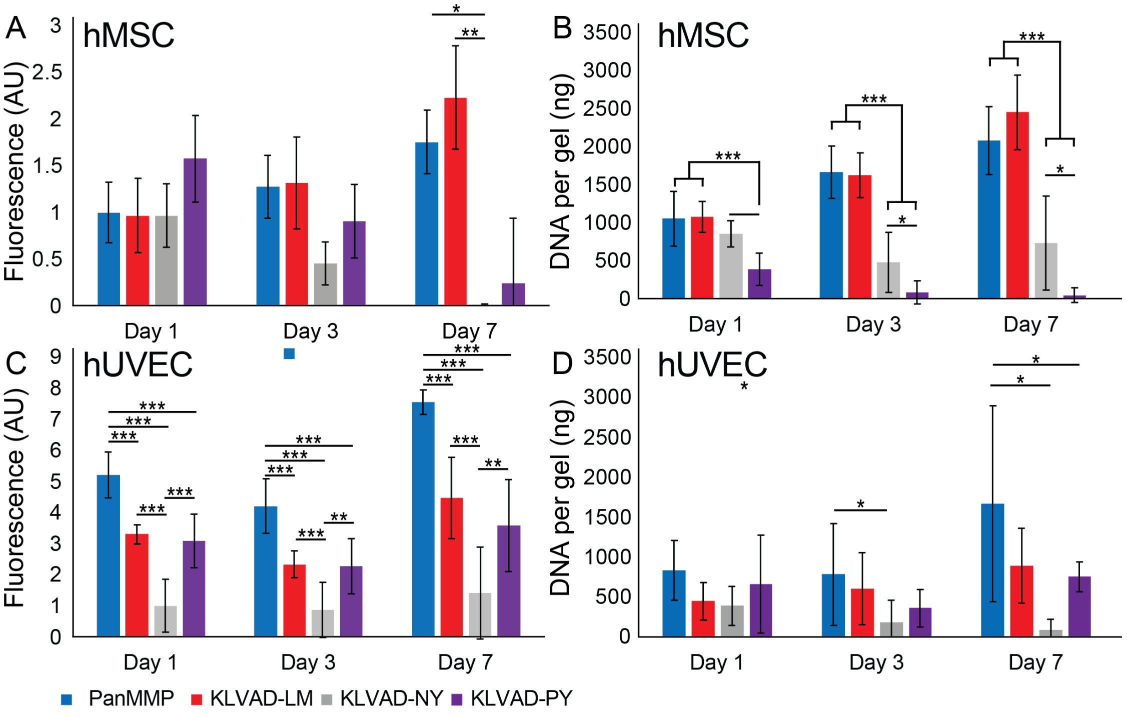

Figure S5. The sequence of crosslinking peptides influenced cell viability within hydrogels. The Pan-MMP and KLVAD-LM peptides had higher hMSCs viability as determined by both (**A**) alamarBlue viability and (**B**) DNA quantification, while the viability for hUVECs had less dependence on peptide for both alamarBlue (**C**) and DNA quantification (**D**). * indicates $p < 0.05$, ** indicates $p < 0.01$, and *** indicates that $p < 0.001$ by Tukey's host-hoc test. Error bars represent ± standard deviation, N = 3 with technical triplicates.

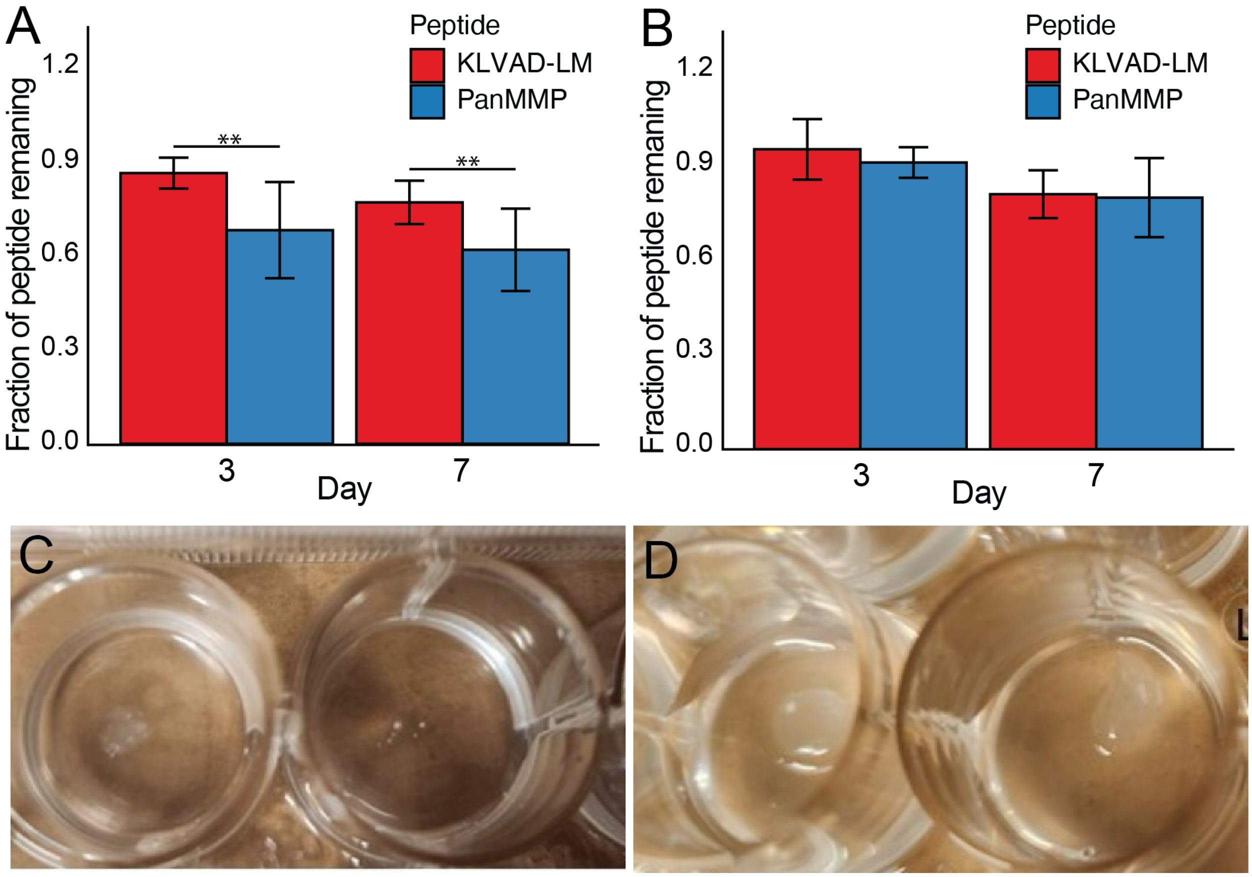

Figure S6. Cells were cultured in PEG hydrogels 1 million cells/mL the amount of peptide degradation was quantified at Day 3 and 7 (**A**) hMSCs and (**B**) hUVECs. Hydrogels with 20 million hMSCs per mL were cultured for seven days. (**C**) Hydrogels crosslinked by the PanMMP peptide showed macroscopic degradation, while (**D**) those crosslinked by the KLVAD-LM peptide were still visibly intact. ** indicates $p < 0.01$ by a Student's t-test. Error bars represent ± standard deviation, N = 3 with technical triplicates.

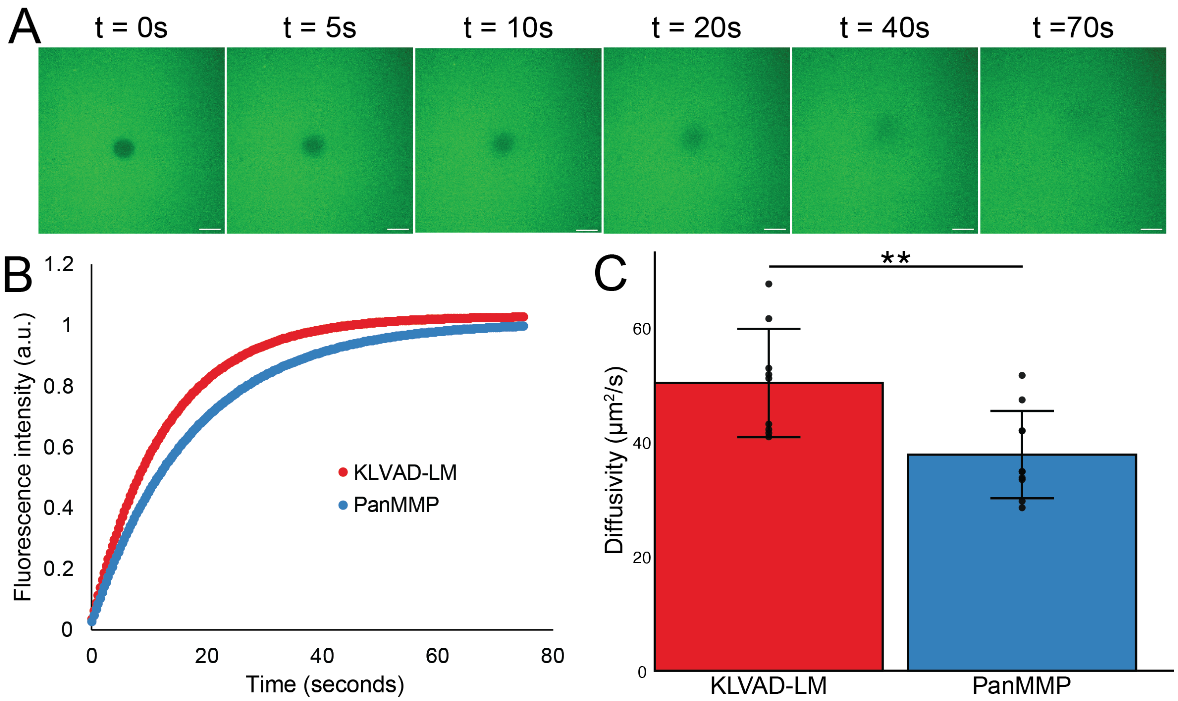

Figure S7. Fluorescence recovery after photobleaching (FRAP) was performed to quantify the diffusion of 40 kDa FITC-dextran within the hydrogel matrices. (**A**) Visualization of diffusion of 40 kDa FITC-dextran into the photo bleached area of a PanMMP hydrogel. (**B**) Quantification of fluorescence intensity returning to the photo bleached area for both KLVAD-LM and PanMMP hydrogels. (**C**) Quantification of diffusivity within KLVAD-LM and PanMMP crosslinked hydrogel . ** indicates $p < 0.01$ by a Student's t-test. Error bars represent ± standard deviation, N = 3 with technical triplicates.

TAILS Proteomics Procedure

Cells were grown in their respective media until they reached 70% confluence, and four T-75 flasks were used per cell type. The media was removed and the cells were washed 3x to remove serum proteins and dead cells. Serum-free media was added for 30 minutes, and then replaced with fresh serum-free media. Cells were grown for 24 hours at which time the media was collected, treated with the HALT protease inhibitor cocktail, and centrifuged at 2,200g to remove dead cells. The centrifuged media was filtered using a 0.2 µm syringe filter and 15% (v/v) of 100% trichloroacetic acid (TCA) was added to precipitate the proteins. This was put on ice for 3-4 hours, and then centrifuged at 9000g for 15min at 4°C and the supernatant was decanted. The pellet was washed with freezer-cold (-20 °C) acetone and it was centrifuged again at 9000g for 15min at 4 °C. Three acetone washes per performed and then the pellet was resuspended at 8M guanidinium hydrochloride was added to make a protein concentration of 2 mg/mL, which was determined using a BCA protein quantification assay.

Disulfide reduction an alkylation

The pellet was sonicated until it went into solution, and then this was mixed 1:1 with 200 mM HEPES at pH 7 to make a final protein concentration of ~1-2 mg/mL. A fresh solution of 1M dithiothreitol (DTT) was made and added to make a final DTT concentration in the sample of 5 mM. This was incubated with the proteins for 1 hour at 65 °C to reduce the disulfide bonds on the proteins. The samples were cooled to room temperature, and then a solution of 0.1M iodoacetamide was added to a final concentration of 15 mM to alkylate the cysteines on the proteins. The sample was vortexed and incubated in the dark for 30 minutes at room temperature. 20 µL of 1M DTT was added per mL and vortexed.

The proteins were then isolated using a water:methanol:chloroform protocol. 3 mLs of ice-cold water were added per mL of protein solution. This was then mixed with 4 mLs of ice-cold methanol per mL of protein solution. Then 1 mL of ice-cold chloroform was added per mL of protein solution, and it was vortexed to mix the solution. This was centrifuged at 3,000 x g at 4 °C for 7 min and the white protein interphase was isolated using a spatula and transferred to a 1.5 mL microcentrifuge tube. This pellet was washed 3x with 1 mL of methanol.

Dimethylation of primary amines
The pellet was resuspended/dissolved in 100 µL of 0.1 M NaOH was added to the pellet, followed by 50 µL of 1M HEPES, pH 7.5, and 100 µL of water. The pellet was sonicated until the protein went into solution. This was mixed with 250 µL of 6 M guanidinium hydrochloride and the pH was adjusted to 7.5 using either NaOH or HCl. A fresh 2M working stock of formaldehyde and a fresh 1M working stock of $NaBH_3CN$ (typically 6.3 mg/100 µL of water) were made. 10 µL of 2M formaldehyde was added to the 500 µL protein solution make a 40 mM final concentration, immediately followed by 10 µL of of the 1M $NaBH_3CN$. The pH was adjusted to 6-7 and this was incubated overnight at 37°C to dimethylate all primary amines on the proteins, including both lysines and N-terminal amines. The following morning an additional 20 mM of fresh formaldehyde and 10 mM of fresh $NaBH_3CN$ was added and incubated at 37°C for one hour to ensure complete dimethylation and the reaction was quenched by adding 1 M Tris, pH 6.8 to a 100 mM final concentration. The samples were precipitated using the water:methanol:chloroform protocol to precipitate the proteins and the pellet was washed with methanol 3x.

Trypsin digestion of proteins
The pellet was resuspended in ~100 µL of 6 M GuCl and then diluted ten-fold with 100 mM HEPES, pH 8.0 for trypsin digestion. Proteomics-grade trypsin was added to the solution at a 1:100 trypsin:sample ratio and mixed with pipetting and vortexing. The sample was incubated overnight at 37°C.

Negative selection of dimethylated peptides using HPG-ALD polymer
The HPG-ALD polymer was used to remove tryptic peptides. N-terminal amines present in the sample during the dimethylation step, such as those which were generated from proteolytic cleavage of the protein, were dimethylated and will not be reactive towards the aldehyde groups in the high molecular weight HPG-ALD polymer. N-terminal amines generated in the trypsinization step will react with the aldehyde moieties on the high molecular weight HPG-ALD polymers and can be separated from the peptides with dimethylated N-termini. The frozen HPG-ALD polymer solution was thawed at room temperature and added at a polymer:peptide ratio of 5:1 and then immediately after freshly prepared $NaBH_3CN$ was added to a 20 mM final concentration and the pH was adjusted to 6-7 using either NaOH or HCl. The samples were then incubated overnight at 37°C.

N-terminome sample recovery
1M Tris buffer pH 6.8 was added to a 100 mM final concentration. The pH was checked to be between 6-7 and it was incubated at 37°C to quench the aldehydes on the HPG-ALD polymer. An 10-kDa MWCO 0.5 mL Amicon centrifugal filter was pre-washed with 400 µL 100 mM NaOH, centrifuged at 12,000 rcf for 5 min and the flow-through was discarded. A second wash with deionized water was done at 12,000 rcf for 5 min and the flow-through was again discarded. 300 µL of the HPG-ALD/peptide mixture was laded into the centrifugal filter and it was centrifuged at 12,000 rcf for 10 minutes and the flow through which contains the desired dimethylated peptides was collected and transferred to a fresh 15 mL tube. The remaining HPG-ALD/peptide mixture was added to the centrifugal filter and was spun at 12,000 rcf for 10 minutes and the flow through was collected. The remaining HPG-ALD in the filter was washed with 50:50 methanol:water to solubilize hydrophobic peptides and the centrifugation step was

repeated and the flow through was collected into the 15 mL tube. The combined flow throughs were lyophilized, and then re-dissolved in 1-2 mL of water with 5% acetonitrile and 0.1% trifluoroacetic acid. This was then injected onto a prep-HPLC for desalting. Briefly, after the sample was injected the gradient was kept at 95% water and 5% acetonitrile with 0.1% TFA for five minutes to allow the salts to elute. The gradient was then ramped up to 100% acetonitrile (with 0.1% TFA) over two minutes and held there for three minutes, at which point it was ramped back down to 95% water and 5% acetonitrile with 0.1% TFA. Fractions which eluted peptides after the beginning of the ramp were collected, combined, and lyophilized.

Cation exchange chromatography
Dimethylated peptides were then fractionated using strong cation exchange (SCX) chromatography using a 100 mm × 4.6 mm, 5 µm, 300 Å PolySULFOETHYL SCX column from PolyLC. One mg was loaded onto the column per injection, and a gradient from 100% Buffer A (30% acetonitrile, 5 mM KH_2PO_4, pH 2.7) to 100% Buffer B (30% acetonitrile, 5 mM KH2PO4, 0.5 M NaCl, pH 2.7) over 30 minutes and was run at a flow rate of 1 mL per minute. The collected fractions were combined into approximately 10 fractions and lyophilized. The samples were then dissolved in 1 mL of water, and desalted using C18 Sep-Pak Vac 6cc cartridges. Briefly the columns were conditioned with 6 ml of isopropanol and equilibrated with 6 ml of 0.1% formic acid. Once the 0.1% formic acid had been flushed through, the samples were loaded and it was washed with water containing 4.25% acetonitrile and 0.1% formic acid to wash the salts through. The peptides were then eluted with 50:50 acetonitrile:water and 0.1% formic acid. The samples were then lyophilized and ready for liquid chromatography mass spectrometry.

Proteomics Using Liquid Chromatography Mass Spectrometry
Samples were either sent to the proteomics core facility at the University of Pennsylvania or analyzing in the lab using a Thermo Fisher Vanquish ultra high pressure liquid chromatography (UPLC) and an LTQ-XL mass spectrometer using a HALO Peptide ES-C18 1.0x250mm 2.7 µm UHPLC Column 1.0x250mm (Mac-Mod) at 55°C under micro-flow 50 µL/min conditions.(1) The samples were dissolved into 40 µL and 10 µL was injected onto the UPLC column. The mobile phase was ramped between 95% Phase A (water with 1% acetic acid) and 5% Phase B (acetonitrile with 1% acetic acid) over 60 minutes and peptides were identified using a Top 5 MS/MS method.

Proteomics Analysis
The data was processed using the trans-proteomics pipeline (TPP) (http://www.tppms.org/). The LTQ RAW data was converted to mzXML format in profile mode (not centroid) using msconvert tools in the TPP. Peptides were identified using both the X!Tandem and Mascot databases and peptide validation was done using the Peptide Prophet. For X!Tandem searches the following parameters were used: fragment mass tolerance 0.8 kDa and fixed modifications including: cysteine carbamidomethylation (+57.021464) and peptide N-terminal and lysine dimethylation (+28.031300). Variable modifications: methionine oxidation (+15.994915), asparagine deamidation (+0.984016) and glutamine deamidation (+0.984016) For Mascot searches the following peptide modifications were used: cysteine carbamidomethylation (+57.021464), peptide N-terminal and lysine dimethylation (+28.031300); variable modifications: methionine oxidation (+15.994915), asparagine deamidation (+0.984016) and glutamine deamidation (+0.984016).

The results from the X!Tandem and Mascot searches were analyzed using the Xinteract, PeptideProphet and XPRESS tools of the TPP. The following PeptideProphet options were used: ‘Use accurate mass binning’, ‘Do not use the NTT model’ and ‘Use decoy hits to pin down the negative distribution’ and to provide the correct identifier for decoy proteins. The output of was an interact pepXML file that included all the peptides and their related information including the peptide sequence, protein, and scoring.

Peptides which had greater than 95% likelihood of an accurate match were selected and peptides with a cysteine were removed. Peptides in this list which came from proteins found either on the cell membrane or extracellularly were then selected. The pepXML file gives the sequence of the peptides found in the fragment identified in LCMS. Since this only the C-terminal side of the site of proteolytic cleavage, the Uniprot database (https://www.uniprot.org/) was used to identify the amino acids on the N-terminal side of the cleavage. Typically peptides of approximately ten amino acids were used, roughly five amino acids on either side of the cleavage site, however this was occasionally shifted to include more hydrophilic/charged amino acids within the sequence to help ensure that the peptides are soluble in cell culture media.

Cyanine Dye Synthesis

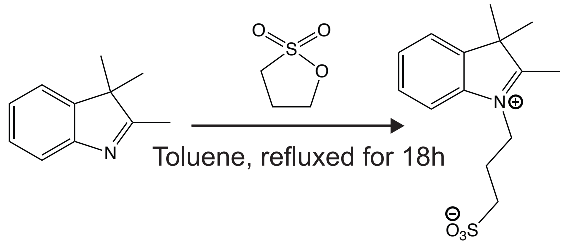

Synthesis of sulfonated indolenine[(2)]*:* A mixture of 2,3,3-trimethylindolenine (20.0 mL, 127.0 mmol) and 1,3-propanesultone (15.5 g, 127.0 mmol) was refluxed in toluene (500 mL) for 18 h, which resulted in the formation of a dark red precipitate. The reaction was then cooled to room temperature and was concentrated under reduced pressure. The product was then dissolved in dichloromethane and then precipitated in ethyl ether in 50 mL centrifugal tubes. These were then centrifuged to concentrate the product and the ether was decanted. The product was dried under reduced pressure via lyophilization to yield a red crystalline powder.

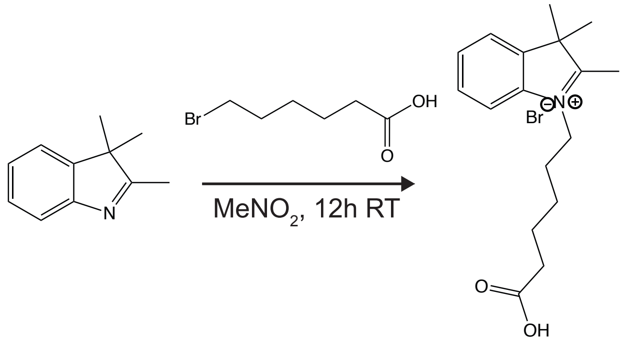

Synthesis of carboxylated indolenine: 6-bromohexanoic acid (1.0 mol) was added to a solution of the 2,3,3-trimethylindolenine (1.0 mol) in nitromethane ($MeNO_2$) (600 mL). The reaction was initially cooled on ice, then stirred for 24 hours at room temperature. The reaction mixture was then triturated with diethyl ether in 50 mL tubes, centrifuged to concentrate the product, and the ether was decanted. The product was then dried under vacuum on a lyophilizer. [(3)]

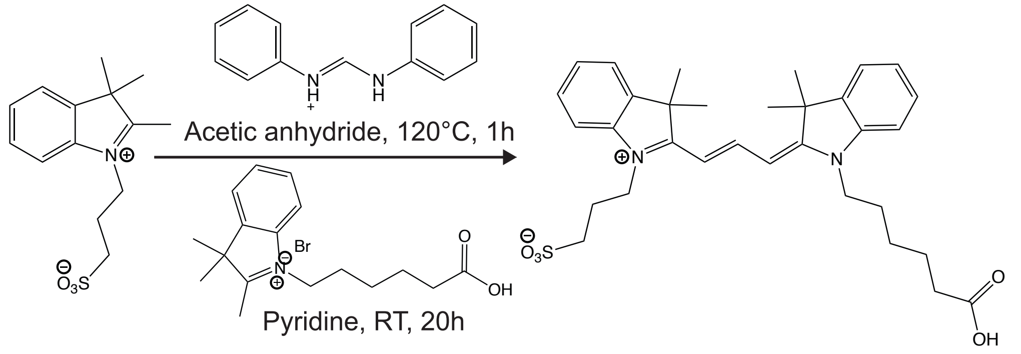

Synthesis of Cy3: The sulfated indolenine (50.0 mmol) and *N*,*N*-diphenylformamidine (60.0 mmol) were dissolved in acetic anhydride (150 mL) and heated at 120 °C for 30 min in an oil bath. The reaction was then cooled to room temperature and a solution of the carboxylated indolenine (70 mmol) dissolved in dry pyridine (150 mL) was added. The mixture was stirred at room temperature for 12 h and concentrated. The residue was then dissolved in chloroform (100 mL) and precipitated in diethyl ether (1 L). The residue was purified using silica gel chromatography using methanol in dichloromethane as eluent (gradient of 10-20%).

References

1. Lenčo J, Vajrychová M, Pimková Kn, Prokšová Mn, Benková Mt, Klimentová J, Tambor Vc, Soukup Oe. Conventional-flow liquid chromatography–mass spectrometry for exploratory bottom-up proteomic analyses. Analytical chemistry. 2018;90(8):5381-9.
2. Rose NC, Sanchez AV, Tipple EF, Lynam JM, Spicer CD. Insight into ortho-boronoaldehyde conjugation via a FRET-based reporter assay. Chemical Science. 2022;13(43):12791-8.
3. Kvach MV, Ustinov AV, Stepanova IA, Malakhov AD, Skorobogatyi MV, Shmanai VV, Korshun VA. A convenient synthesis of cyanine dyes: Reagents for the labeling of biomolecules. Wiley Online Library; 2008.

Peptide Characterization

A

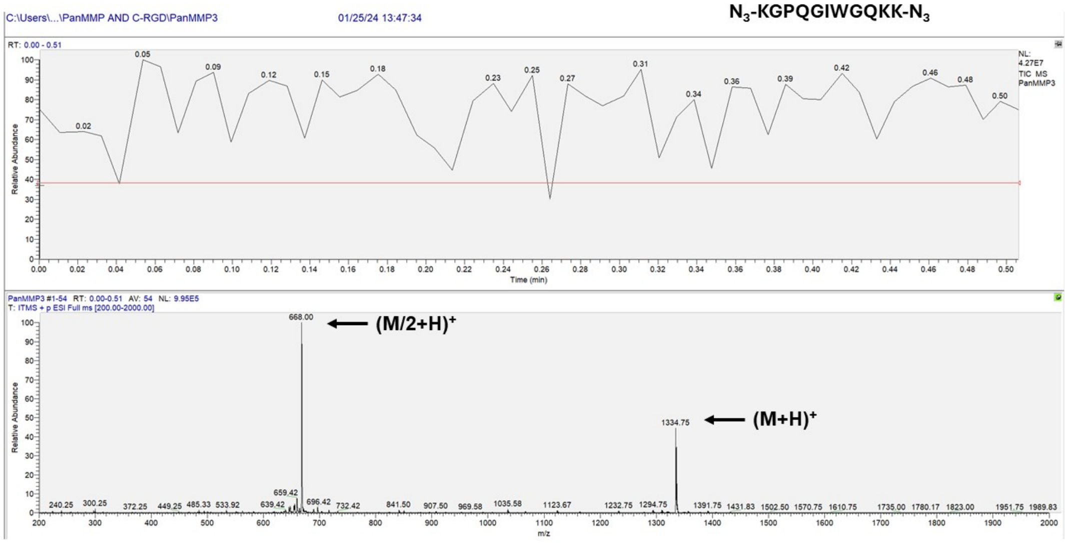

B

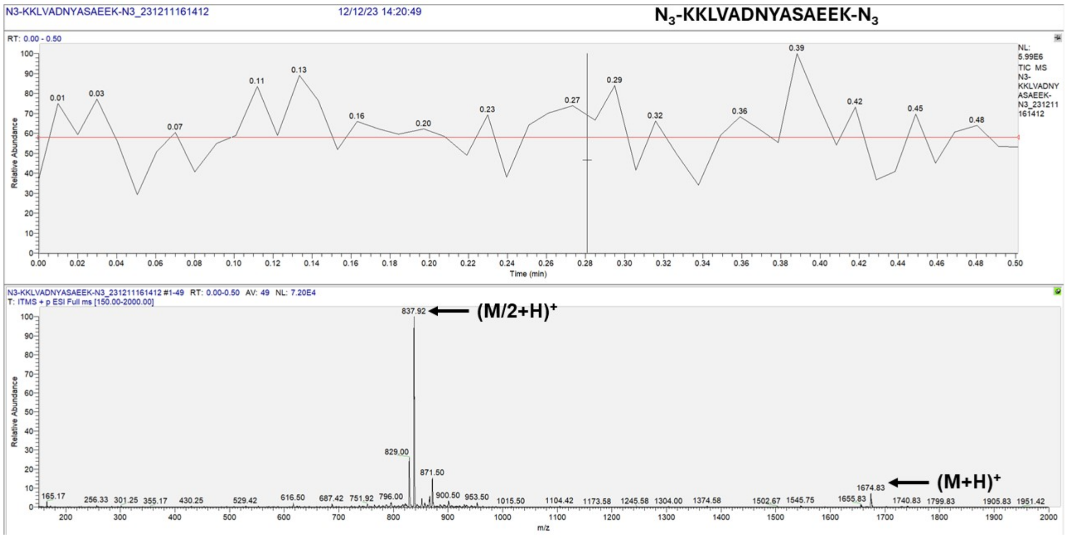

C

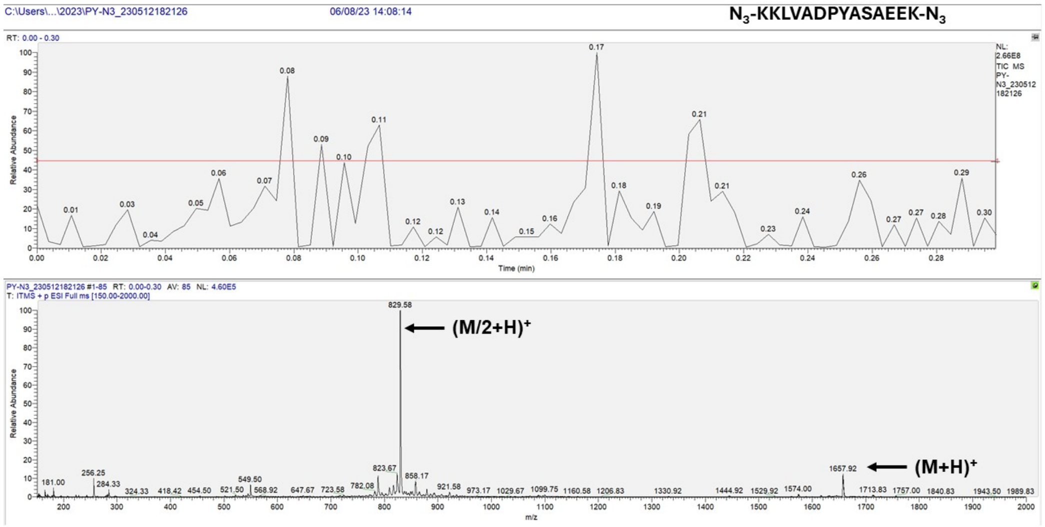
C:\Users\...\2023\PY-N3_230512182126
06/08/23 14:08:14
N3-KKLVADPYASAEEK-N3
Relative Abundance
Time (min)
(M/2+H)+
(M+H)+
829.58
1657.92
m/z

D

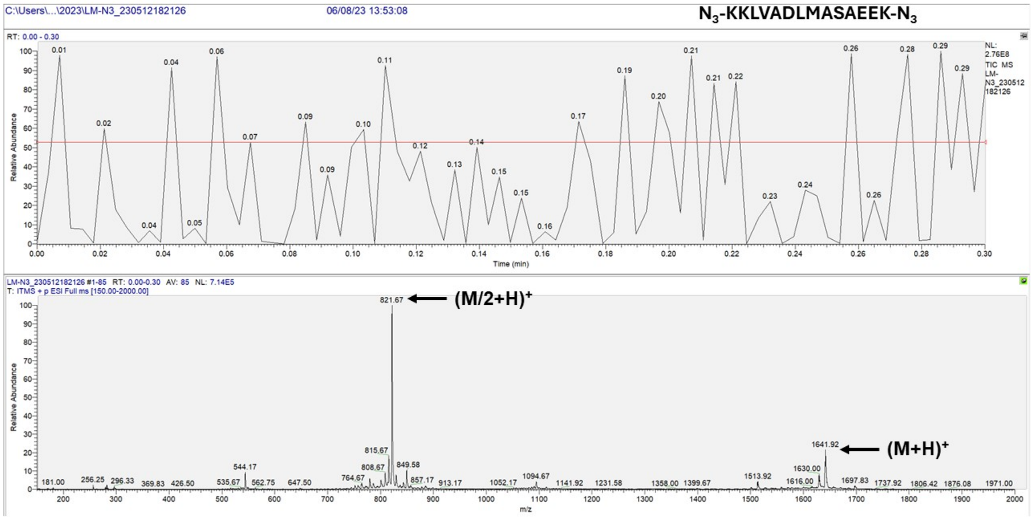
C:\Users\...\2023\LM-N3_230512182126
06/08/23 13:53:08
N3-KKLVADLMASAEEK-N3
Relative Abundance
Time (min)
(M/2+H)+
(M+H)+
821.67
1641.92
m/z

E

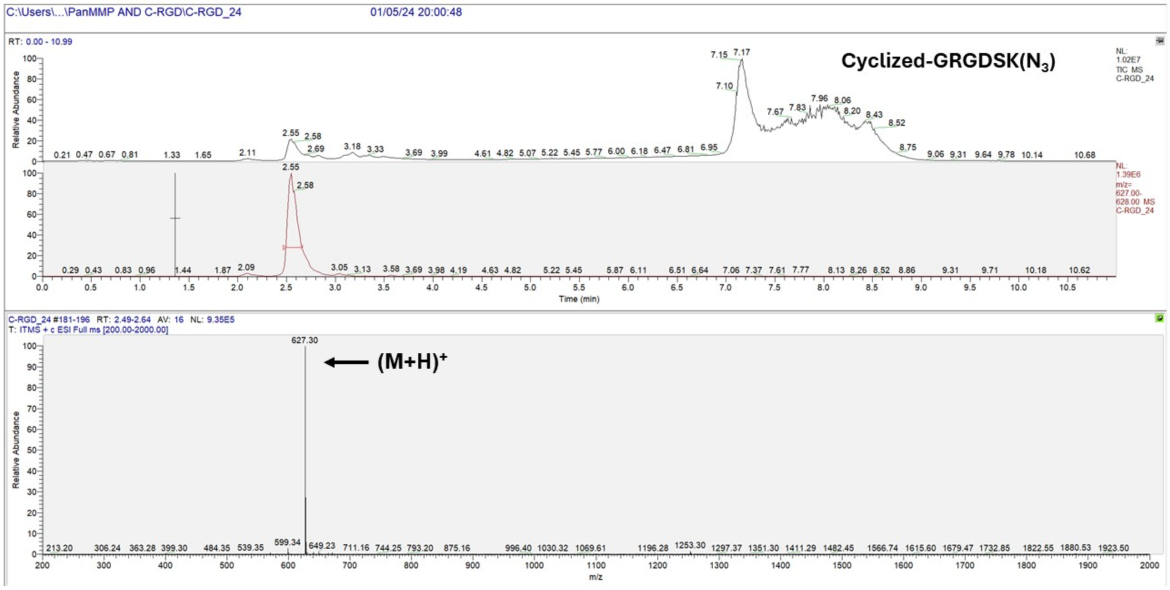
C:\Users\...\PanMMP AND C-RGD\C-RGD_24
01/05/24 20:00:48
RT: 0.00 - 10.99
Cyclized-GRGDSK(N$_3$)
NL: 1.02E7 TIC MS C-RGD_24
NL: 1.39E6 m/z= 627.00-628.00 MS C-RGD_24
Relative Abundance
Time (min)
C-RGD_24 #181-196 RT: 2.49-2.64 AV: 16 NL: 9.35E5
T: ITMS + c ESI Full ms [200.00-2000.00]
627.30
(M+H)$^+$
m/z

F

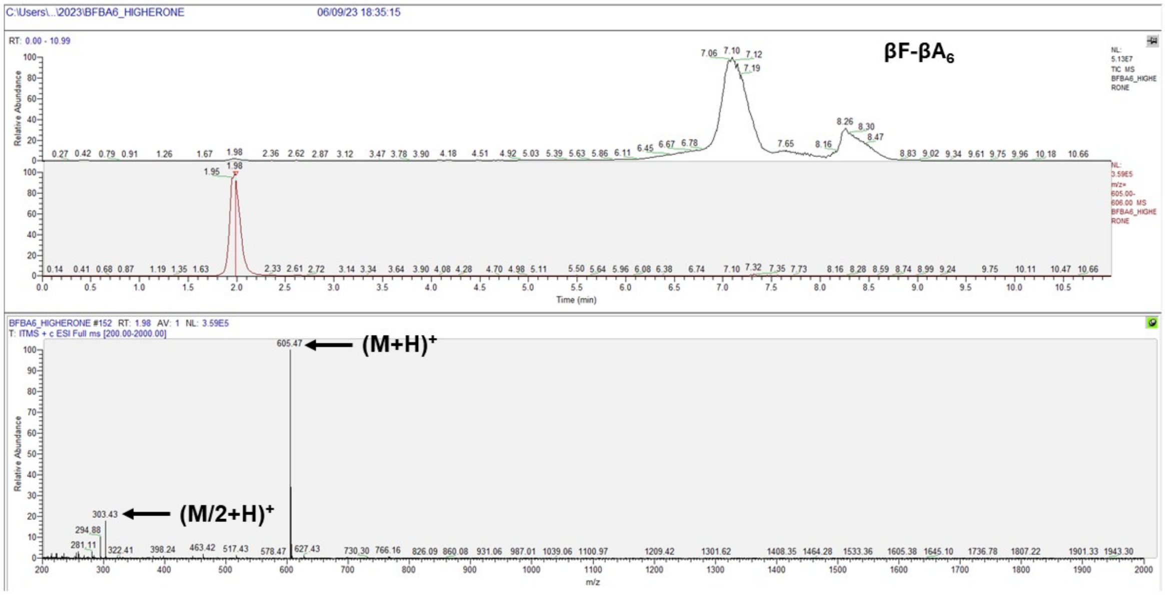
C:\Users\...\2023\BFBA6_HIGHERONE
06/09/23 18:35:15
RT: 0.00 - 10.99
βF-βA$_6$
NL: 5.13E7 TIC MS BFBA6_HIGHERONE
NL: 3.59E5 m/z= 605.00-606.00 MS BFBA6_HIGHERONE
Relative Abundance
Time (min)
BFBA6_HIGHERONE #152 RT: 1.98 AV: 1 NL: 3.59E5
T: ITMS + c ESI Full ms [200.00-2000.00]
605.47
(M+H)$^+$
303.43
(M/2+H)$^+$
m/z

G

Cy3-KKKLVADLMASAEKKK(N_3)

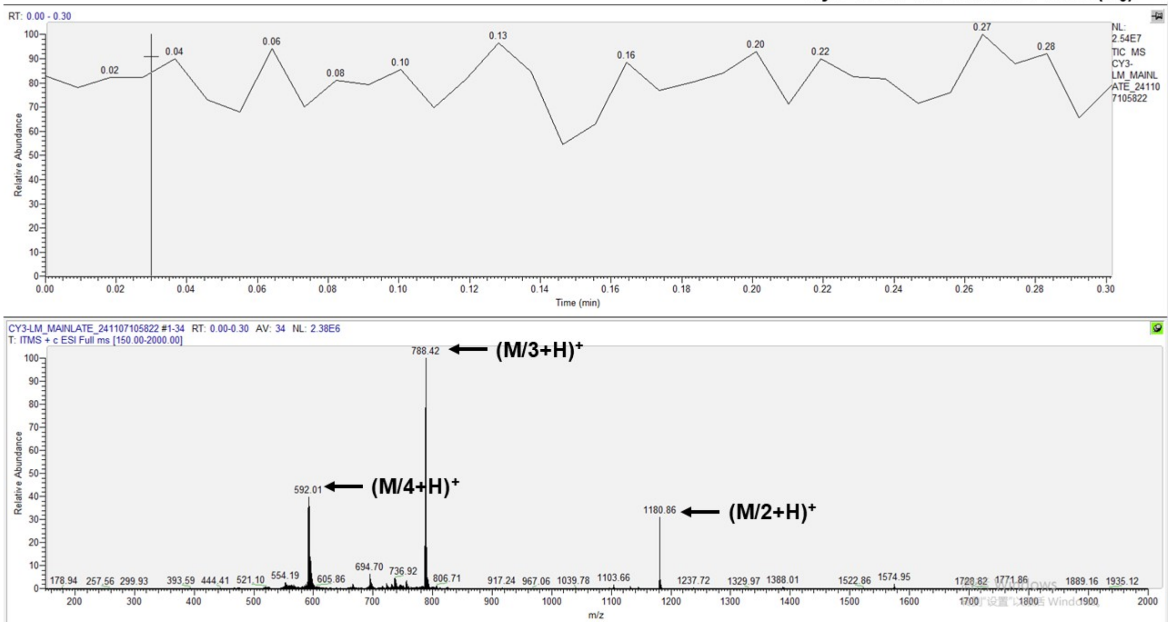

H

Cy3-KKGPQGIWGQKKK(N_3)

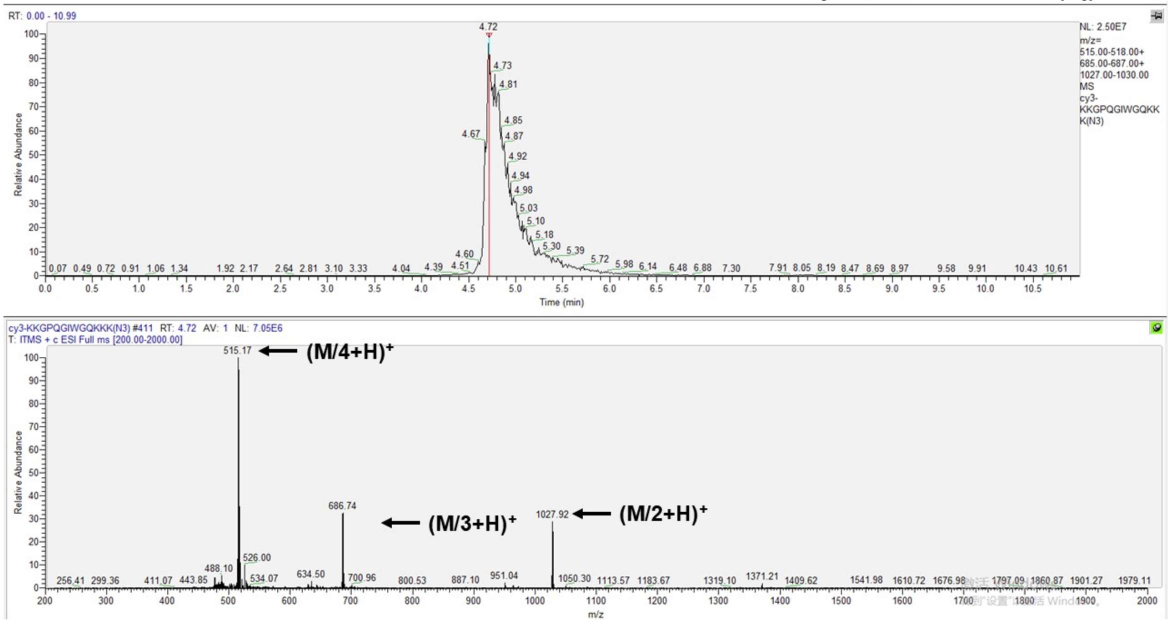

Fig. S8. Mass spec characterization of peptides. Crosslinking peptides (**A**) N_3-KGPQGIWGQK(N_3), (**B**) N_3-KKLVADNYASAEEK(N_3) (**C**) N_3-KKLVADPYASAEEK(N_3), (**D**) N_3-KKLVADLMASAEEK(N_3), (**E**) cyclic GRGDSK(N_3), (**F**) the βF-βA_6, (**G**) Cy3-KKKLVADLMASAEKKK(N_3), and (**H**) N_3-KKGPQGIWGQKKK(N_3) internal standard for degradation studies.

Characterization of KLVAD-XX-ASAE split-and-pool peptide libraries

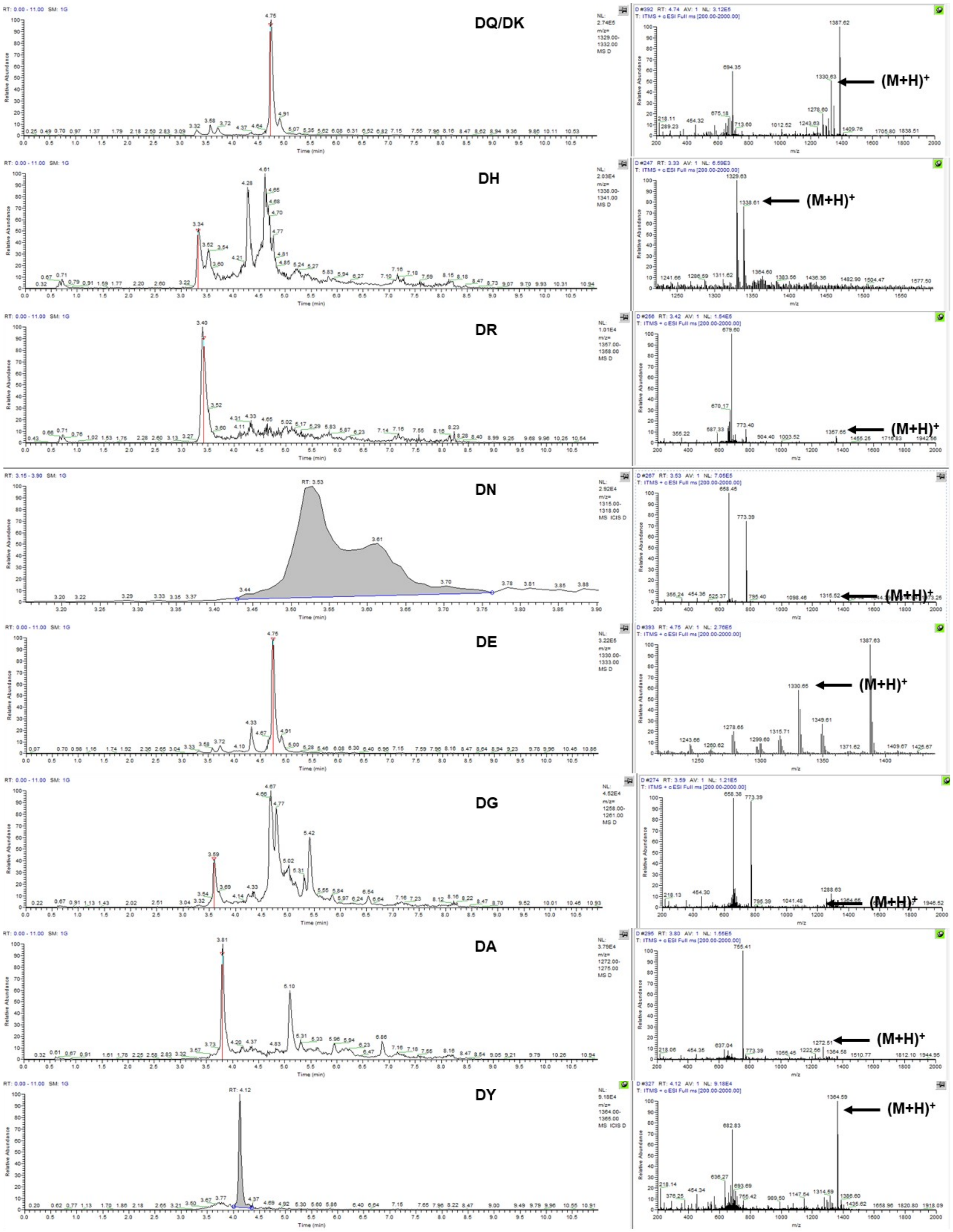

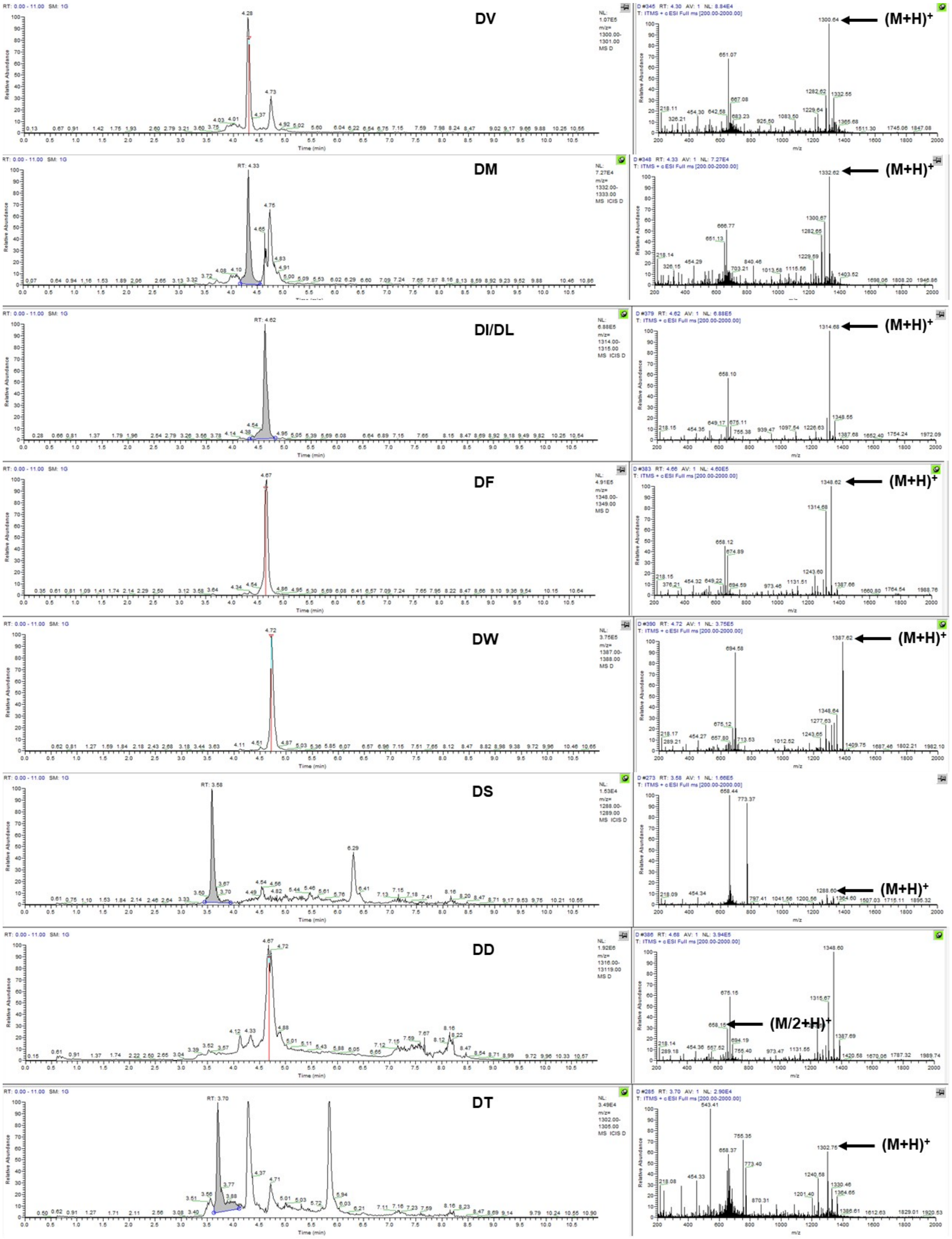
DV
(M+H)+
DM
(M+H)+
DI/DL
(M+H)+
DF
(M+H)+
DW
(M+H)+
DS
(M+H)+
DD
(M/2+H)+
DT
(M+H)+
Relative Abundance
Time (min)
m/z

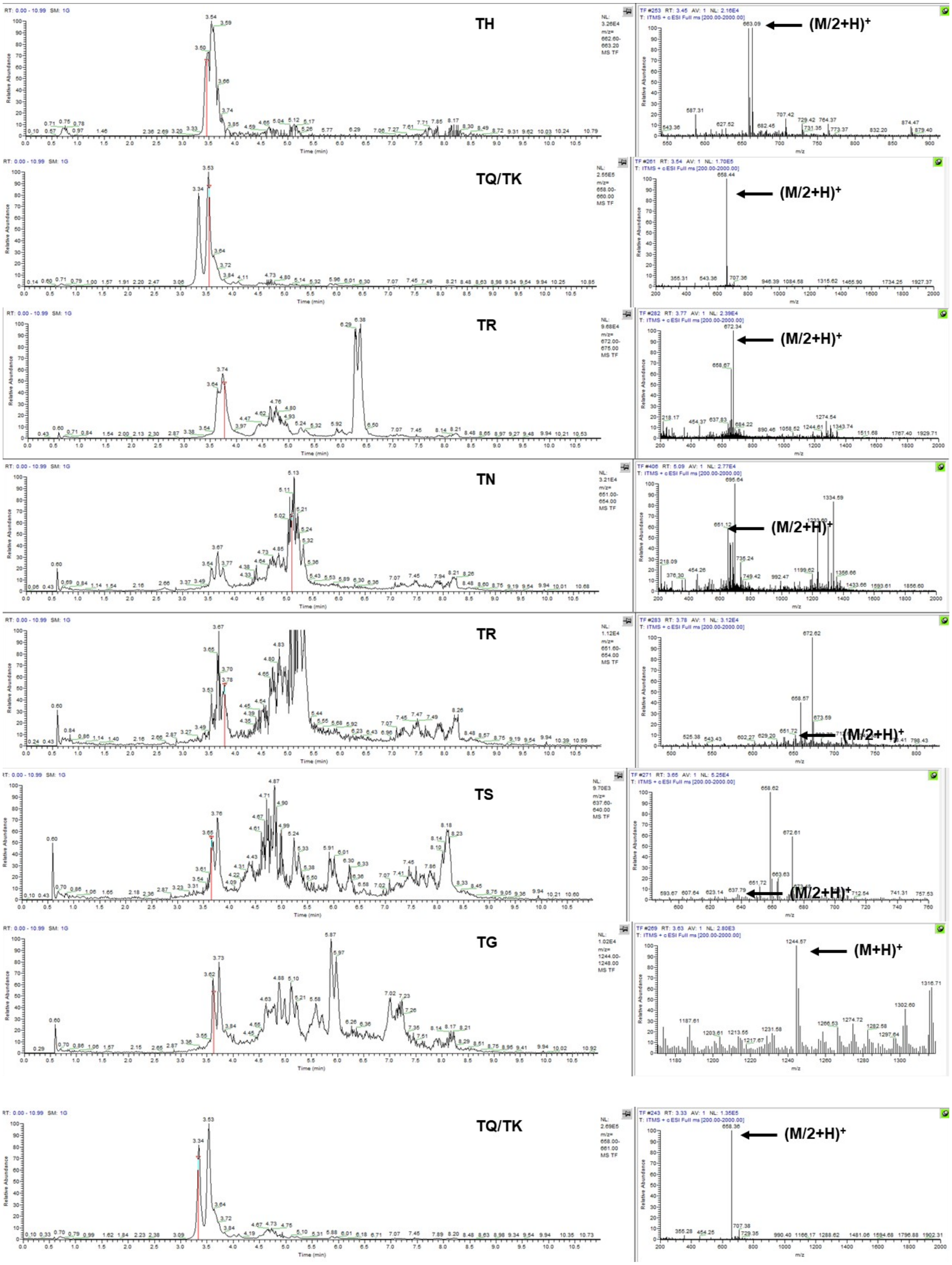

TH
(M/2+H)+
TQ/TK
(M/2+H)+
TR
(M/2+H)+
TN
(M/2+H)+
TR
(M/2+H)+
TS
(M/2+H)+
TG
(M+H)+
TQ/TK
(M/2+H)+

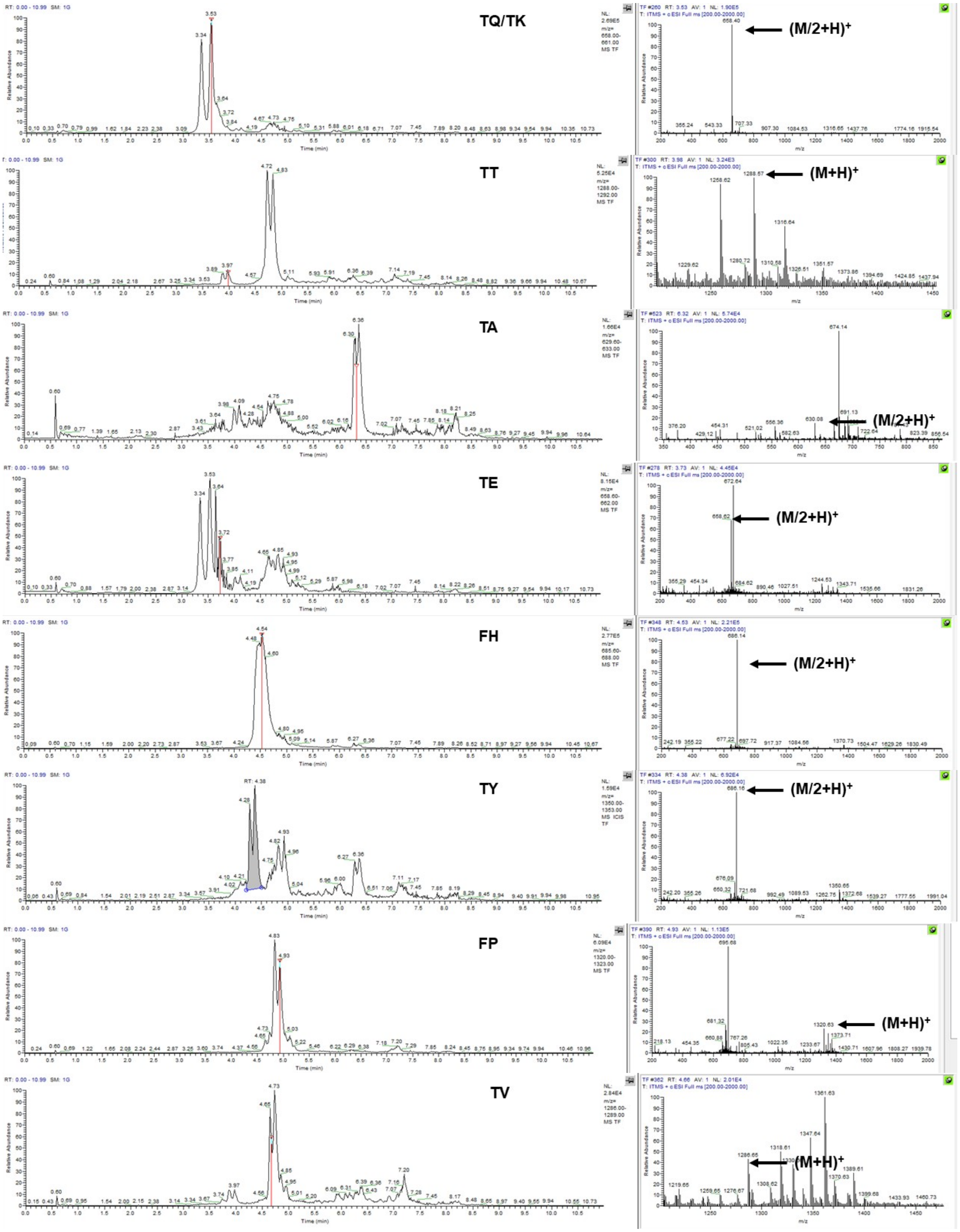

TQ/TK
(M/2+H)+
TT
(M+H)+
TA
(M/2+H)+
TE
(M/2+H)+
FH
(M/2+H)+
TY
(M/2+H)+
FP
(M+H)+
TV
(M+H)+

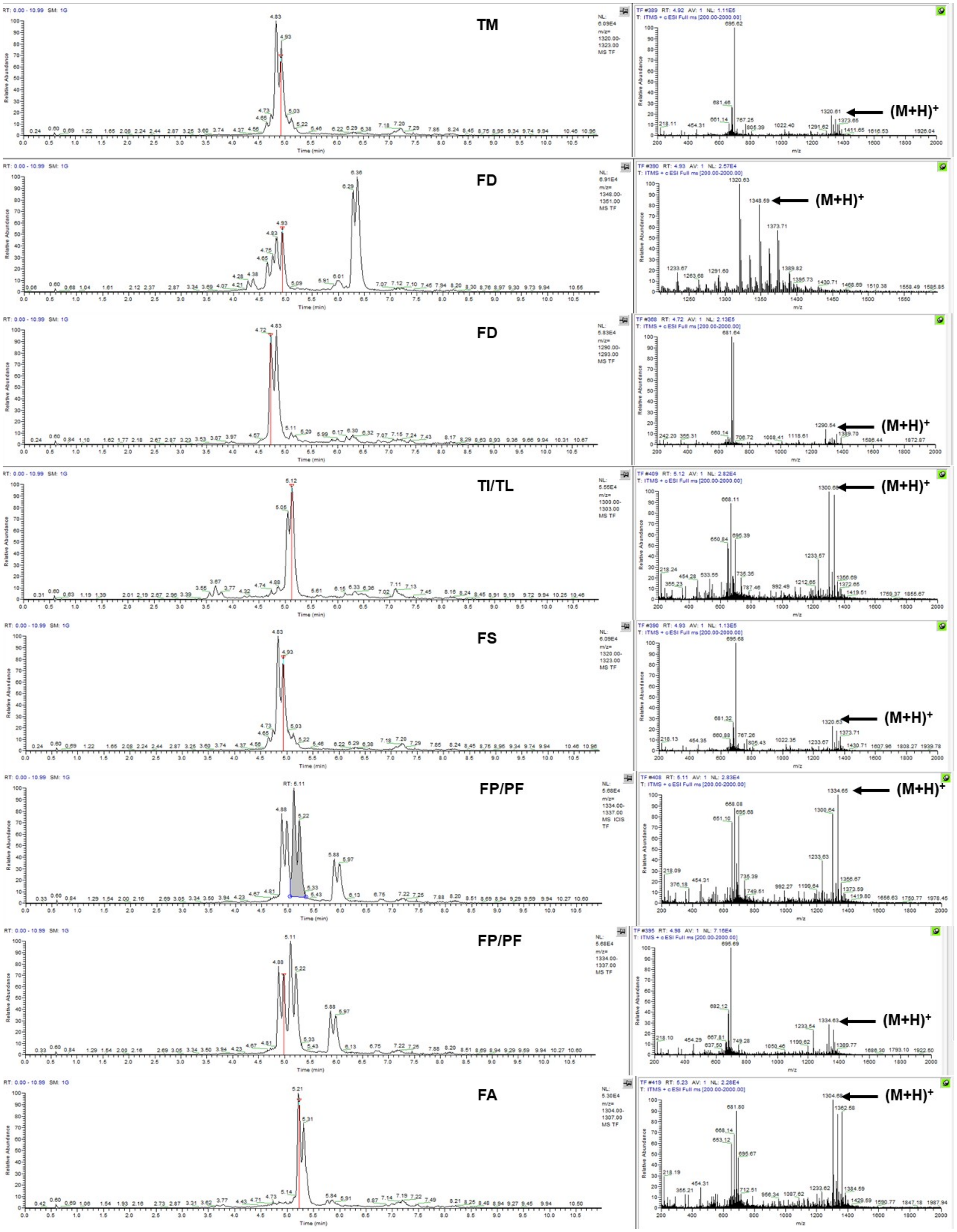

TM
(M+H)+
FD
(M+H)+
FD
(M+H)+
TI/TL
(M+H)+
FS
(M+H)+
FP/PF
(M+H)+
FP/PF
(M+H)+
FA
(M+H)+

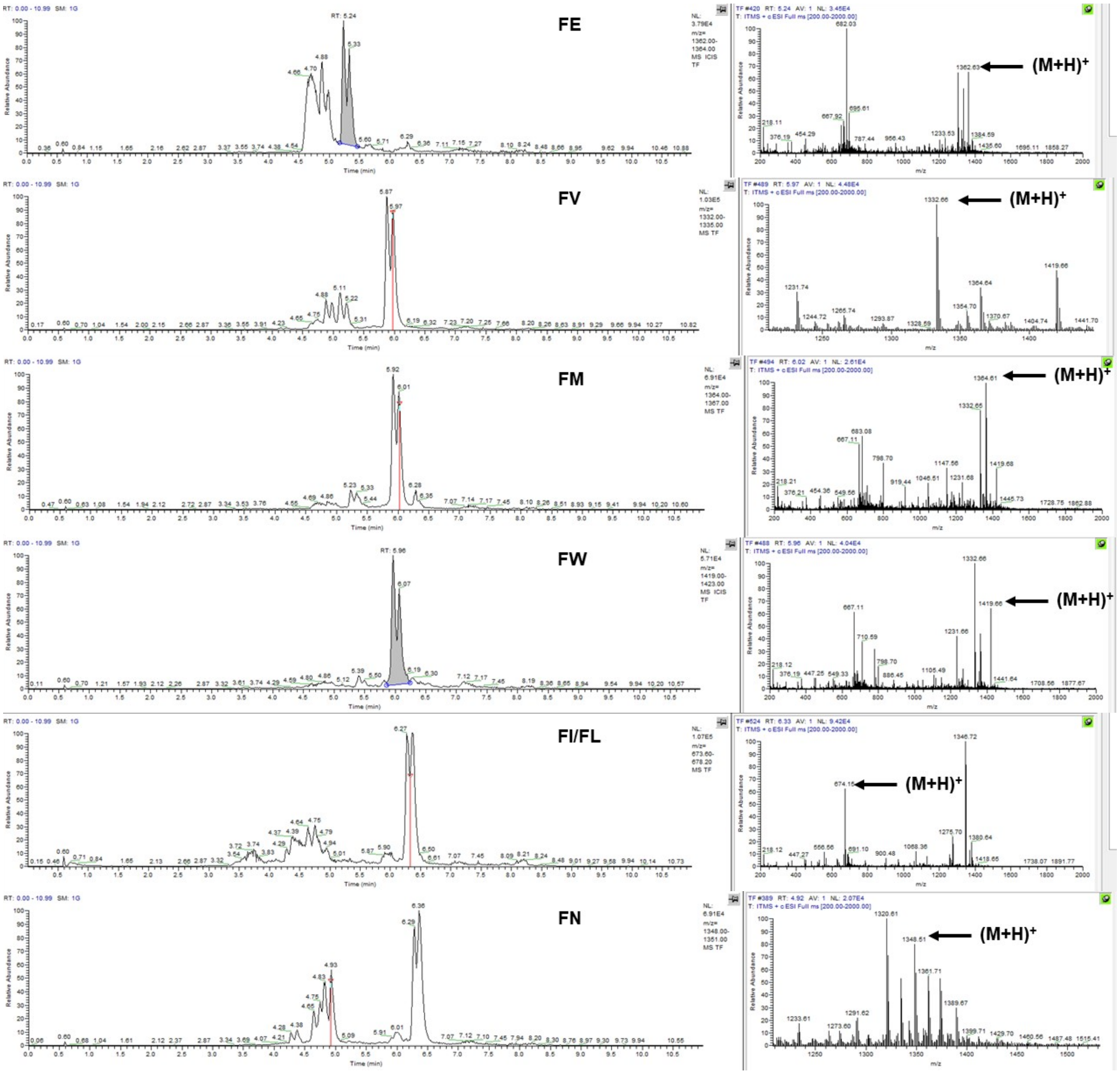
FE
(M+H)+
FV
(M+H)+
FM
(M+H)+
FW
(M+H)+
FI/FL
(M+H)+
FN
(M+H)+
Relative Abundance
Time (min)
m/z

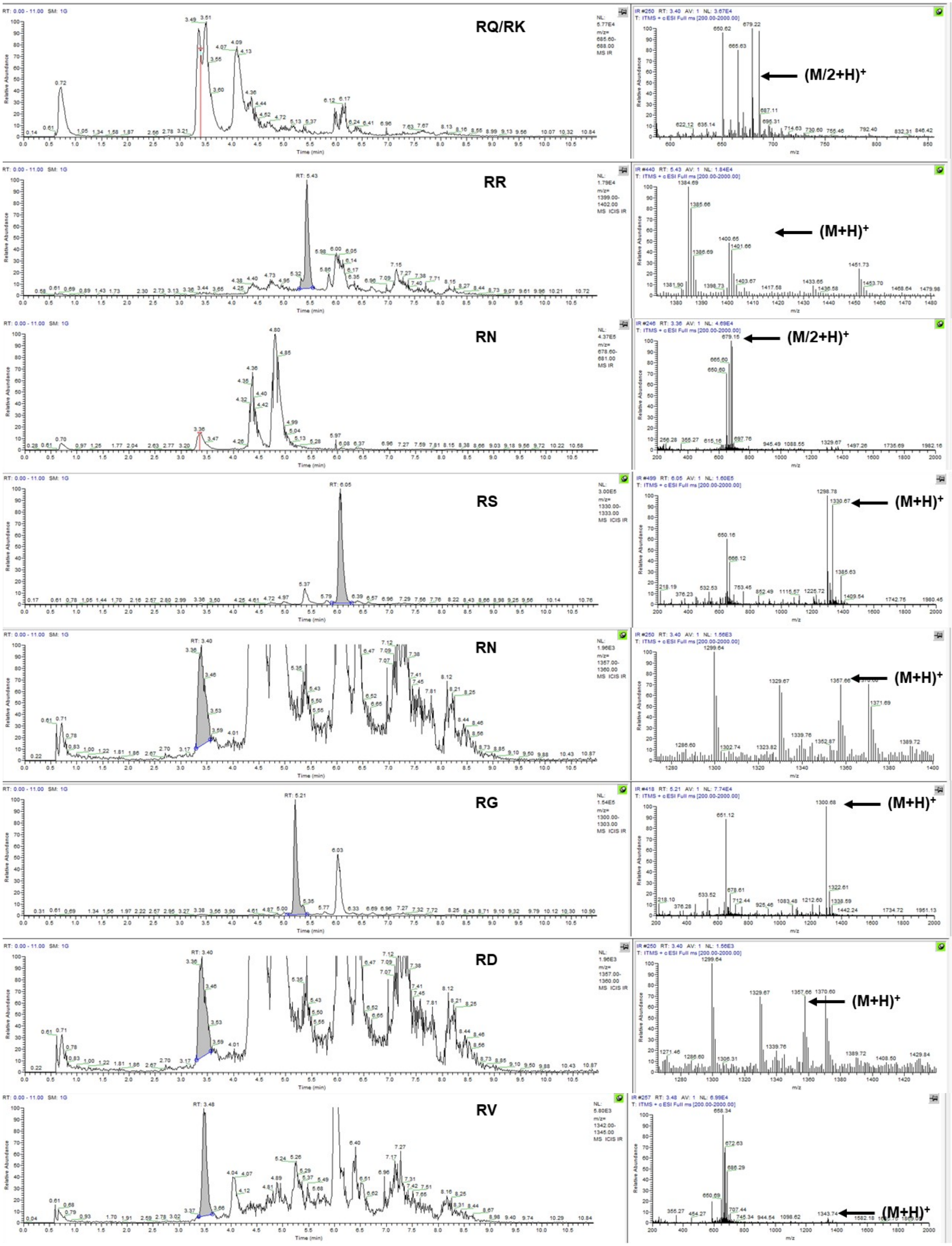
RQ/RK
(M/2+H)+
RR
(M+H)+
RN
(M/2+H)+
RS
(M+H)+
RN
(M+H)+
RG
(M+H)+
RD
(M+H)+
RV
(M+H)+

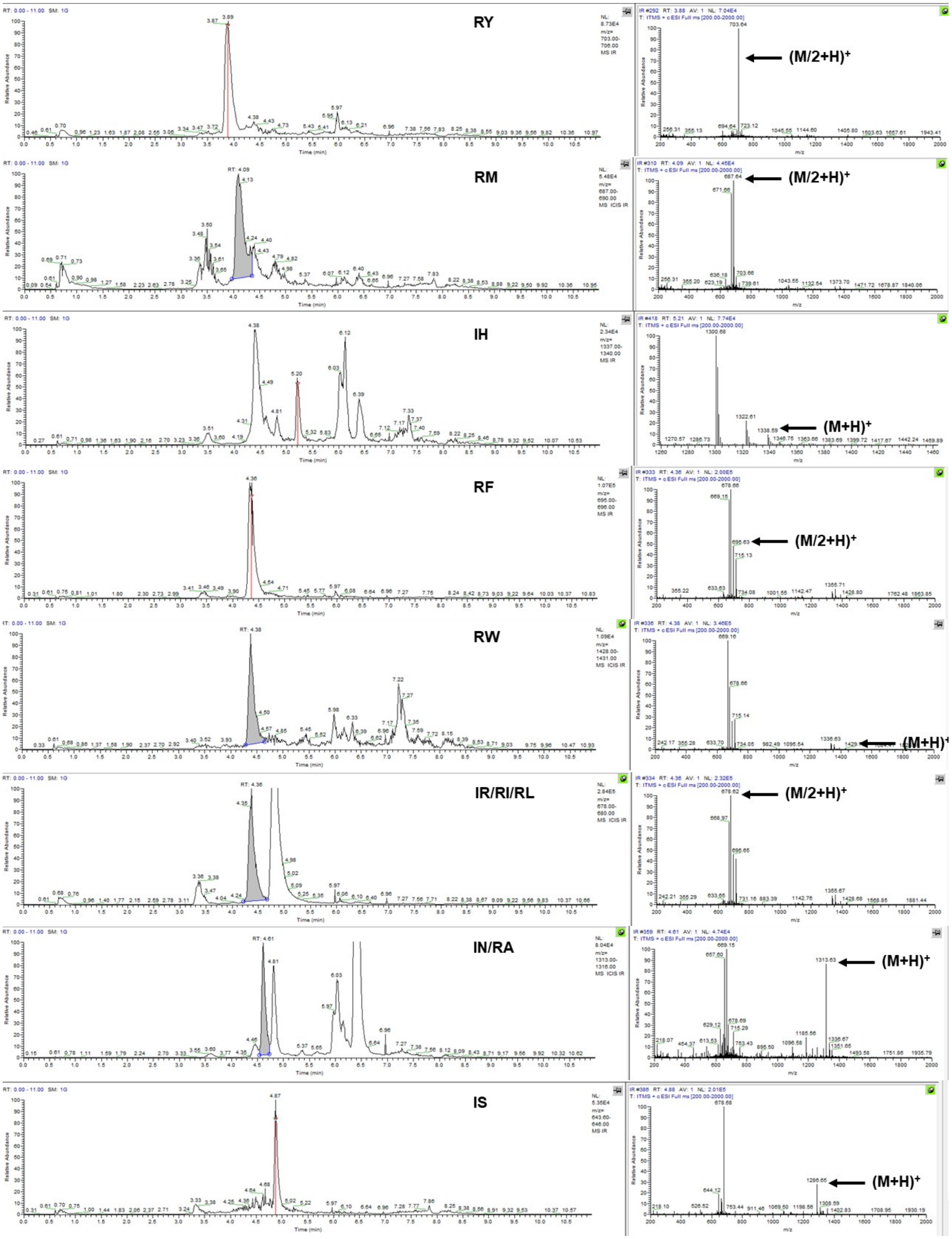

RY
(M/2+H)+
RM
(M/2+H)+
IH
(M+H)+
RF
(M/2+H)+
RW
(M+H)+
IR/RI/RL
(M/2+H)+
IN/RA
(M+H)+
IS
(M+H)+

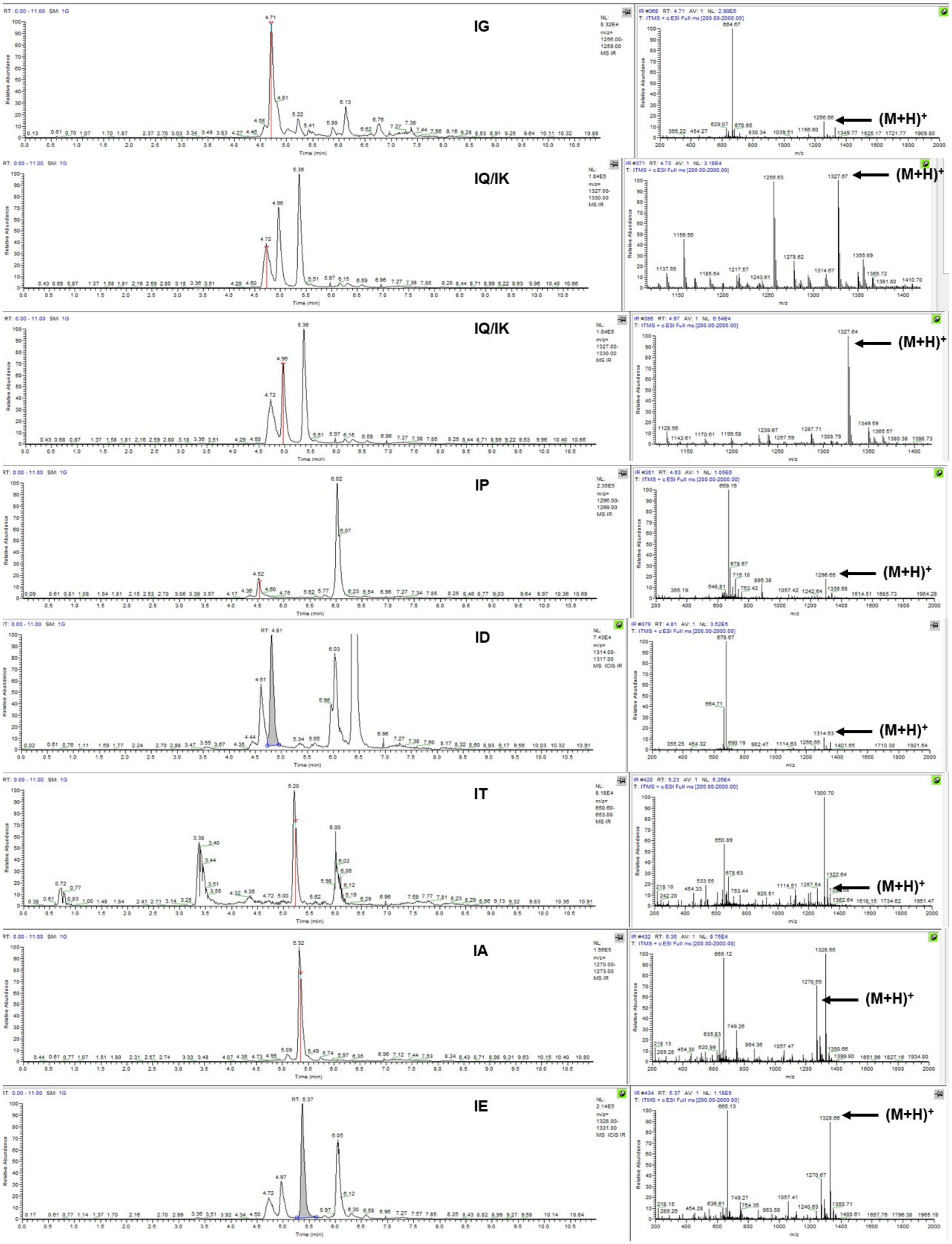
IG
(M+H)+
IQ/IK
(M+H)+
IQ/IK
(M+H)+
IP
(M+H)+
ID
(M+H)+
IT
(M+H)+
IA
(M+H)+
IE
(M+H)+

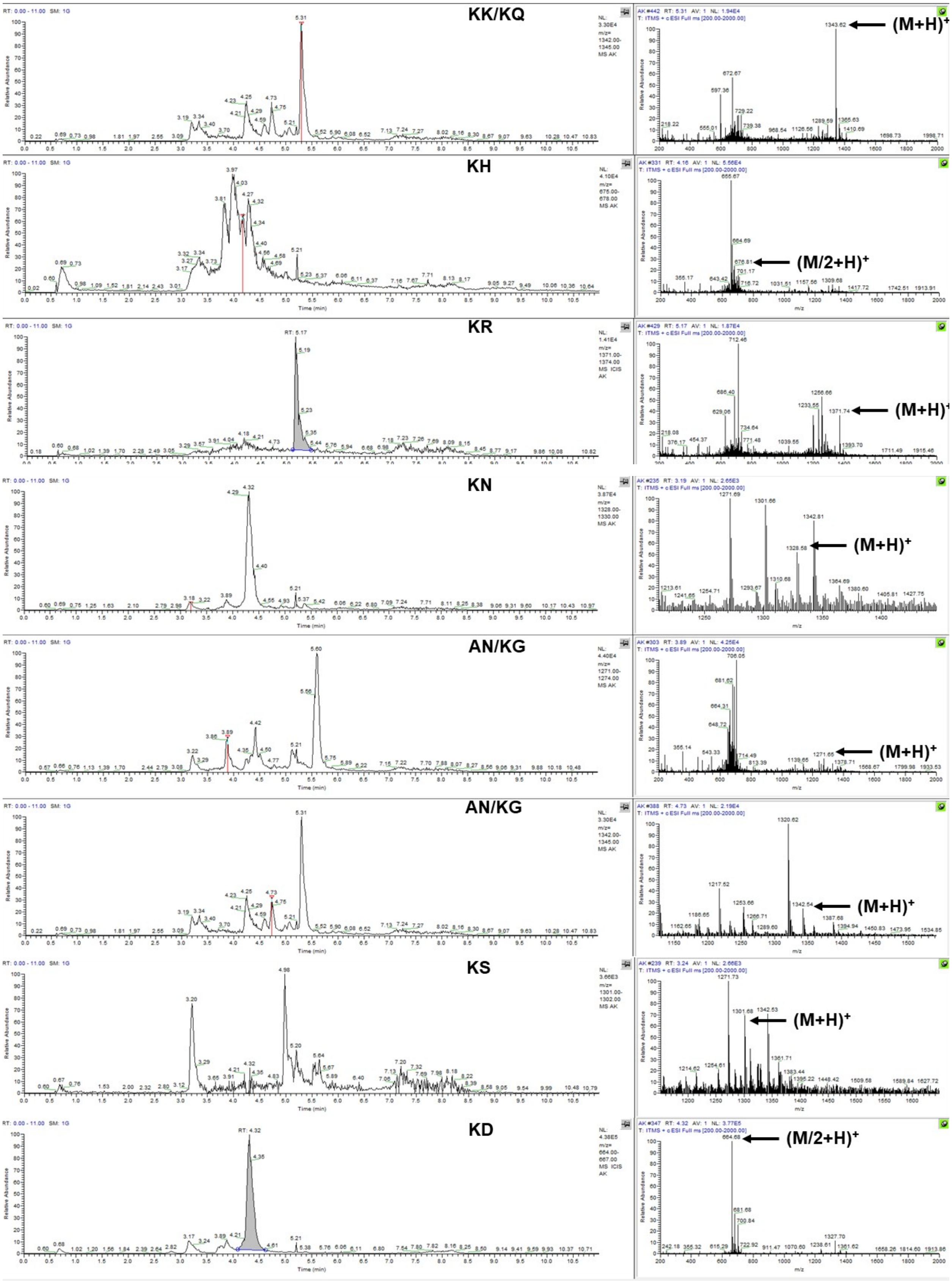
KK/KQ
(M+H)+
KH
(M/2+H)+
KR
(M+H)+
KN
(M+H)+
AN/KG
(M+H)+
AN/KG
(M+H)+
KS
(M+H)+
KD
(M/2+H)+

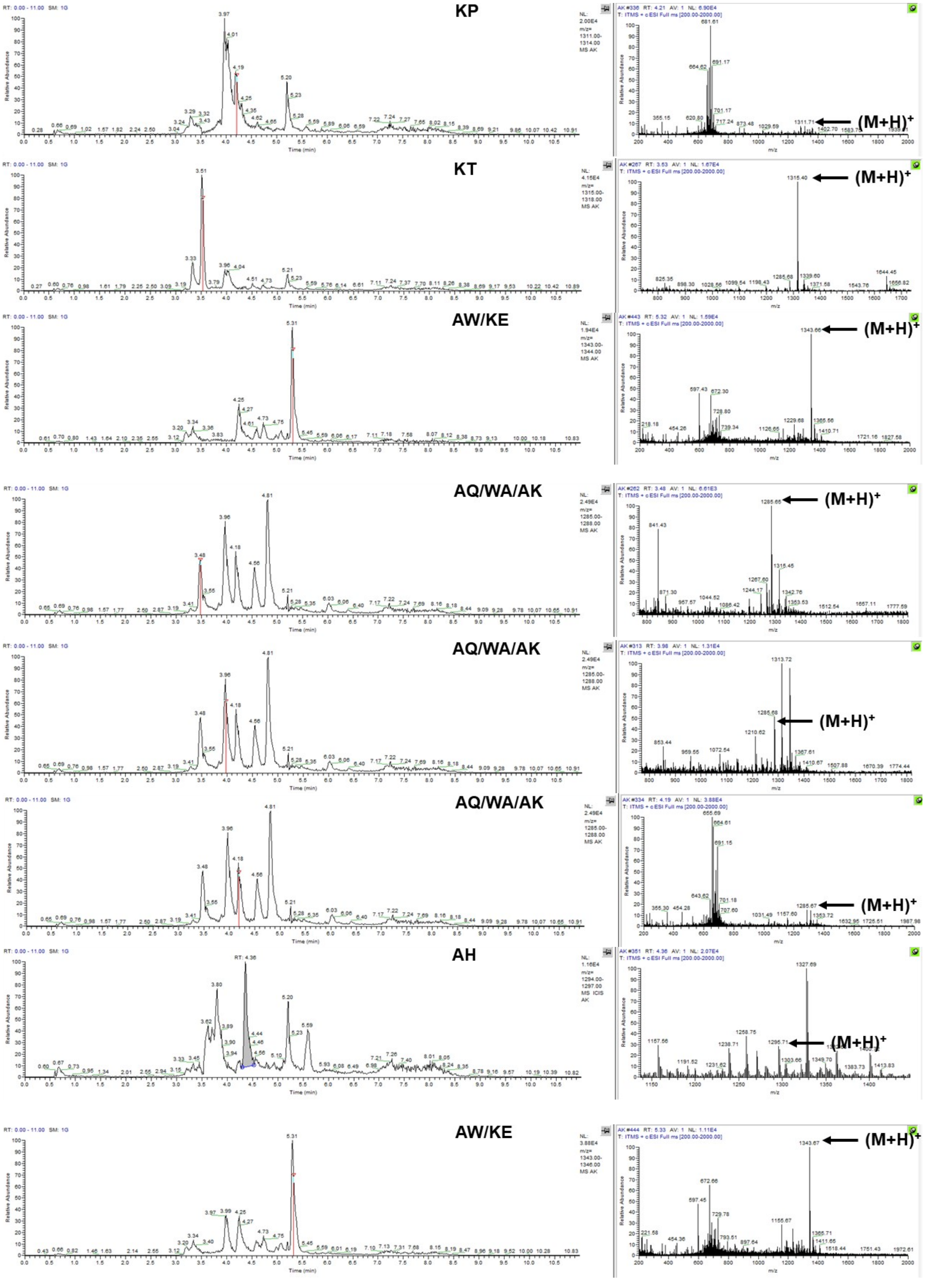

KP
(M+H)+
KT
(M+H)+
AW/KE
(M+H)+
AQ/WA/AK
(M+H)+
AQ/WA/AK
(M+H)+
AQ/WA/AK
(M+H)+
AH
(M+H)+
AW/KE
(M+H)+

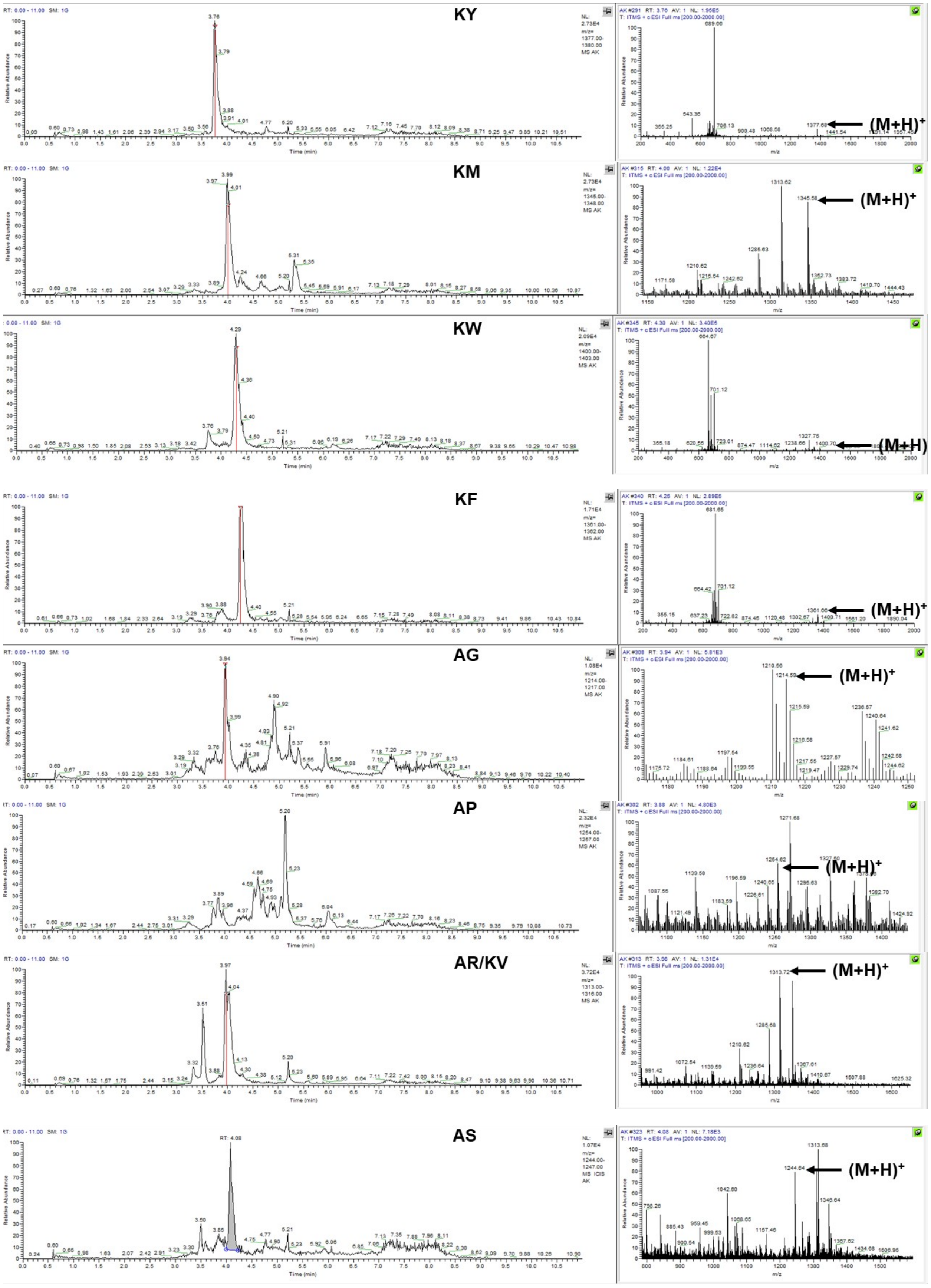
KY
(M+H)+
KM
(M+H)+
KW
(M+H)
KF
(M+H)+
AG
(M+H)+
AP
(M+H)+
AR/KV
(M+H)+
AS
(M+H)+
Relative Abundance
Time (min)
m/z

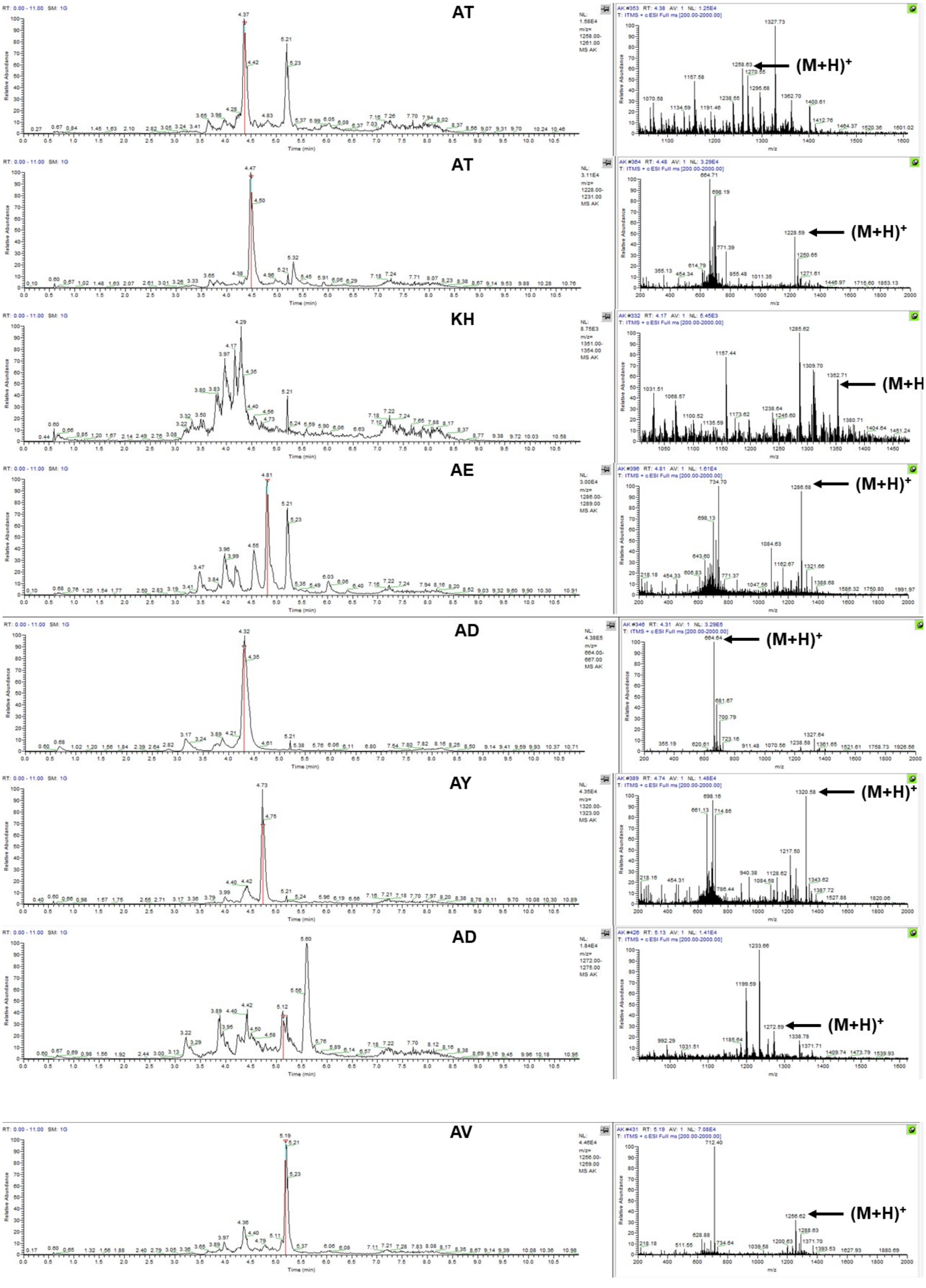
AT
(M+H)+
AT
(M+H)+
KH
(M+H
AE
(M+H)+
AD
(M+H)+
AY
(M+H)+
AD
(M+H)+
AV
(M+H)+

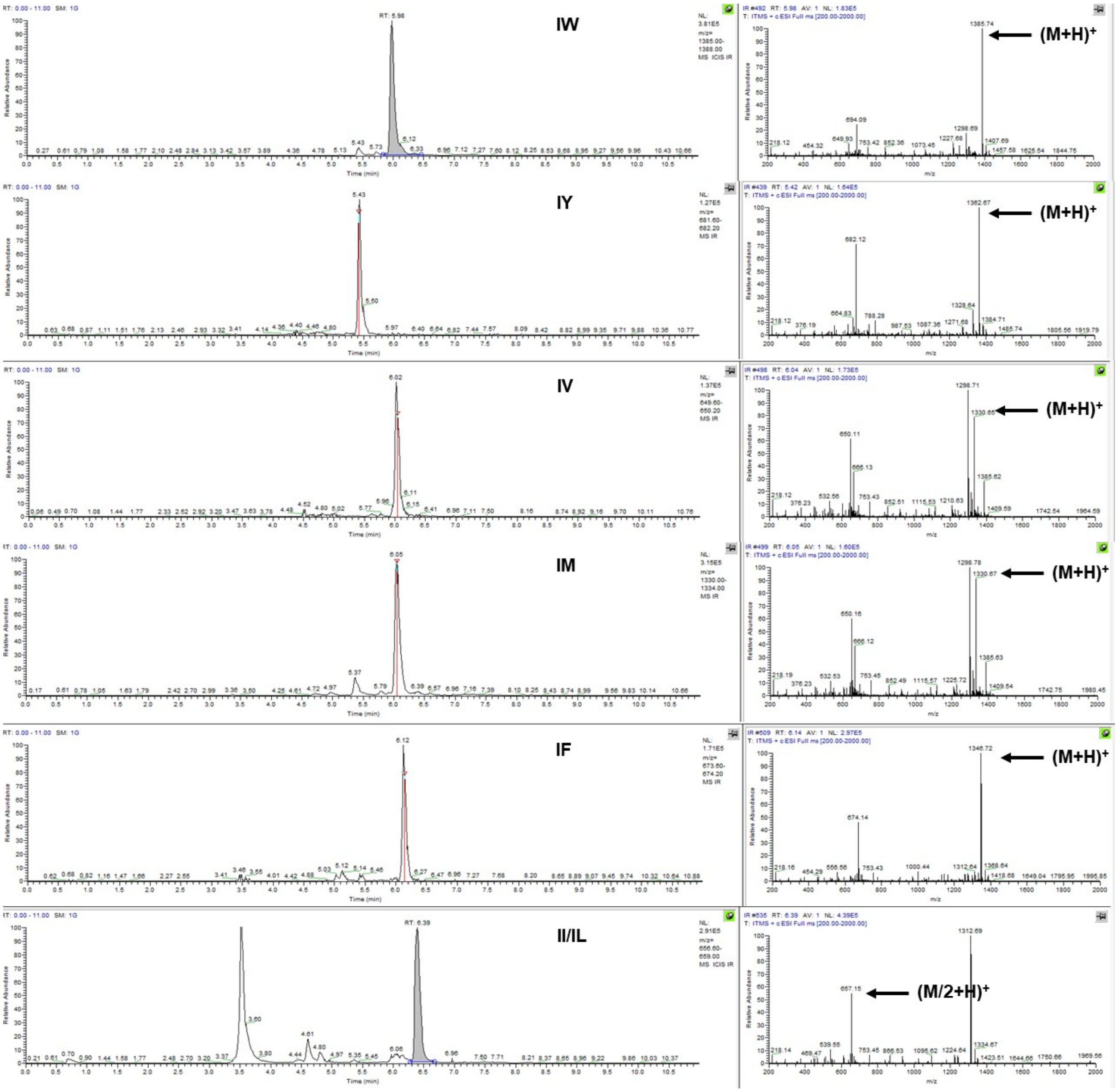

IW
(M+H)+
1385.74
IY
(M+H)+
1362.67
682.12
IV
(M+H)+
1298.71
1330.65
650.11
IM
(M+H)+
1298.78
1330.67
650.16
IF
(M+H)+
1346.72
674.14
II/IL
(M/2+H)+
1312.69
657.15
Relative Abundance
Time (min)
m/z

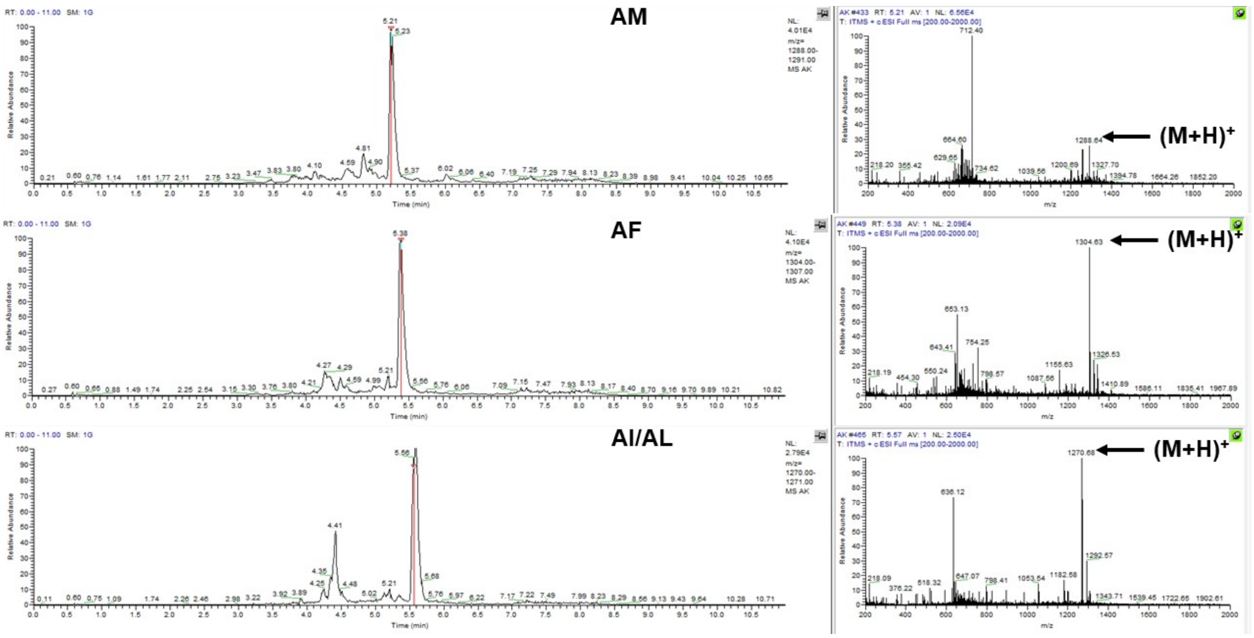
AM
(M+H)+
AF
(M+H)+
AI/AL
(M+H)+

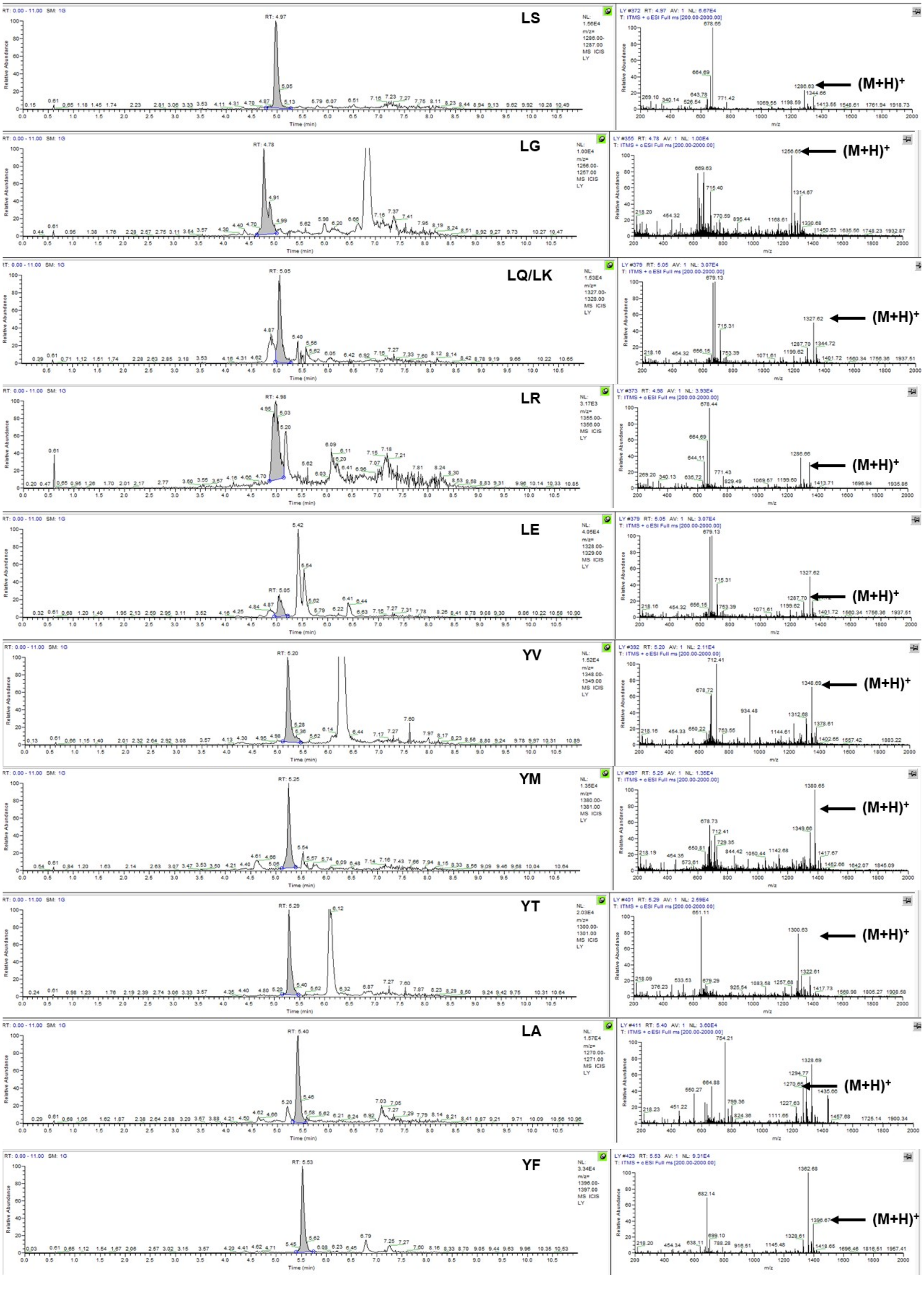

LS
(M+H)+
LG
(M+H)+
LQ/LK
(M+H)+
LR
(M+H)+
LE
(M+H)+
YV
(M+H)+
YM
(M+H)+
YT
(M+H)+
LA
(M+H)+
YF
(M+H)+

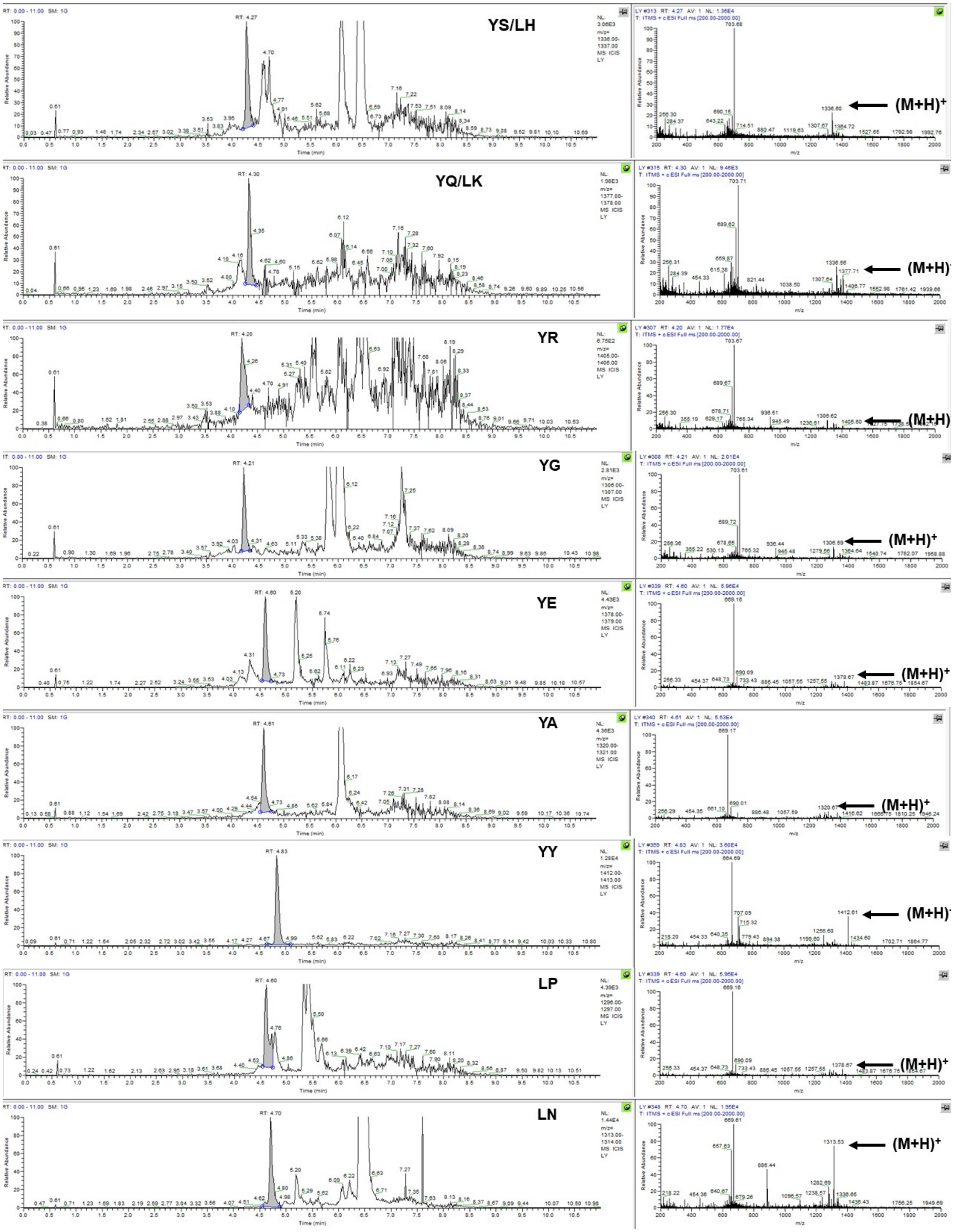
YS/LH
(M+H)+
YQ/LK
(M+H)+
YR
(M+H)+
YG
(M+H)+
YE
(M+H)+
YA
(M+H)+
YY
(M+H)+
LP
(M+H)+
LN
(M+H)+

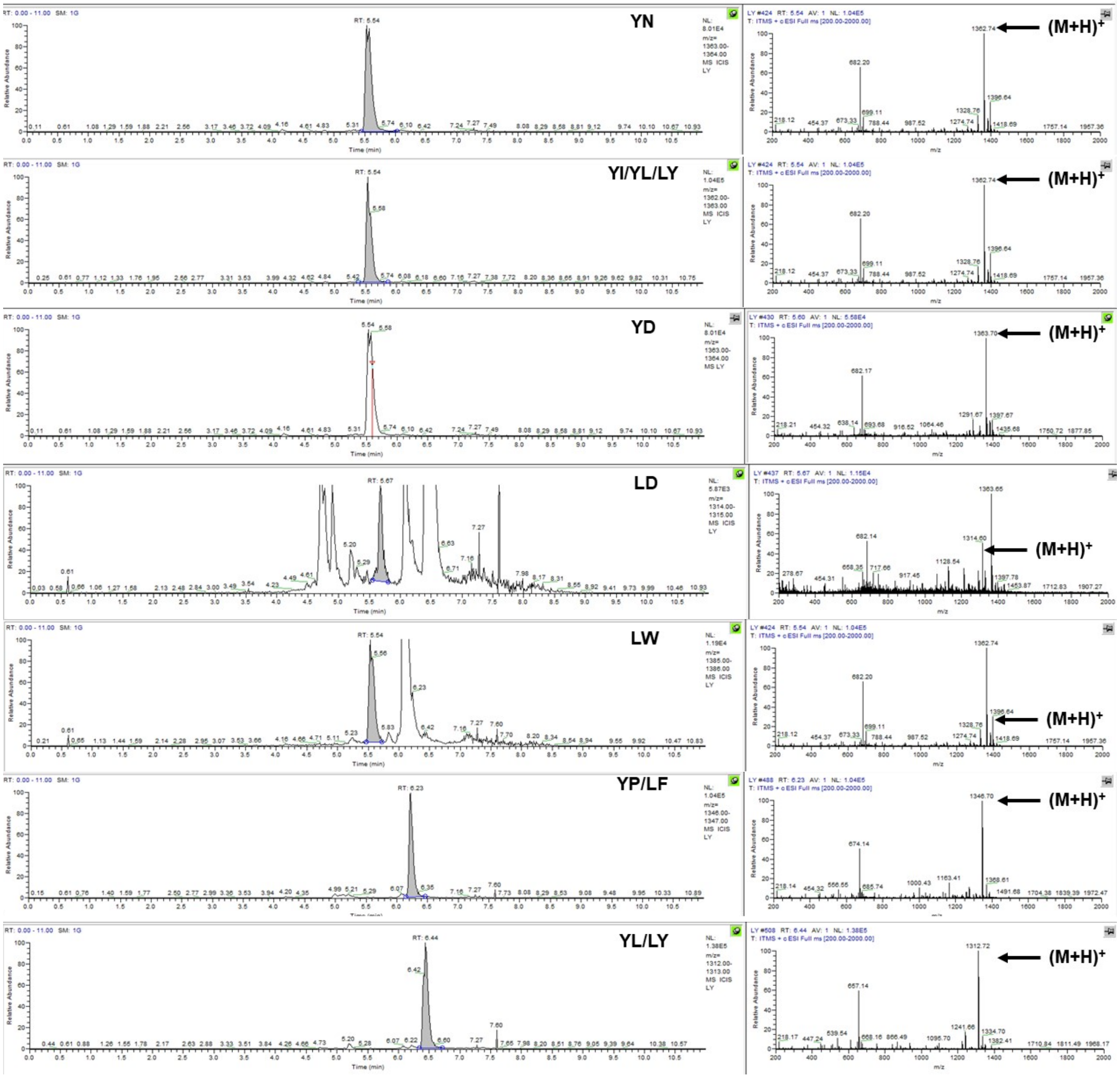
YN
(M+H)+
YI/YL/LY
(M+H)+
YD
(M+H)+
LD
(M+H)+
LW
(M+H)+
YP/LF
(M+H)+
YL/LY
(M+H)+

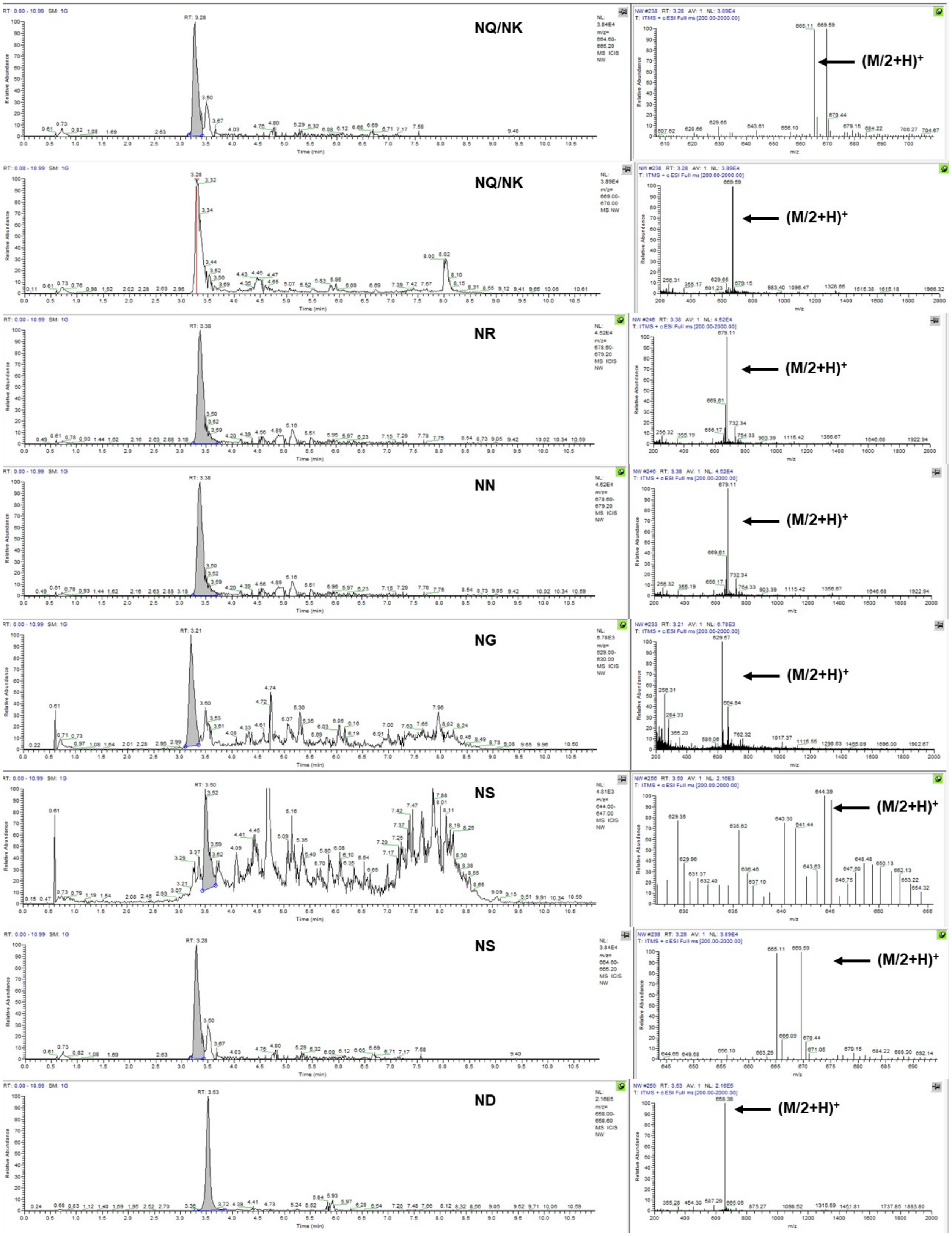
NQ/NK
(M/2+H)+
NQ/NK
(M/2+H)+
NR
(M/2+H)+
NN
(M/2+H)+
NG
(M/2+H)+
NS
(M/2+H)+
NS
(M/2+H)+
ND
(M/2+H)+

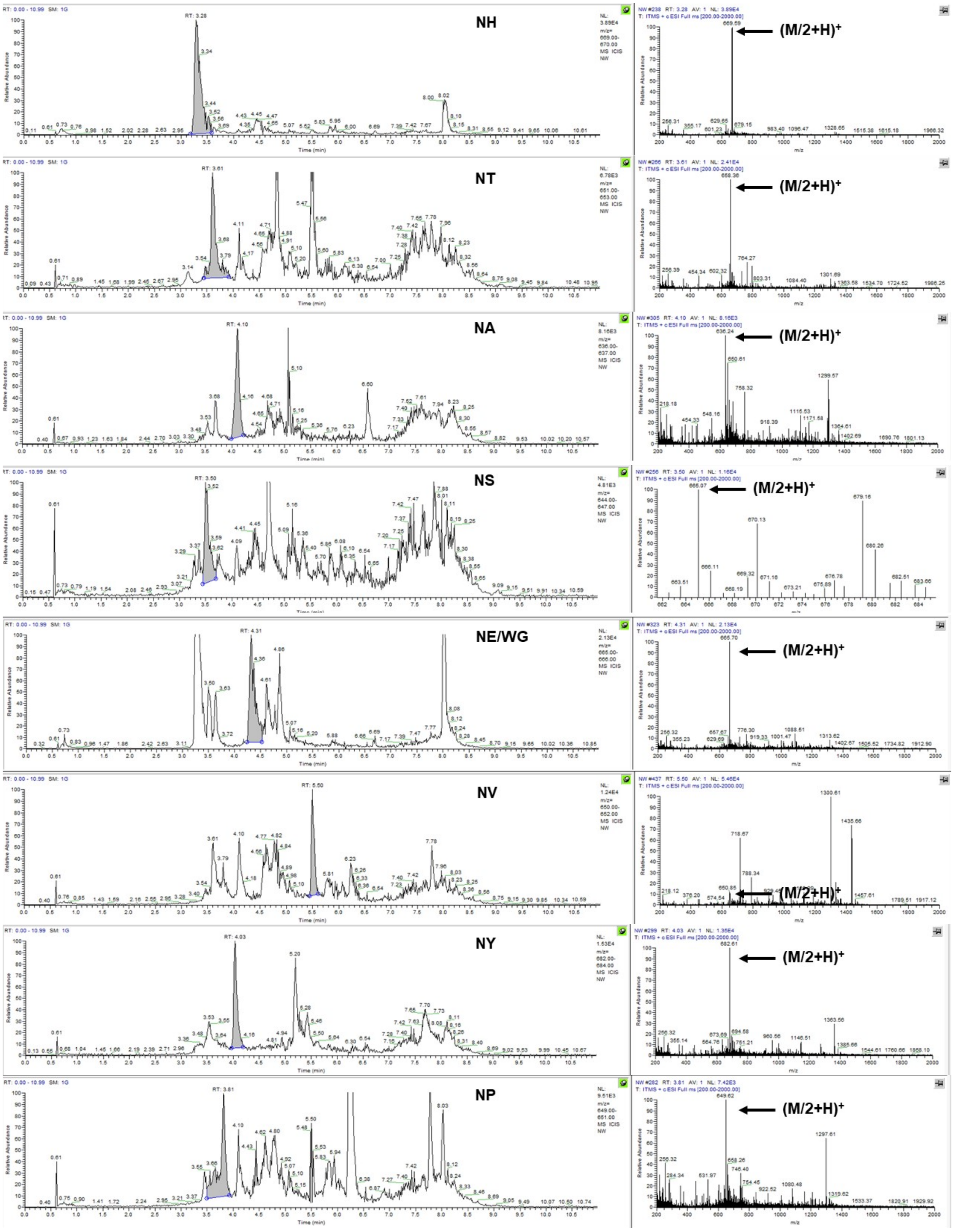
NH
(M/2+H)+
NT
(M/2+H)+
NA
(M/2+H)+
NS
(M/2+H)+
NE/WG
(M/2+H)+
NV
(M/2+H)+
NY
(M/2+H)+
NP
(M/2+H)+
Relative Abundance
Time (min)
m/z

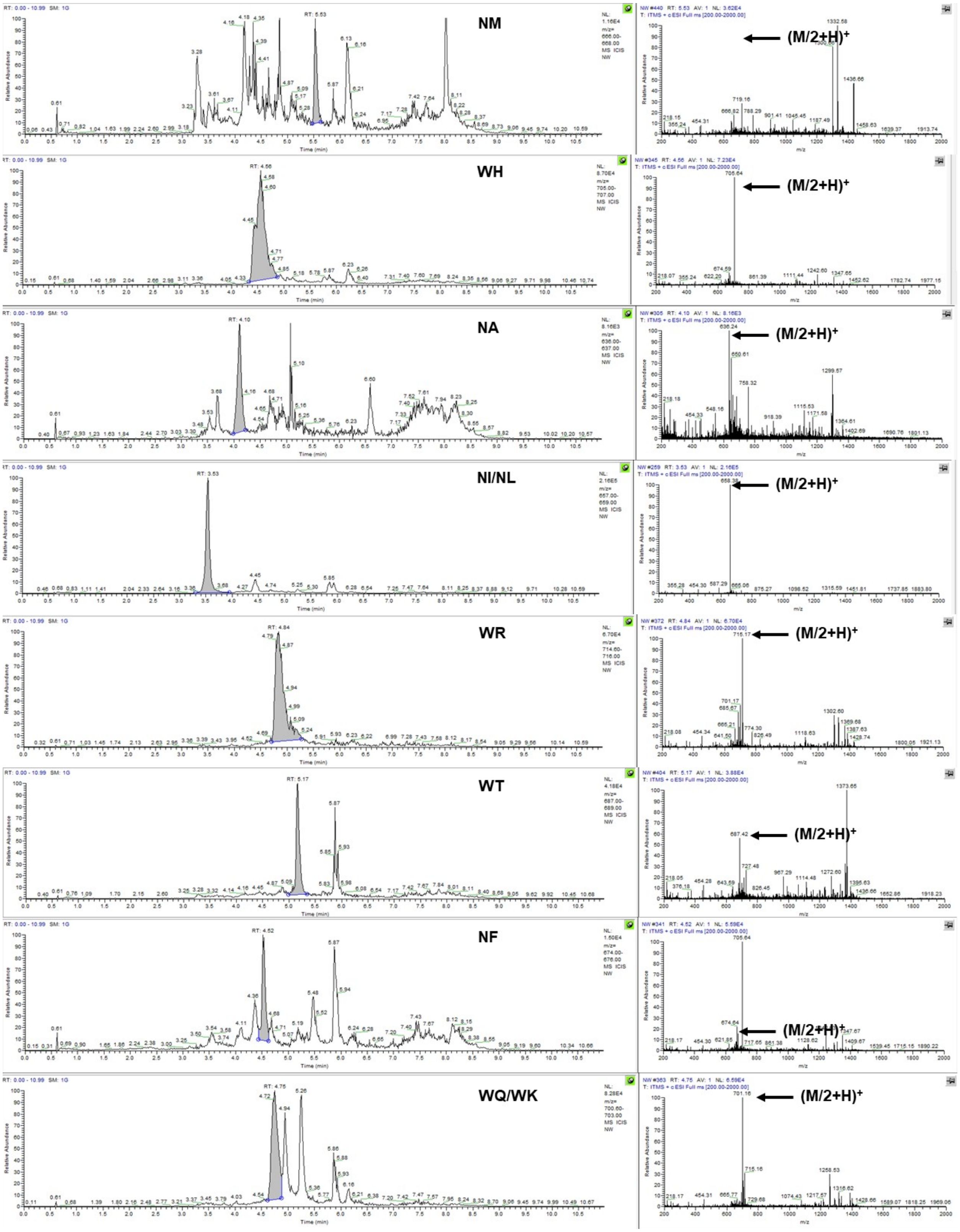
NM
(M/2+H)+
WH
(M/2+H)+
NA
(M/2+H)+
NI/NL
(M/2+H)+
WR
(M/2+H)+
WT
(M/2+H)+
NF
(M/2+H)+
WQ/WK
(M/2+H)+

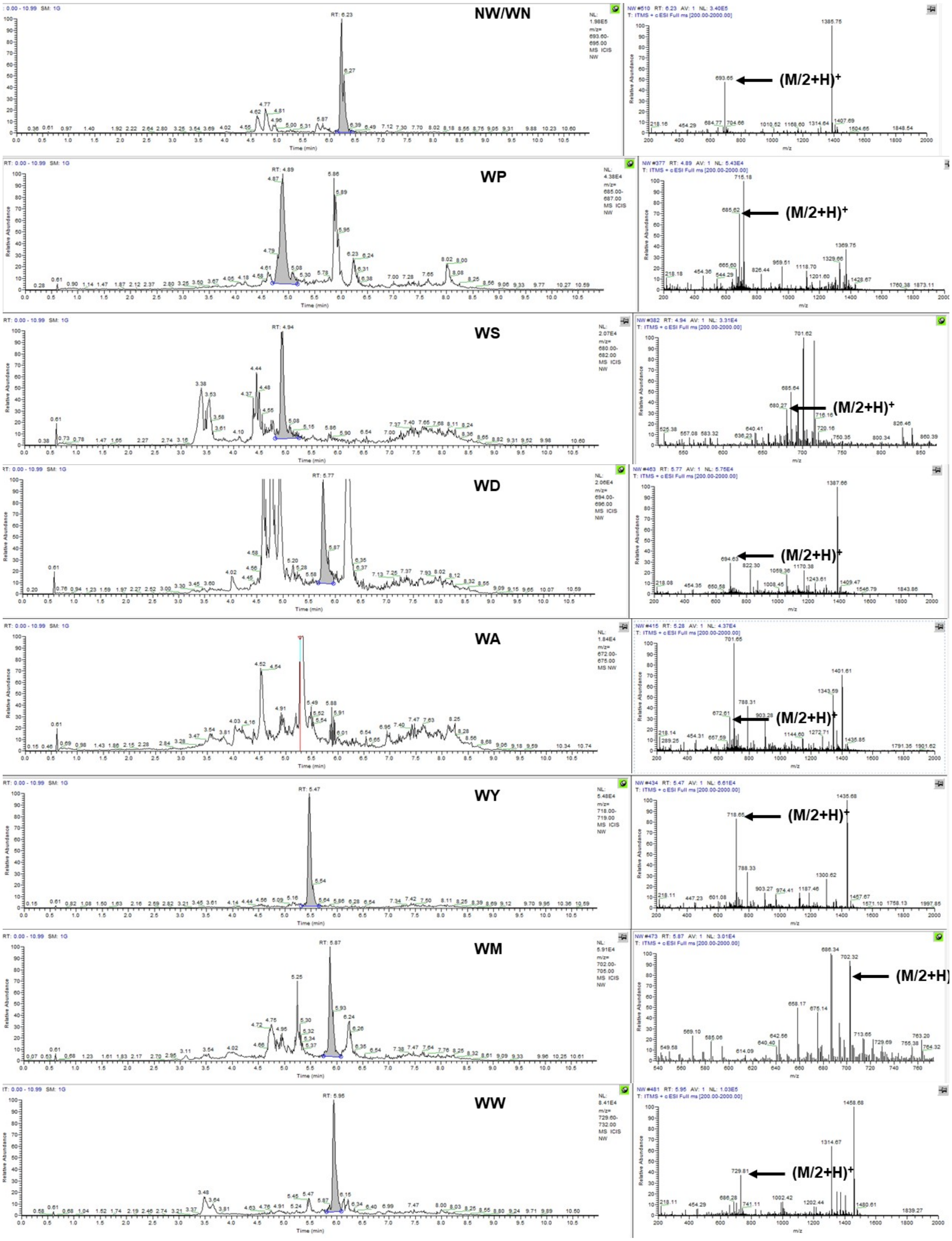
NW/WN
(M/2+H)+
WP
(M/2+H)+
WS
(M/2+H)+
WD
(M/2+H)+
WA
(M/2+H)+
WY
(M/2+H)+
WM
(M/2+H)
WW
(M/2+H)+
Time (min)
m/z
Relative Abundance

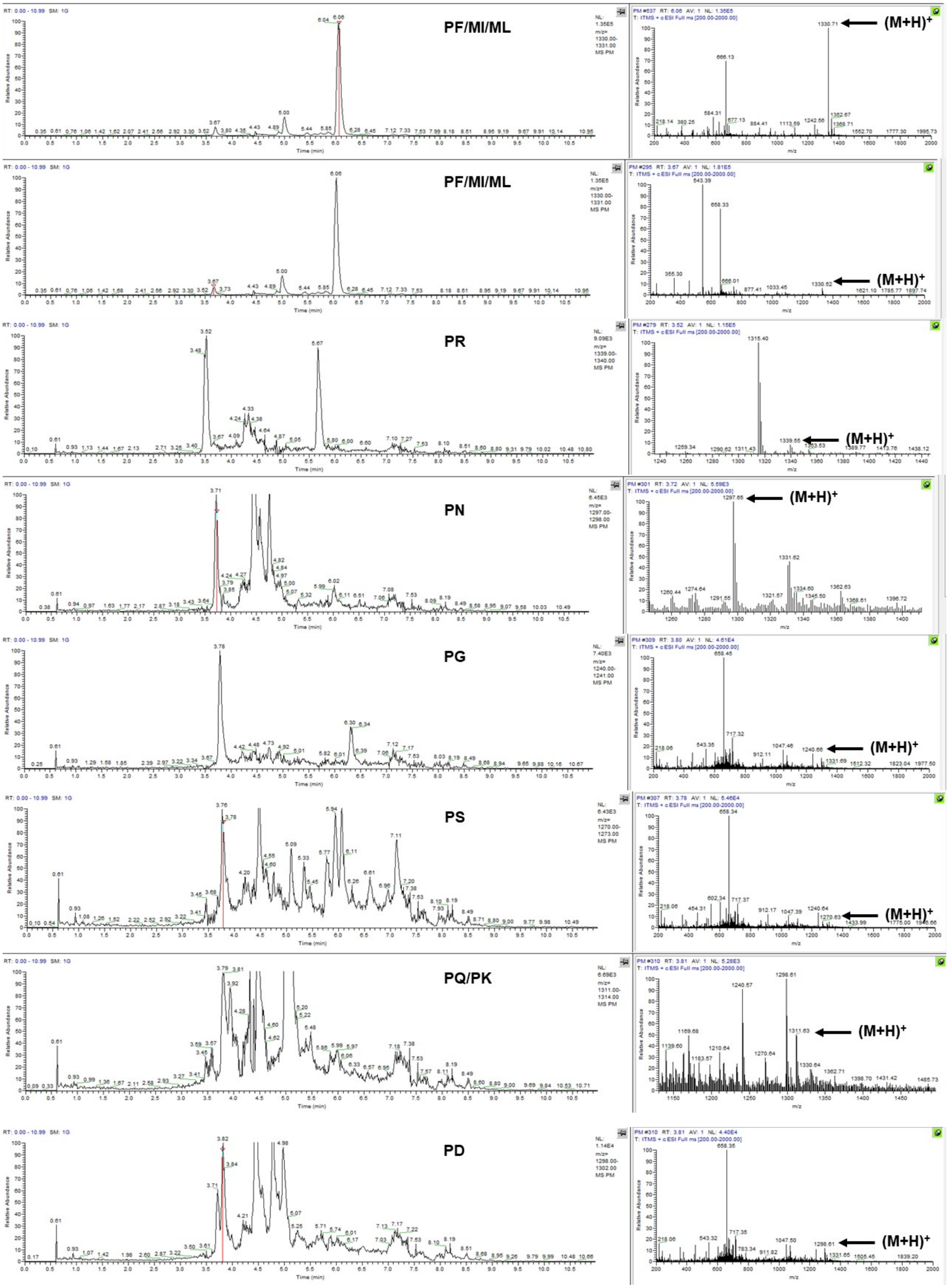

PF/MI/ML
(M+H)+
PF/MI/ML
(M+H)+
PR
(M+H)+
PN
(M+H)+
PG
(M+H)+
PS
(M+H)+
PQ/PK
(M+H)+
PD
(M+H)+
Relative Abundance
Time (min)
m/z

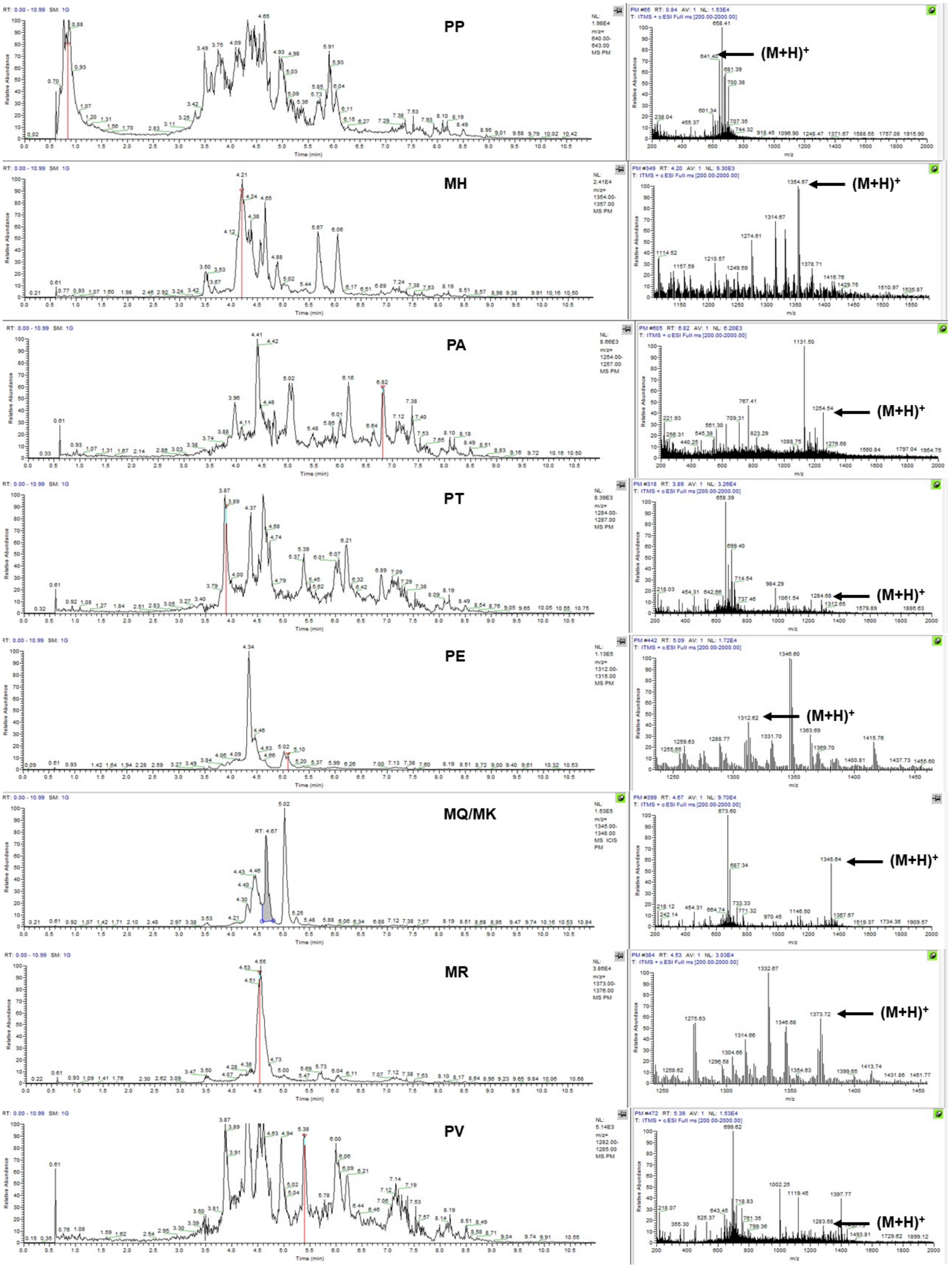
PP
(M+H)+
MH
(M+H)+
PA
(M+H)+
PT
(M+H)+
PE
(M+H)+
MQ/MK
(M+H)+
MR
(M+H)+
PV
(M+H)+
Relative Abundance
Time (min)
m/z

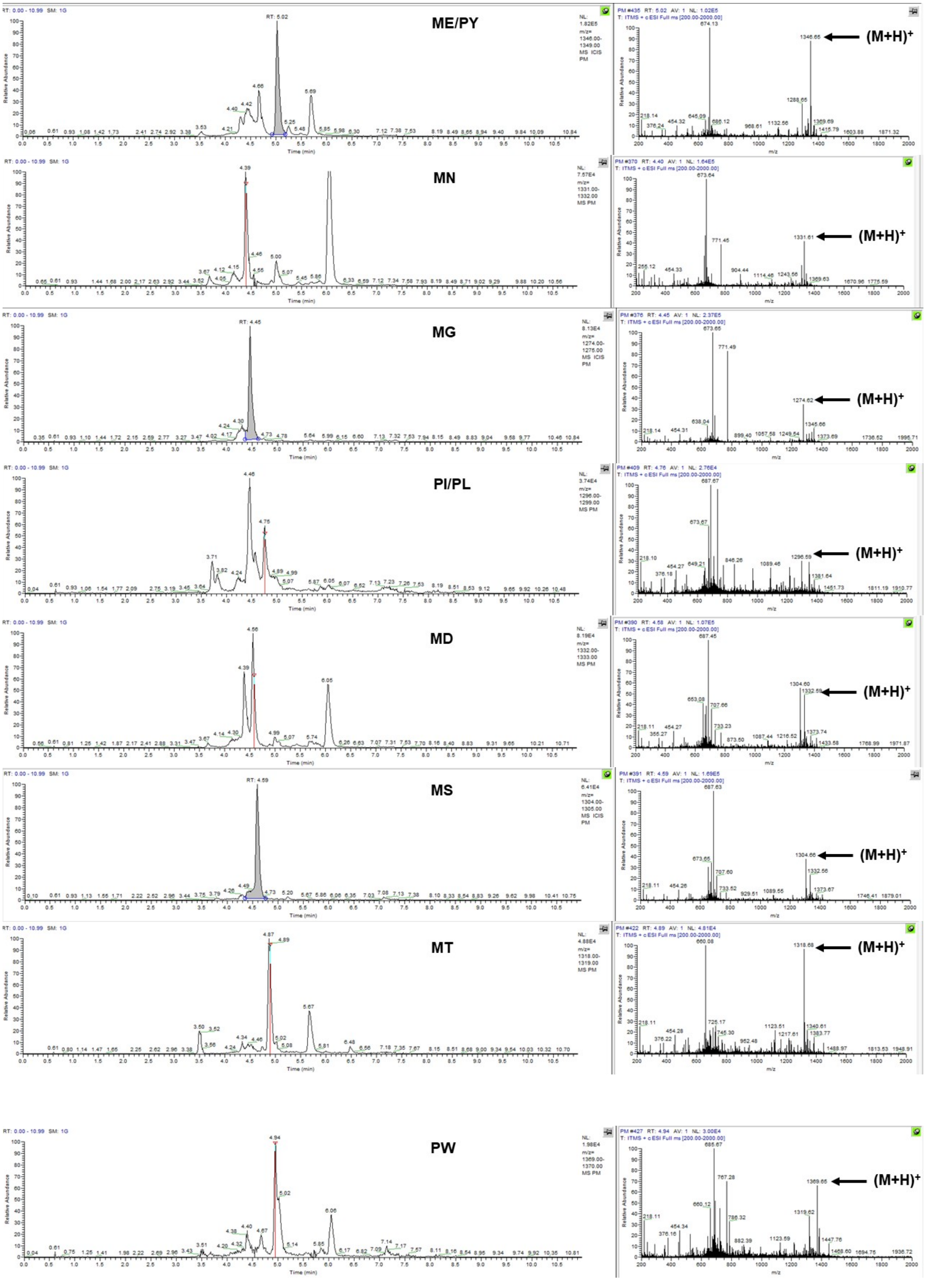
ME/PY
(M+H)$^+$
MN
(M+H)$^+$
MG
(M+H)$^+$
PI/PL
(M+H)$^+$
MD
(M+H)$^+$
MS
(M+H)$^+$
MT
(M+H)$^+$
PW
(M+H)$^+$

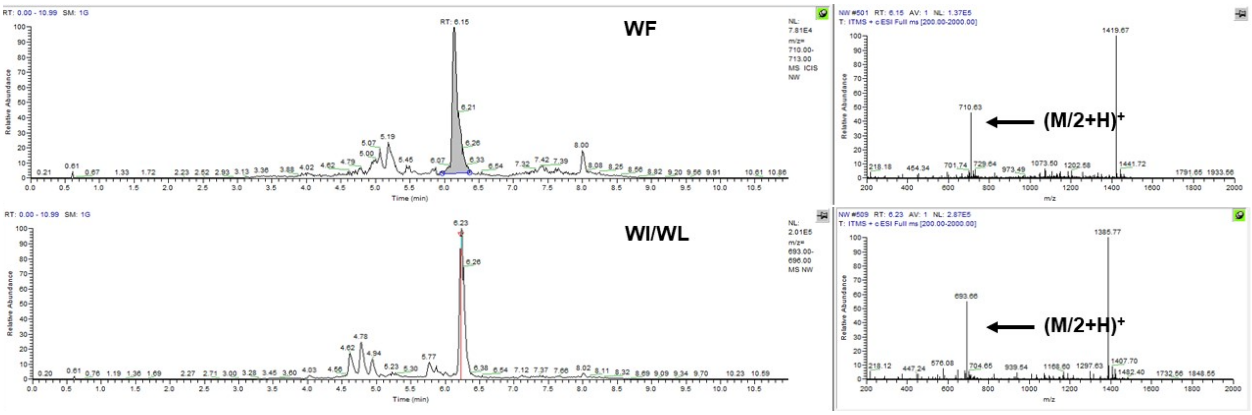
WF
WI/WL
(M/2+H)+
(M/2+H)+
Relative Abundance
Time (min)
m/z

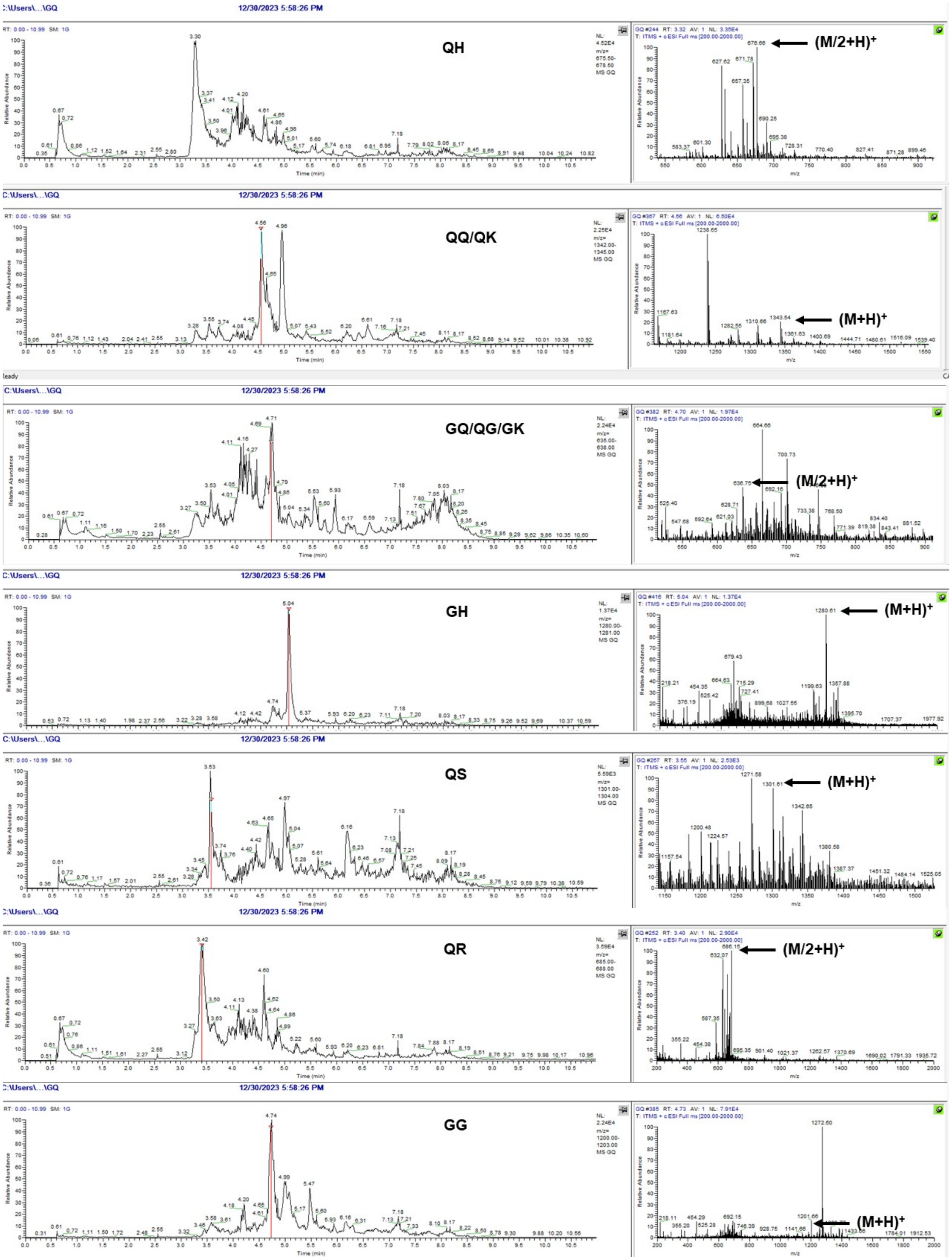

QH
(M/2+H)+
QQ/QK
(M+H)+
GQ/QG/GK
(M/2+H)+
GH
(M+H)+
QS
(M+H)+
QR
(M/2+H)+
GG
(M+H)+

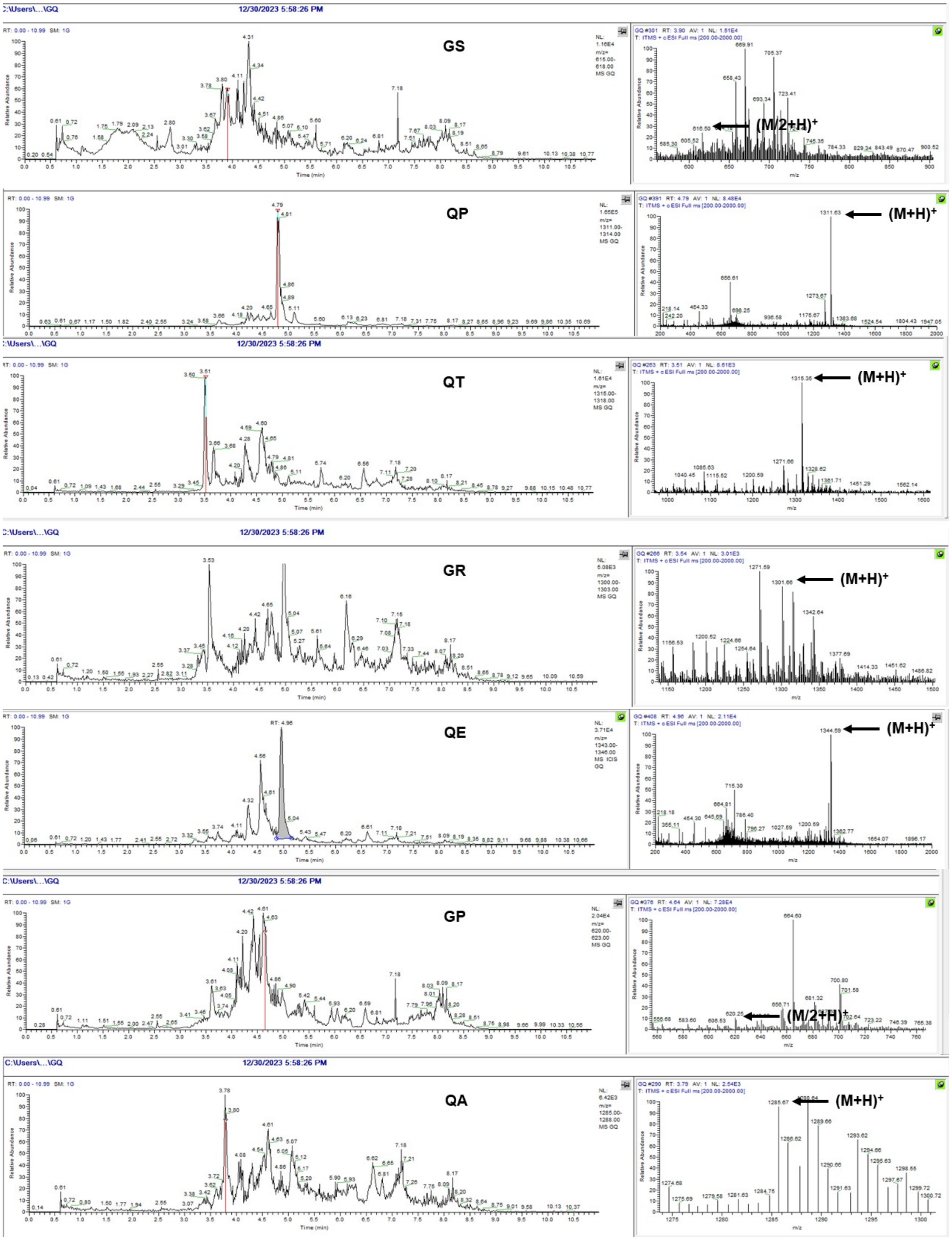
C:\Users\...\GQ
12/30/2023 5:58:26 PM
GS
(M/2+H)+
QP
(M+H)+
QT
(M+H)+
GR
(M+H)+
QE
(M+H)+
GP
(M/2+H)+
QA
(M+H)+
Time (min)
Relative Abundance
m/z

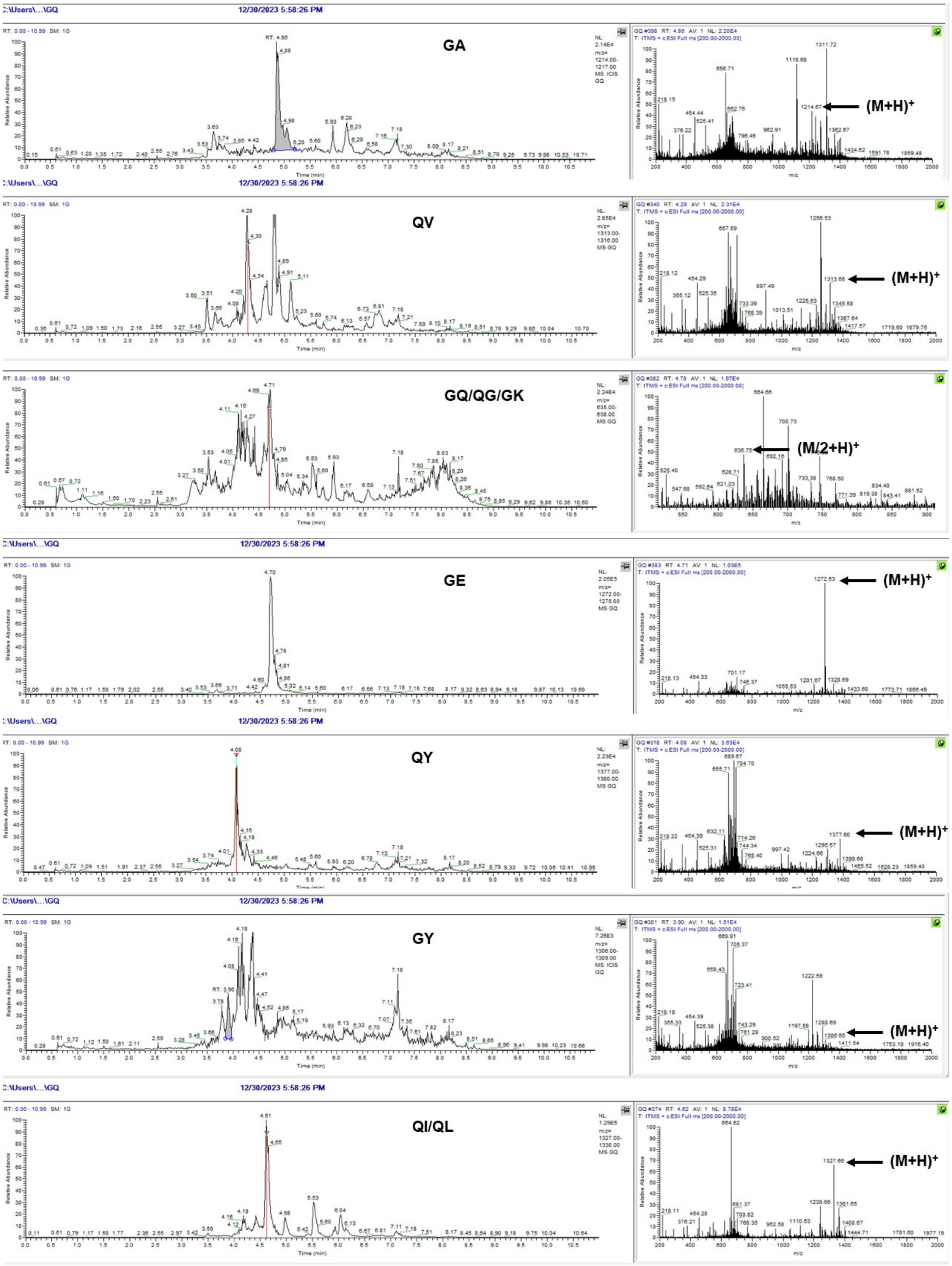
GA
(M+H)+
QV
(M+H)+
GQ/QG/GK
(M/2+H)+
GE
(M+H)+
QY
(M+H)+
GY
(M+H)+
QI/QL
(M+H)+

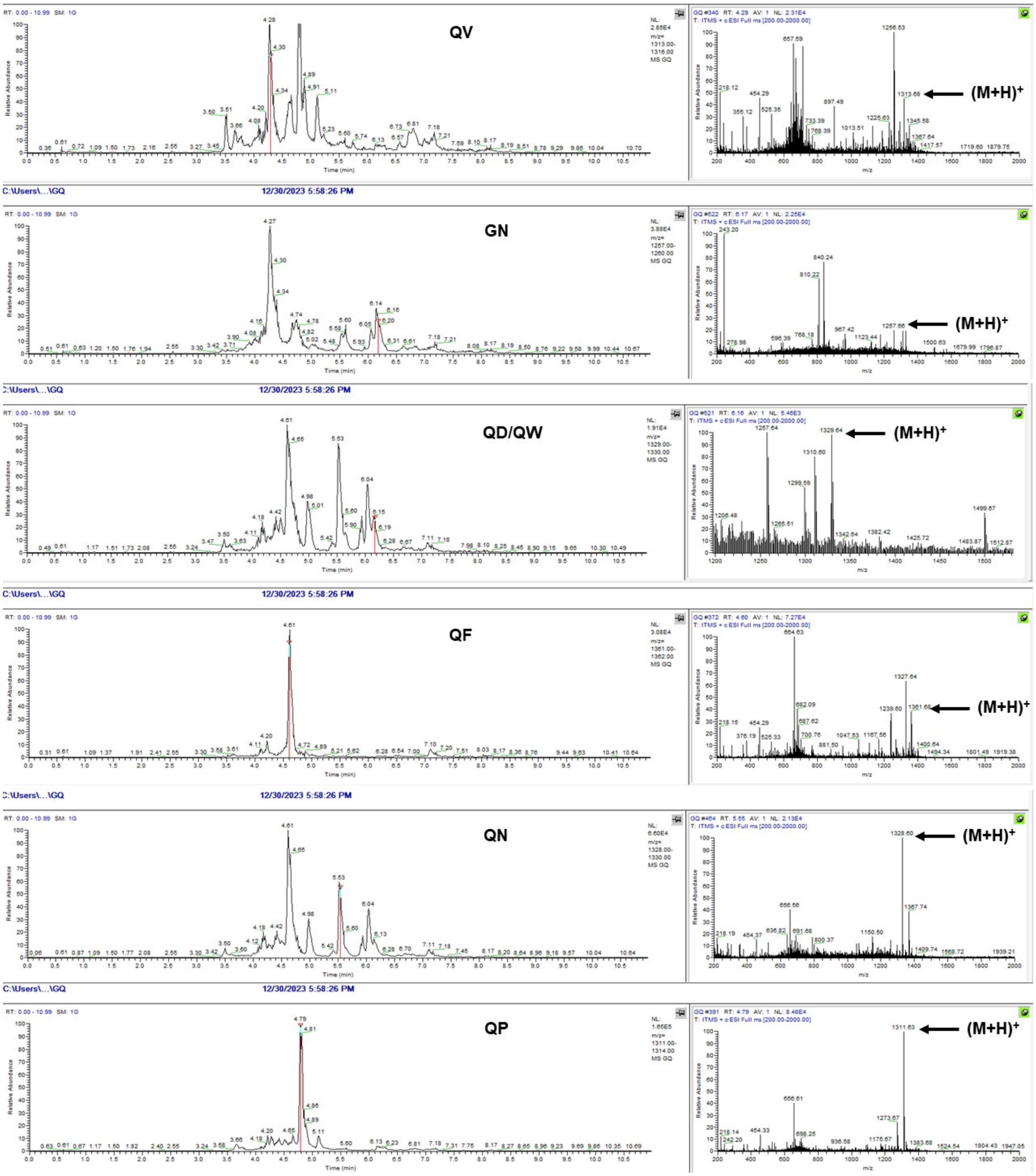
QV
(M+H)+
C:\Users\...\GQ
12/30/2023 5:58:26 PM
GN
(M+H)+
C:\Users\...\GQ
12/30/2023 5:58:26 PM
QD/QW
(M+H)+
C:\Users\...\GQ
12/30/2023 5:58:26 PM
QF
(M+H)+
C:\Users\...\GQ
12/30/2023 5:58:26 PM
QN
(M+H)+
C:\Users\...\GQ
12/30/2023 5:58:26 PM
QP
(M+H)+

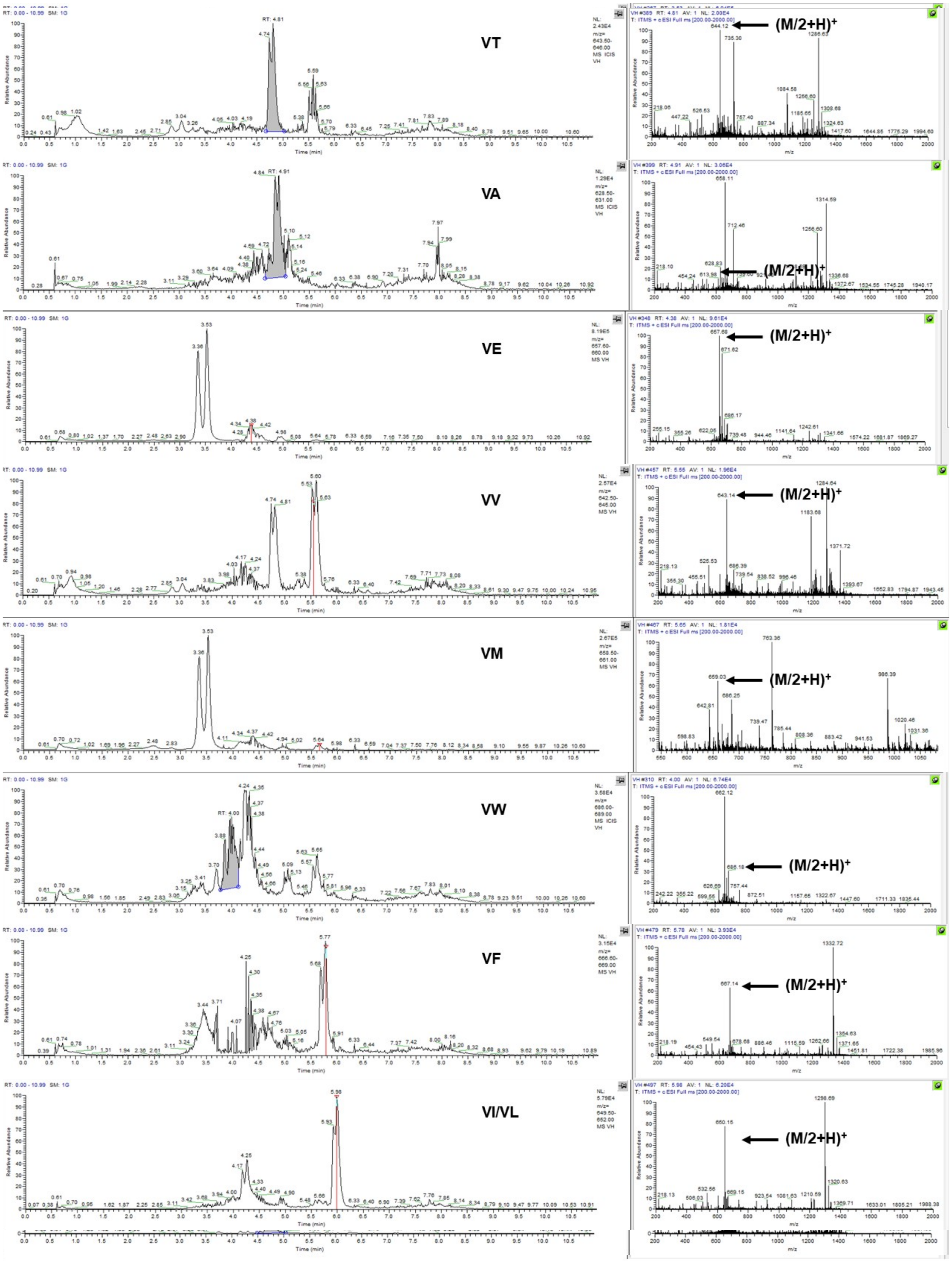
VT
(M/2+H)+
VA
(M/2+H)+
VE
(M/2+H)+
VV
(M/2+H)+
VM
(M/2+H)+
VW
(M/2+H)+
VF
(M/2+H)+
VI/VL
(M/2+H)+

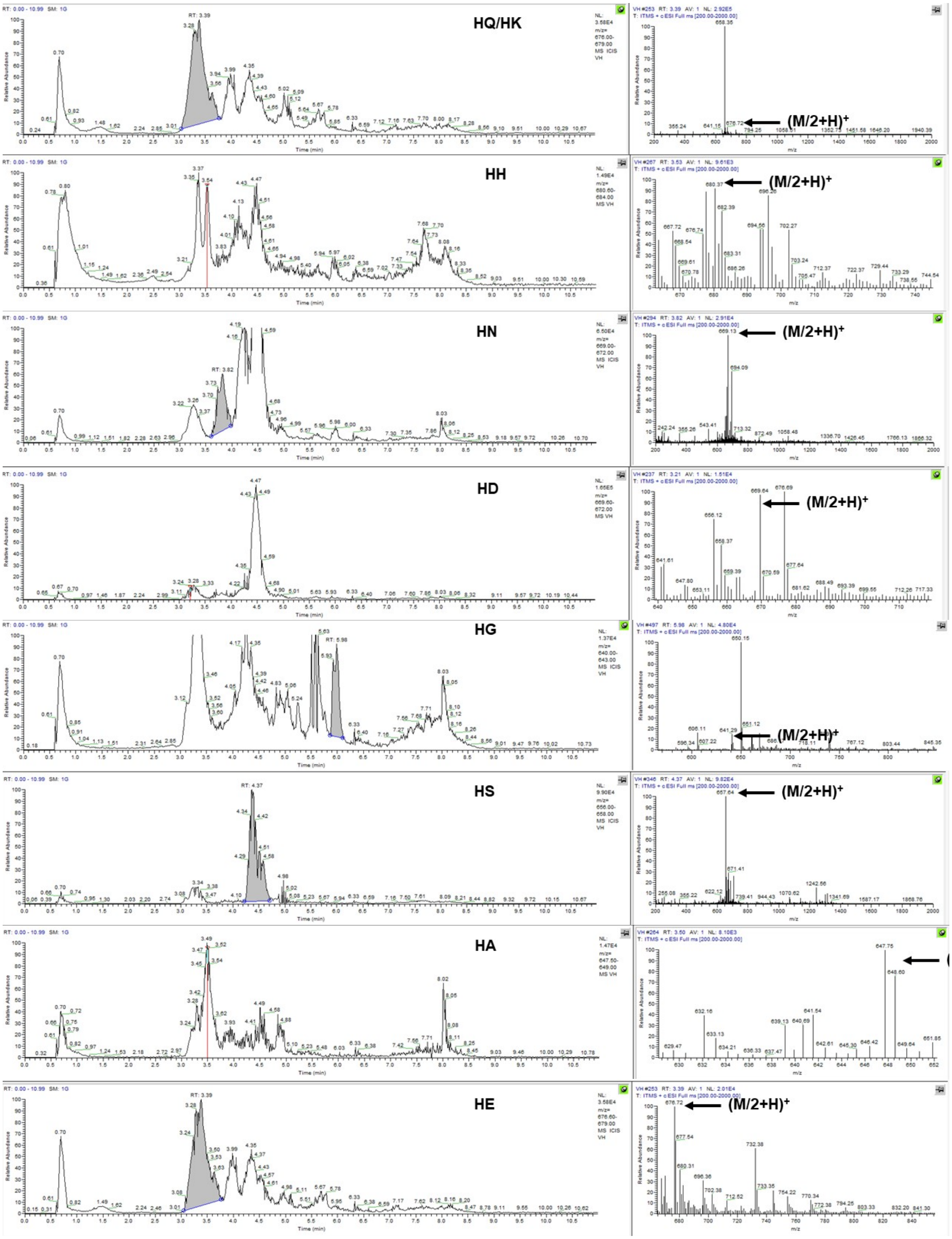

Fig. S9. Mass spec characterization of split-and-pool peptide libraries.
